# Effect of N-Acetylcysteine in Mitochondrial Function, Redox Signaling, and Sirtuin 3 Levels in the Heart During Cardiorenal Syndrome Type 4 Development

**DOI:** 10.3390/antiox14030367

**Published:** 2025-03-20

**Authors:** Isabel Amador-Martínez, Omar Emiliano Aparicio-Trejo, Ana Karina Aranda-Rivera, Bismarck Bernabe-Yepes, Omar Noel Medina-Campos, Edilia Tapia, Carlo César Cortés-González, Alejandro Silva-Palacios, Francisco Javier Roldán, Juan Carlos León-Contreras, Rogelio Hernández-Pando, Emma Saavedra, José Guillermo Gonzaga-Sánchez, Zeltzin Alejandra Ceja-Galicia, Laura Gabriela Sánchez-Lozada, José Pedraza-Chaverri

**Affiliations:** 1 Posgrado en Ciencias Biológicas, Unidad de Posgrado, Edificio D, 1º Piso, Circuito de Posgrados, Ciudad Universitaria, Coyoacán 04510, Mexico; 2 Laboratorio F-315, Departamento de Biología, Facultad de Química, Universidad Nacional Autónoma de México, Mexico City 04510, Mexico; 3 Departamento de Fisiopatología Cardio-Renal, Instituto Nacional de Cardiología Ignacio Chávez, Mexico City 14080, Mexico; 4 Departamento de Farmacología, Facultad de Medicina, Universidad Nacional Autónoma de México, Mexico City 04510, Mexico; 5 Departamento de Biomedicina Cardiovascular, Instituto Nacional de Cardiología Ignacio Chávez, Mexico City 14080, Mexico; 6 Unidad de Investigación Biomédica en Cáncer, Instituto Nacional de Cancerología, Mexico City 14080, Mexico; 7 Departamento de Consulta Externa, Instituto Nacional de Cardiología Ignacio Chávez, Mexico City 14080, Mexico; 8 Experimental Pathology Section, National Institute of Medical Sciences and Nutrition ‘‘Salvador Zubirán’’, Mexico City 14000, Mexico; 9 Departamento de Bioquímica, Instituto Nacional de Cardiología Ignacio Chávez, Mexico City 14080, Mexico

**Keywords:** chronic kidney disease, cardiorenal syndrome type 4, oxidative stress, mitochondrial dysfunction, redox signaling, sirtuin 3

## Abstract

Type 4 cardiorenal syndrome (CRS-4) is a pathology in which chronic kidney disease (CKD) triggers the development of cardiovascular disease. CKD pathophysiology produces alterations that can affect the bioenergetics of heart mitochondria, causing oxidative stress and reducing antioxidant glutathione (GSH) levels. GSH depletion alters protein function by affecting post-translational modifications such as S-glutathionylation (RS-SG), exacerbating oxidative stress, and mitochondrial dysfunction. On the other hand, N-acetylcysteine (NAC) is an antioxidant GSH precursor that modulates oxidative stress and RS-SG. Moreover, recent studies have found that NAC can activate the Sirtuin 3 (SIRT3) deacetylase in diseases. However, the role of NAC and its effects on mitochondrial function, redox signaling, and SIRT3 modifications in the heart during CRS-4 have not been studied. This study aimed to investigate the role of NAC in mitochondrial function, redox signaling, and SIRT3 in the hearts of animals with CRS-4 at two months of follow-up. Our results showed that the oral administration of NAC (600 mg/kg/day) improved blood pressure and reduced cardiac fibrosis. NACs’ protective effect was associated with preserving cardiac mitochondrial bioenergetics and decreasing these organelles’ hydrogen peroxide (H_2_O_2_) production. Additionally, NAC increased GSH levels in heart mitochondria and regulated the redox state, which coincided with an increase in nicotinamide adenine dinucleotide oxidized (NAD^+^) levels and a decrease in mitochondrial acetylated lysines. Finally, NAC increased SIRT3 levels and the activity of superoxide dismutase 2 (SOD-2) in the heart. Thus, treatment with NAC decreases mitochondrial alterations, restores redox signaling, and decreases SIRT3 disturbances during CRS-4 through an antioxidant defense mechanism.

## 1. Introduction

Chronic kidney disease (CKD) is a severe and progressive health condition characterized by the gradual deterioration of kidney function over time. Regardless of its cause, the pathophysiology of CKD manifests as glomerulosclerosis and/or interstitial fibrosis due to renal inflammation, tubular atrophy, capillary rarefaction, and the deposition of an extracellular matrix [1]. The global estimated prevalence is between 10 and 12%, and it is expected to increase due to its association with a variety of risk factors such as diabetes, hypertension, obesity, and aging [1,2,3]. Notably, CKD patients develop cardiovascular complications through hemodynamic, neurohormonal, and inflammatory factors [4,5]. The association between CKD and cardiovascular disease (CVD) is classified as cardiorenal syndrome type 4 (CRS-4), whose prevalence varies from 5.5 to almost 80% and is the leading cause of death in patients with CKD [6,7].

Although in CRS-4 the pathophysiological mechanisms are poorly understood [6,8], oxidative stress emerges during CKD and is detrimental for the heart [9]. Oxidative stress is produced by the imbalance between antioxidants and reactive oxygen species (ROS) in favor of the latter [10]. Even during the moderate stages of CKD, the heart overproduces ROS, tipping the delicate balance towards oxidative stress [11]. In addition, mitochondria play an essential role in the heart’s functions; however, they are also considered one of the primary sources of ROS production, promoting cellular injury [10,12]. Mitochondrial function is maintained by a balanced redox buffering capacity of the glutathione (GSH) antioxidant [13]. Therefore, the change in the redox state modulates protein function by the oxidation of protein cysteine thiols, like S-glutathionylation (RS-SG). In this way, mitochondrial proteins, such as sirtuin 3 (SIRT3) deacetylase and the electron transport system (ETS), contain reactive thiol groups susceptible to redox regulation, affecting their functions [14,15,16].

Previous studies have shown that GSH levels significantly decrease in CKD and worsen as kidney function is lost [17]. In addition, enzymatic RS-SG is induced by increased hydrogen peroxide (H_2_O_2_) levels [13,15] and by GSH depletion, which occurs during CKD [13,18]. N-acetylcysteine (NAC) is an antioxidant approved by the Food and Drug Administration for treating acetaminophen overdoses and some lung diseases [19,20]. It is also a GSH precursor that modulates RS-SG in CKD and CRS-3 [18,21]. Moreover, NAC has been found to produce the activation of mitochondrial SIRT3 deacetylase (a crucial deacetylase found in the mitochondrial matrix) in diabetic nephropathy [22] and during renal toxicity [23], decreasing ROS levels and improving mitochondrial membrane potential (ΔΨm). However, it is not known how mitochondrial function, redox signaling, and SIRT3 are affected in the heart during CRS-4 development and whether the long-term oral administration of NAC (600 mg/kg/day) can modulate GSH levels and reduce mitochondrial dysfunction, redox signaling, and SIRT3 alterations of the heart in a clinically relevant model of CRS-4 [24,25]. Thus, the present study aimed to evaluate whether treatment with NAC during CKD can restore GSH levels in cardiac mitochondria and whether, in this way, NAC maintains redox signaling and regulates mitochondrial alterations and the SIRT3 deacetylase in CRS-4.

## 2. Materials and Methods

### 2.1. Reagents

Adenosine 5′-diphosphate (ADP) sodium salt, adenosine 5′-triphosphate (ATP) sodium salt, Amplex red, antimycin A, ammonium chloride (NH_4_Cl), bromophenol blue, fat-free bovine serum albumin (BSA), β-mercaptoethanol, 1-chloro-2,4-dinitrobenzene (CDNB), catalase from bovine liver, cytochrome c from equine heart, coenzyme A (CoA), Coomassie Brilliant blue G 250 (for Bradford reagent), D-mannitol, decylubiquinone (DUB), dipotassium phosphate (KH_2_PO_4_), 2,6-dichlorophenolindophenol sodium salt hydrate (DCPIP), 5,5′-dithio-bis-(2-nitrobenzoic acid) (DTNB), lyophilized powder, 5-thio-2-nitrobenzoic acid (TNB) ethylene glycol-bis(2-aminoethyl ether)-N,N,N′,N′-tetraacetic acid (EGTA), GSH, glutathione disulfide (GSSG), glutathione peroxidase (GPx), glutathione reductase (GR), glutaraldehyde, glycerol, 4-(2-hydroxyethyl)-1-piperazineethanesulfonic acid (HEPES), horseradish peroxidase (HRP), K-lactobionate, lead citrate, manganese (II) chloride (MgCl_2_) tetrahydrate, malic acid, NAC, N-ethyl maleimide (NEM), Nicotinamide adenine dinucleotide phosphate reduced (NADPH), oxidized (NADP^+^), Nicotinamide adenine dinucleotide reduced (NADH), oxidized (NAD^+^), NADH/NAD^+^ assay kit (MAK037), nitro blue tetrazolium (NBT), non-fat dry milk, olygomicin, osmium tetroxide, paraformaldehyde, phenylmethanesulfonyl fluoride (PMSF), protease and phosphatase inhibitor cocktail, potassium cyanide (KCN), rotenone, sodium azide (NaN_3_), sodium bicarbonate (NaHCO_3_), sodium chloride (NaCl) sodium deoxycholate, sodium dihydrogen phosphate monobasic (NaH_2_PO_4_), sodium dodecyl sulfate (SDS), sodium glutamate, sodium hydroxide (NaOH), sodium L-ascorbate, sodium fluoride (NaF), sodium malate, sodium orthovanadate (Na_3_VO4), sodium succinate dibasic, superoxide dismutase (SOD) from bovine source, sucrose, taurine, tetramethyl-p-phenylenediamine (TMPD), trizma (Tris), trizma-hydrochloride (Tris-HCl), triton X-100, thiamine pyrophosphate, tween, uranyl acetate, and 2-vinylpyridine (2-VP) were purchased from Sigma-Aldrich (St. Louis, MO, USA). Commercial kits from Spinreact (Girona, Spain) were used to measure blood urea nitrogen (BUN) and plasma creatinine. Sodium bicarbonate (NaHCO_3_), H_2_O_2_, ethyl alcohol, ethylenediaminetetraacetic acid (EDTA) disodium salt dihydrate, and potassium hydroxide (KOH) were purchased from JT Baker (Xalostoc, Edo. Mexico, Mexico). Epoxy resin was purchased from Pelco International (Redding, CA, USA). Sedative sodium pentobarbital (SedalphorteMR) was purchased from Salud y Bienestar Animal S.A. de C.V. (Mexico City, Mexico).

### 2.2. Ethics Statement

Rats were obtained from the animal facility center of the Institute of Cellular Physiology of the Universidad Nacional Autónoma de México. All experimental procedures were approved by the Institutional Animal Care and Use Committee of the Instituto Nacional de Cardiología Ignacio Chávez (INC/CICUAL/011/2023). The animal procedures followed the guidelines for the Care and Use of Laboratory Animals published by the Mexican Official Norm Guides (NOM-062-ZOO-2001) and the National Institutes of Health. The disposal of biological residues was performed following NOM-087- SEMARNAT-SSA-2002.

### 2.3. Experimental Design

**Figure 1:**
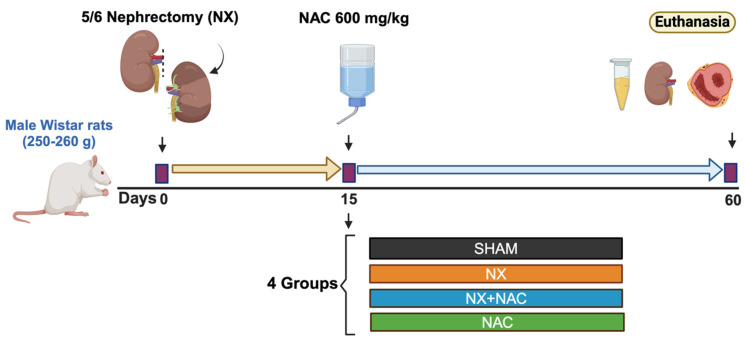
Experimental protocol. Male Wistar rats weighing 250–260 g were subjected to 5/6 Nephrectomy (NX) or SHAM surgery. At day 15 of the surgery, rats were given N-acetylcysteine (NAC) in the vehicle (a combination of drinking water and a 300 mM carbonate buffer) at 600 mg/kg/day. Rats were divided into 4 groups (SHAM, NX, NX + NAC, and NAC), as represented in the rectangles below. All animals were followed for 60 days until euthanasia. SHAM: simulated surgery + vehicle; NX: 5/6 Nephrectomy + vehicle; NX + NAC: 5/6 Nephrectomy treated with N-acetylcysteine; NAC: control healthy rats treated with N-acetylcysteine.

Seventy-eight male Wistar rats (*Rattus norvegicus*) weighing 250 to 260 g were randomly assigned to four experimental groups (Figure 1): (1) SHAM: animals that underwent laparotomy (n = 18). (2) 5/6 Nephrectomy (NX): rats subjected to a right unilateral nephrectomy, followed by the selective infarction of approximately 2/3 parts of the left kidney on the same day [26,27] (n = 22). SHAM and NX groups were administered with the vehicle starting on day 15 post-surgery and ending at 60 days. (3) NX + NAC: rats undergoing 5/6 Nephrectomy, with daily oral NAC administration (600 mg/kg/day) starting 15 days after the surgery and continuing for 45 additional days (n = 22). Thus, the treatment lasted until day 60 (Figure 1). NAC was given at 15 days because uremia and systemic oxidative stress are already evident in NX rats. To ensure complete NAC consumption, rats were placed in individual cages and given a daily dose adjusting the pH to 7.2 (with NaOH 10%) and the volume to 25 mL with a combination of drinking water and a 300 mM carbonate buffer (vehicle). After receiving the dose, the rats were returned to their cages with access to tap water. (4) NAC: animals without surgical intervention but administered NAC following the same temporal schedule as that of the NX + NAC group (n = 16).

Rats were maintained in standard laboratory conditions (24 ± 1°C temperature, 40 ± 5% humidity, and a 12:12 h light–dark cycle) and provided free access to a standard rodent laboratory diet (5001, Rodent Diet^TM^). A 12 h pre-surgery fasting period was implemented to facilitate the surgical process. The dose of NAC was chosen based on previous studies [27,28,29] and according to our preliminary studies showing a cardiac protective effect in rats at two months follow-up of CKD. After receiving the dose, the rats were returned to their cages with access to tap water. At the end of this study (60 days), rats were subjected to euthanasia with a lethal dose of sodium pentobarbital intraperitoneally (120 mg/kg). Then, rats were placed on a heating pad to maintain body temperature at 37 °C, and the plasma, heart, and remnant kidney were collected (Figure 1).

### 2.4. Renal Damage Markers (BUN, Creatinine, and Proteinuria)

To verify renal damage after surgery and before NAC treatment, 0.4 milliliters of blood was taken from the tail vein and collected into heparinized tubes after 24 h and 15 days. Blood samples were centrifuged at 3000× *g* for 10 min at 4 °C to obtain plasma and kept at −70 °C until used. To measure renal damage markers at the end of the experimental follow-up, a blood sample was taken from the abdominal aorta and centrifuged at 3000× *g* for 10 min to obtain plasma. Commercial kits from Spinreact (Girona, Spain) were used to measure BUN (510 nm) and creatinine (492 nm) following the manufacturer’s instructions. Values were compared with those reported in the literature for the same strain of rats [30,31].

Rats were individually placed in metabolic cages to collect 24 h urine at 0, 15, 30, and 60 days to determine urinary protein excretion. Urine at 60 days was also used to assess creatinine levels and calculate its clearance [32]. Proteinuria levels were quantified using the Bradford method [33]. For this, the urine was passed through different dilutions with distilled water, and upon determining the appropriate one, it was quantified with the Bradford reagent according to the absorbance measurement at 595 nm. The value was interpolated to a BSA standard curve with known concentrations. Urinary protein excretion was reported as mg per urinary volume collected in 24 h.

### 2.5. Systolic Blood Pressure (SBP)

SBP was measured noninvasively in conscious restrained rats with a tail-cuff sphygmomanometer (IN125/R, ADI Instruments, Colorado Spring, CO, USA) [30] on days 0, 15, 30, and 60. For this, whole rats were placed in an incubator at 40 °C for 5 min to dilate the tail vein [30]. Then, the same operator made three measurements, and we reported the mean in each experimental group.

### 2.6. Histology Evaluation

Histological analyses of cardiac and renal tissues [hematoxylin–eosin (H&E) and Masson’s trichrome] were performed following established protocols [31,34]. For fibrosis quantification, left ventricle (LV) sections were stained with Masson’s trichrome. Then, the fibrotic area of eight different sections of the tissue of three animals was obtained concerning the total area, and the percentage of fibrosis was quantified with the same adjustment parameters with ImageJ software (v 1.53, National Institutes of Health, Bethesda, MD, USA). The quantification was reported as a percentage of the average fibrosis.

### 2.7. Protein Extraction and Western Blot (WB) Assay

Protein extraction and WB assay were made as previously described [34]. Briefly, 30 μg of protein was loaded in SDS–polyacrylamide gel electrophoresis (PAGE) for 1 h and 30 min at 120 volts. The gels were transferred to polyvinylidene fluoride (PVDF) membranes and then blocked with non-fat dry milk for 1 h at room temperature. Then, membranes were incubated overnight with corresponding primary antibodies prepared in Tris-buffered saline with 0.1% Tween 20 detergent, using the dilutions shown in Supplementary Table S1. Secondary antibodies (Supplementary Table S2) were incubated in constant stirring at room temperature and darkness for 1 h and 30 min. The protein bands were identified using fluorescence in an Odyssey Sa scanner (LICOR Biosciences, Lincoln, NE, USA) or chemiluminescence in ChemiDoc Imaging Systems (Bio-Rad, Hercules, CA, USA). Bands were analyzed with ImageJ studio, and protein quantification was expressed as arbitrary units, representing the optical density ratio of the protein of interest/loading control. Glyceraldehyde 3-phosphate dehydrogenase (GAPDH) was used as a loading control for kidney proteins, and actin was used as a loading control for heart proteins. 

### 2.8. Evaluation of Cardiac Function by Echocardiography (Echo)

Two-dimensional cardiac chamber images were obtained from the short-axis views of the left ventricle (LV) at the papillary muscle level of at least 3 beats in each rat of the different groups using a SONOS 550 echocardiographer (Koninklijke Phillips Electronics, Eindhoven, The Netherlands) with a 12 MHz transducer. The rats were anesthetized with sodium pentobarbital at a dose of 1.9 mg/100 g body weight intraperitoneally [34]. The LV internal diameter, LV posterior wall thickness, fractional shortening (FS), and % ejection fraction (%EF) were calculated from the LV dimensions at the end-systole (LVDs) and end-diastole (LVDd) using Teicholz’s formula as follows: %EF = (Vd − Vs)/Vd, where Vd = [7/(2.4 + LVDd)] × LVDd3 and Vs = [7/(2.4 + LVDs)] × LVDs3 [34]. At the end of the evaluation, the animals recovered for a few days before being euthanized.

### 2.9. OXPHOS Subunit Protein Content in Total Heart Tissue

The protein levels of the OXPHOS subunits were evaluated by WB using an antibody cocktail (Supplementary Table S1). The cocktail comprises a mixture of five antibodies, each detecting CI-CIV complexes and ATP synthase subunits [35]. The protein subunits are NADH: ubiquinone oxidoreductase subunit B8 (NDUFB8) for CI, succinate dehydrogenase B (SDHB) for CII, ubiquinol–cytochrome c reductase core protein 2 (UQCRC2) for CIII, cytochrome c oxidase I for CIV, and ATP 5A for the ATP synthase. The homogenates for the immunodetection of OXPHOS were handled according to the manufacturer’s instructions (Abcam, Cambridge, UK).

### 2.10. Mitochondrial Isolation and Oxygen Consumption

After sacrifice, the heart’s LV was immediately cooled by immersion in isolation buffer (225 mM D-mannitol, 75 mM sucrose, 1 mM EDTA, 5 mM HEPES, 0.1% BSA, pH = 7.4) at 4 °C and then cut into small pieces. The tissues were homogenized in a glass Potter-Elvehjem with a Teflon VR pestle in the same buffer, and mitochondria were obtained as previously reported [36]. The mitochondrial pellet was resuspended in 200 μL of BSA-free isolation buffer, and the mitochondrial total protein was measured using the Lowry method [37].

Mitochondrial oxygen (O_2_) consumption was evaluated at 37 °C with a high-resolution respirometer (Oxygraph O2k, OROBOROS, Innsbruck, Austria) following established protocols [38,39]. The values were corrected by the total protein content.

### 2.11. Mitochondrial Complexes’ Activity in Total Heart Tissue

The spectrophotometric evaluation of the mitochondrial complexes I, II, and IV involved 20 μg of total protein at 37 °C. This analysis used a Synergy HT microplate reader (Biotek Instruments, Winooski, VT, USA), as previously described [38]. Moreover, 160 μM DCPIP was reduced in a reaction with 60 μM of decyl ubiquinone (DUB) in 80 μM NADH to measure CI activity. The CI activity was determined by the proportional disappearance of the oxidized DCPIP at 600 nm in the presence of 80 μM NADH, 2 μM antimycin A, 2.5 mM malonate, and 5 mM NaN_3_. In a parallel assay, 1 mM rotenone was added to obtain the non-specific reaction. CII activity was assessed in 20 mM succinate, 2 μg/mL rotenone, 2 μg/mL antimycin A, and 5 mM NaN_3_. The reduction of DCPIP by DUB was followed at 600 nm. The non-specific reaction was determined by adding 1 M malonate. The CIII activity involved monitoring the generation of the reduced form of cytochrome c at 550 nm, using 3.12 mM DUBH_2_ in the presence of 500 μM KCN, 1 μM rotenone, 5 mM NaN_3_, and 75 μM oxidized cytochrome c. The non-specific cytochrome c reduction was evaluated by adding 1.8 mM antimycin A. The activity of CIV was based on monitoring the oxidation of 1 mM reduced cytochrome c at 550 nm, with 5 mM KCN and 1.5 μm antimycin A. The non-specific oxidation of cytochrome c was determined by adding 5 mM NaN_3_. All activities were expressed as a percentage of change versus the control.

### 2.12. Mitochondrial H_2_O_2_ Production

The levels of H_2_O_2_ were measured in an O2k-Fluorometer (OROBOROS, Innsbruck, Austria) with Amplex red as a probe. The isolated mitochondria were resuspended in 2.0 mL of MiR05 (MiR05 plus HRP 0.5 U/mL). Calibration curves with 0.1 μM steps of H_2_O_2_ were performed to ensure the assay’s linearity states [39]. The H_2_O_2_ production rate in respiratory states was determined as state 2 H_2_O_2_ production, obtained by adding mitochondria plus CI- and CII-linked substrates (5 mM pyruvate, 2 mM malate, 10 mM glutamate plus 10 mM succinate). The production in state 3 was measured after adding 2.5 mM ADP. Finally, 2.5 μM oligomycin was added to determine the production in state 4. All results were normalized by protein content.

### 2.13. Glutathione Content in the Total Homogenate, Isolated Mitochondria, and Mitochondrial Protein RS-SG in the LV of the Heart

One hundred mg of the LV was homogenized in 0.5 mL of phosphate buffer (0.1 M K_2_HPO_4_, 5 mM disodium EDTA, pH = 7.5). Samples were divided into two aliquots of 0.250 mL each to measure either total glutathione (GSH + GSSG) or GSSG. GSH + GSSG and the glutathione disulfide (GSSG) content was determined using the method described by Rahman et al. [40]. The rate of absorbance change for each experiment was compared with the standards of GSH or GSSG.

The levels of S-glutathionylated proteins were determined in isolated mitochondria using an antibody that identifies this modification by WB (Supplementary Table S1). Before mitochondrial isolation, the buffer was treated with 50 mM N-ethyl maleimide (NEM). NEM is a sulfhydryl alkylating reagent that blocks free cysteines and avoids their oxidation [41]. The steps for mitochondrial isolation are shown in one section above.

### 2.14. Transmission Electron Microscopy

Small tissue fragments of the heart’s LV were fixed with Karnovsky fixative (1.5% glutaraldehyde and 4% paraformaldehyde) in 0.2 M Sörensen buffer at pH = 7.4 to analyze their ultrastructural electron microscopy [36]. The tissues were then post-fixed with 1% osmium tetroxide, dehydrated with ethyl alcohol in ascending concentrations, and infiltrated in epoxy resin. Finally, ultrathin sections were contrasted with uranyl acetate and lead citrate and observed with an electron microscope (Tecnai Spirit BioTwin, FEI, Hillsboro, OR, USA).

### 2.15. NAD^+^/NADH Measurements in Heart Tissue

NAD^+^/NADH levels were analyzed with a colorimetric assay following the manufacturer’s instructions (MAK037, Sigma-Aldrich, St. Louis, MO, USA). An NADH standard curve with known concentrations corrected the data. The Lowry method determined the protein content, which normalized all the results [37].

### 2.16. Activity of Antioxidant Enzymes in Heart Tissue

The activity of antioxidant enzymes in heart tissue was measured according to a previous report [18]. One hundred mg of tissue from the LV was homogenized into 50 mM phosphate buffer containing 1% triton X-100 and centrifuged at 15,000× *g* for 10 min at 4 °C. The supernatant was recovered to determine superoxide dismutase-2 (SOD-2) activity by spectrophotometry by monitoring at 560 nm the NBT reduction to formazan as a probe [42]. One unit of SOD-2 activity was identified as the protein level that inhibits NBT reduction at 50% [43]. The activity of SOD-1 was inhibited with 0.1 M of NaN_3_. Catalase activity was evaluated at 240 nm following the Aebi method based on the H_2_O_2_ decomposition [44]. GPx activity was determined by the disappearance of NADPH at 340 nm in a coupled reaction with GR [45]. GR activity was measured at 340 nm by evaluating the decrease in NADPH absorbance at 340 nm [46]. Glutathione S-transferase (GST) activity was assessed by measuring the increase in absorbance at 340 nm generated by the adduct GSH-CDNB.

### 2.17. Statistical Analysis

The results are reported as mean ± standard error of the mean (SEM). Data analysis was performed with GraphPad Prism 8 (v. 8.2.1, GraphPad Software, San Diego, CA, USA). Shapiro–Wilk and Normal QQ plots were applied to determine whether parametric or nonparametric tests should be conducted. Comparisons among groups were tested with one-way ANOVA or Kruskal–Wallis tests followed by their post hoc analysis. Significant differences were considered when the *p* value was < 0.05.

## 3. Results

### 3.1. Administration of NAC-Reduced CKD-Induced Cardiac Alterations at Two-Month Follow-Up

**Figure 2:**
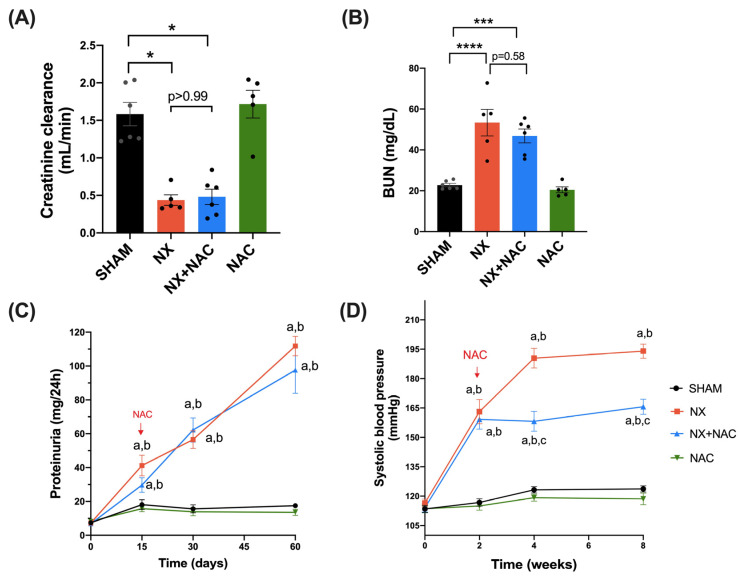
N-acetylcysteine administration reduced functional and structural renal damage markers. (**A**) Creatinine clearance; (**B**) blood urea nitrogen (BUN) in the plasma at two months; (**C**) proteinuria (mg/24 h); and (**D**) systolic blood pressure (SBP) in weeks 2, 4, and 8 after NX were evaluated in all experimental groups. Data are mean ± SEM, analyzed by one-way ANOVA followed by Tukey’s post hoc test (**B**–**D**) and Kruskal–Wallis followed by Dunn’s multiple comparison tests (**A**). n = 5–10: individual values are denoted with black dots for each group. In graphs A and B, * *p* < 0.05, *** *p* < 0.001, and **** *p* < 0.0001. In graphs C and D, a = *p* < 0.05 vs. SHAM (black line), b = *p* < 0.05 vs. NAC (green line), c = *p* < 0.05 vs. NX (orange line). SHAM: simulated surgery + vehicle; NX: 5/6 Nephrectomy + vehicle; NX + NAC: 5/6 Nephrectomy treated with N-acetylcysteine; NAC: control healthy rats treated with N-acetylcysteine.

We first validated the CKD model’s efficacy by evaluating the parameters indicative of kidney function. BUN and creatinine in plasma at 24 h and 15 days after NX were increased compared to those in the SHAM group (Supplementary Figure S1A–D), showing similar damage in the nephrectomized groups. At that point, we administered NAC (600 mg/kg) through drinking water; therefore, NAC was administered for 45 days (Figure 1). At 60 days post-nephrectomy, we observed a significant decrease in creatinine clearance and increased BUN levels in the NX group, which were not recovered in the NX + NAC group (Figure 2A,B). The chronic administration of NAC did not demonstrate a renal impairment in normal rats (NAC group). In the SHAM and NAC groups, proteinuria remained at its baseline levels from the beginning to the end of this study (Figure 2C). However, it increased from the second to the fourth week in the NX (111.8 ± 5.8 mg/24 h) and the NX + NAC (97.6 ± 13.7 mg/24 h) groups, indicating CKD maintenance. The H&E-stained kidney sections showed large focal areas of proximal convoluted tubular damage and atrophy manifested by flattened epithelial cells with some necrotic cells, granular hyaline casts, and interstitial chronic inflammatory infiltrates surrounded by fibrosis and small arteries and arterioles with a hyperplastic middle layer in the NX group, which was confirmed by Masson’s trichrome-stained sections. All these histological abnormalities did not decrease in the NX + NAC group (Supplementary Figure S1E).

An intrinsic consequence of the pathophysiology of CKD is hypertension, which may lead to cardiac dysfunction and worsens CKD. In our study, we observed a statistically significant increase in systolic blood pressure (SBP) in the NX and the NX + NAC groups from the second week of follow-up that was maintained until 8 weeks (Figure 2D). We confirmed that the NX group displayed a significant elevation in SBP at follow-up (Figure 2D, 194 ± 3.6). On the contrary, in the NX + NAC group we observed a partial and significant decrease in SBP (Figure 2D, 166 ± 3.87), highlighting NAC as a potential strategy for cardiovascular hemodynamic alterations.

**Figure 3:**
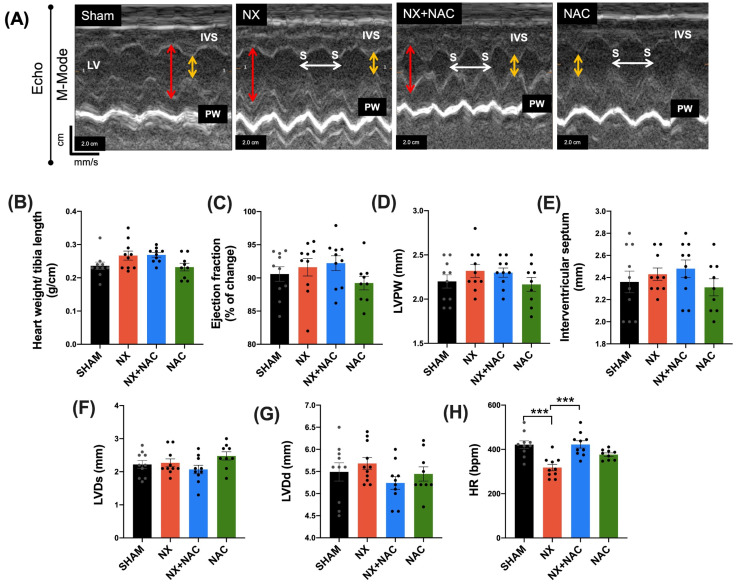
Echocardiography and functional parameters in rats subjected to 5/6 Nephrectomy (NX) and treated with N-acetylcysteine (NAC). (**A**) Representative images of the Echo. M-mode at the mid-ventricular level from two-dimensional images. To measure the end-systolic (yellow double-headed arrow) and end-diastolic (red double-headed arrow) diameters of the left ventricle (LV), the thickness of the interventricular septum (IVS), and the posterior wall (PW) and to calculate the heart rate, the distance between two consecutive systoles (S, white double-headed arrow) was evaluated. (**B**–**H**) Heart weight/tibia length, %EF, LVPW, IVS, LVDs, LVDd, and HR. Data are presented as the mean ± SEM of 9–10 animals in each experimental group. The analysis was performed using ANOVA followed by Tukey’s post hoc test (**A**,**D**,**E**,**G**,**H**) or Kruskal–Wallis followed by Dunn’s multiple comparison tests (**B**,**C**,**F**). *** *p* < 0.001. EF: ejection fraction; HR: heart rate; LVDd: left ventricular dimension at end-diastole; LVDs: left ventricular dimension at end-systole; LVPW: left ventricular dimension posterior wall; bpm: beats per minute. SHAM: simulated surgery + vehicle; NX: 5/6 Nephrectomy + vehicle; NX + NAC: 5/6 Nephrectomy treated with N-acetylcysteine; NAC: control healthy rats treated with N-acetylcysteine.

To evaluate the cardiac function, echocardiography was conducted 60 days after NX (Figure 3). We found that NX group displayed a significant reduction in heart rate (HR) (Figure 3H, *p* < 0.001) compared with that of the SHAM group; meanwhile, no changes in the heart weight/tibia length rate, the %EF, the left ventricular posterior wall (LVPW), the interventricular septum (IVS), the left ventricular end-systole (LVDs), and the end-diastole (LVDd) were seen (Figure 3A–G). The NX + NAC group showed an evident improvement in HR compared to that of the NX group (Figure 3H, *p* < 0.001).

**Figure 4:**
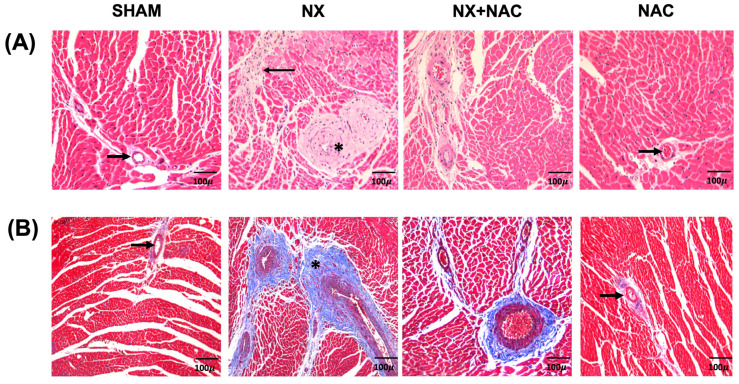

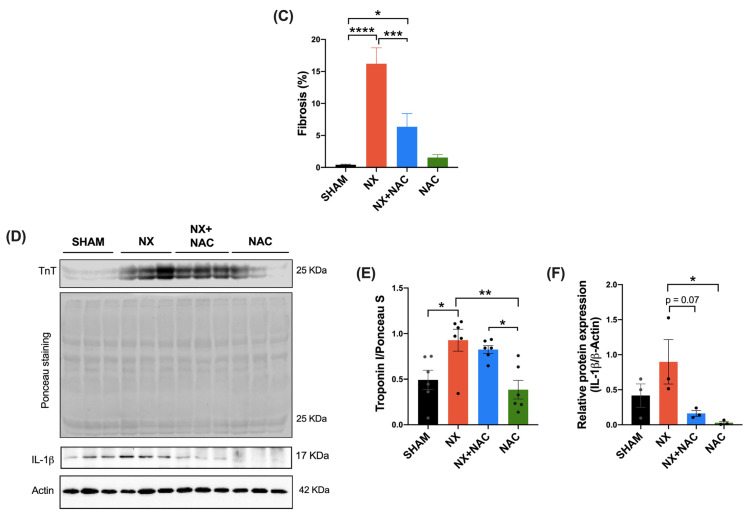
Therapeutic effects of NAC in cardiovascular alterations during 5/6 Nephrectomy (NX)-induced chronic kidney disease (CKD). (**A**) Representative hematoxylin and eosin (H/E) staining micrographs of the heart’s left ventricle. In comparison with the SHAM and NAC groups that show normal myocytes and arteries (arrows), the NX section shows a large widening of the arterial walls (asterisks) and fibrotic bands that invade and substitute the myocardium (arrow); these histological abnormalities are lesser in the NX + NAC group. (**B**) Masson’s trichrome staining micrographs of the SHAM and NAC groups showing vascular layers with scarce collagen tissue stained in blue (arrow). In contrast, the NX group shows extensive perivascular fibrosis (asterisk) with wide bands invading the neighboring myocardial tissue, whereas this perivascular fibrosis is much less in the NX + NAC group. Scale bar = 100 μm of magnification. (**C**) These differences in fibrosis were confirmed by automated morphometry using the heart sections stained with Masson’s trichrome where the NX group showed threefold more fibrosis than that of the NX + NAC group. (**D**,**E**) The immunoblot and protein quantification of cardiac troponin (TnT) in plasma proteins. Ponceau staining was used as a loading control: (**D**,**F**) immunoblot and relative protein expression of interleukin-1β (IL-1β). Actin was used as a loading control. Data are presented as mean ± SEM analyzed by one-way ANOVA followed by Tukey’s test. Individual values are denoted with black dots for each group. * *p* < 0.05, ** *p* < 0.01, *** *p* < 0.001, and **** *p* < 0.0001. SHAM: simulated surgery + vehicle; NX: 5/6 Nephrectomy + vehicle; NX + NAC: 5/6 Nephrectomy treated with N-acetylcysteine; NAC: control healthy rats treated with N-acetylcysteine.

Histological evaluation with H&E and Masson’s trichrome stainings were conducted to study cardiac structure alterations such as remodeling. The results showed significant abnormalities in the cardiac tissue of the NX group compared to that of the SHAM manifested by considerable widening of the medium arterial layer by fibrosis, which substantially decreased the vascular lumen; this perivascular fibrosis highly extended and substituted the neighbor cardiac muscular tissue (Figure 4A). As expected, the percentage of the fibrotic area in the myocardium was significantly increased in the LV of the NX group (16.22%) compared to that of the SHAM group (0.4%). Notably, the NX + NAC group significantly decreased this parameter (6.35%) (Figure 4B,C). Plasma troponin T (TnT) is a systemic marker for evaluating cardiac dysfunction [47]. Therefore, we performed the immunodetection of TnT in the plasma of animals at 8 weeks after NX. As shown in Figure 4D,E, the TnT protein levels significantly increased (*p* < 0.01) in the plasma of animals of the NX group compared to that of the SHAM group, but it partially decreased in the NX + NAC animals (*p* = 0.946). This result showed that NAC exerted partial protection during CKD, possibly as previously reported, by reducing systemic inflammatory events and hemodynamic changes [48,49]. Because cardiorenal damage is highly promoted by persistent inflammation [25], we evaluated the proinflammatory cytokine interleukin-1β (IL-1β) in the LV of the heart. As shown in Figure 4D,F, the levels of IL-1β notably increased in the heart of the NX group compared to that of the SHAM group without significant changes (*p* = 0.30). Interestingly, IL-1β levels decreased further in the NX + NAC group treated with NAC (*p* = 0.07), suggesting a cardiac anti-inflammatory effect promoted by NAC.

### 3.2. NAC Recovered the CKD-Induced Decrease in Heart ETS Activity, OXPHOS Capacity, and Mitochondrial Decoupling During CRS-4

**Figure 5:**
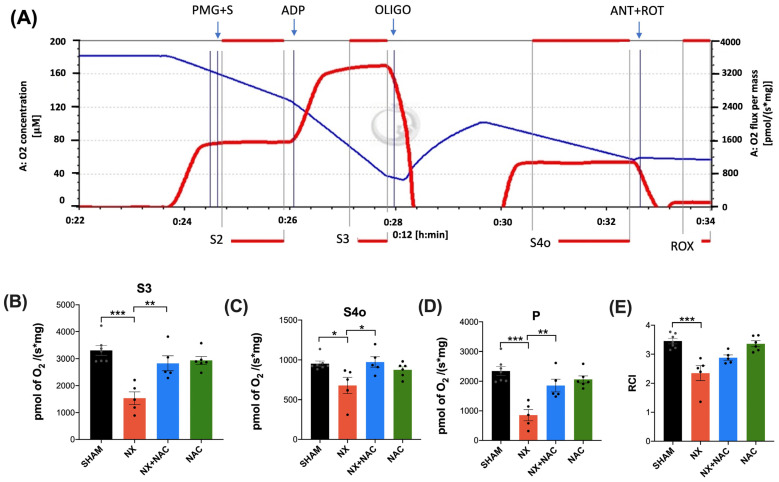
Respiratory states in the cardiac mitochondria of animals with CRS-4 development. Mitochondrial states: (**A**) Schematic representations of the methods used to determine the respiratory parameters. The blue line shows oxygen (O_2_) concentration (μM). Red plots: O_2_ consumption rate normalized per milligram of protein [pmol/(s*mg)]. PMG= pyruvate, malate, and glutamate; S = succinate; ADP = adenosine triphosphate; OLIGO = oligomycin; ANT = antimycin A; ROT = rotenone. (**B**) State 3 (S3), (**C**) state 4 induced by oligomycin (S4o), (**D**) OXPHOS-associated respiration (P), and (**E**) respiratory control index (RCI) in CI + CII-linked respiration. Data are presented as mean ± SEM analyzed by one-way ANOVA followed by Tukey’s test. Individual values are denoted with black dots for each group. * *p* < 0.05, ** *p* < 0.01, and *** *p* < 0.001. SHAM: simulated surgery + vehicle; NX: 5/6 Nephrectomy + vehicle; NX + NAC: 5/6 Nephrectomy treated with N-acetylcysteine; NAC: control healthy rats treated with N-acetylcysteine.

To evaluate if the heart’s mitochondria developed alterations in the capabilities of OXPHOS and if NAC reduced these, we assessed the mitochondrial respiratory parameters in CI + CII-linked respiration (Figure 5A). In the LV-isolated mitochondria of the NX group, there was a significant decrease in the respiratory parameters S3, S4o, P, and RCI (Figure 5B–E). On the contrary, NAC administration during CKD significantly prevented the decrease in the same variables. These data indicated that NAC reversed the NX-induced heart mitochondrial decoupling and OXPHOS impairment.

**Figure 6:**
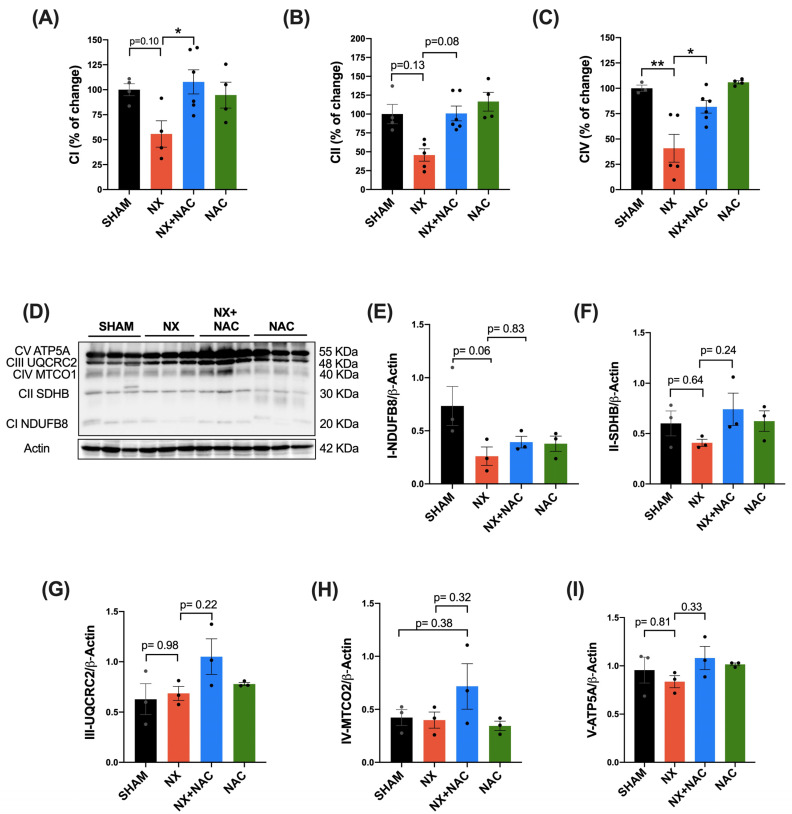
Effect of NAC on mitochondrial complexes’ activity and the levels of subunits of the OXPHOS proteins in the left ventricle (LV) of animals with CRS-4 development. The percentage (%) of activity change compared to that of the SHAM group of the mitochondrial complexes in the LV of hearts is shown in (**A**) complex I (CI), (**B**) complex II (CII), and (**C**) complex IV (CIV). (**D**) The representative immunoblotting and densitometric analysis of the levels of (**E**) NADH–ubiquinone oxidoreductase subunit B8 (CI-NDUFB8), (**F**) succinate dehydrogenase complex iron–sulfur subunit B (CII-SDBH), (**G**) ubiquinol–cytochrome c reductase core protein 2 (CIII-UQCRC2), (**H**) cytochrome c oxidase subunit I (CIV-MTCO1), and (**I**) a subunit of the ATP synthase (CV-ATP5A). Actin was used as a loading control. Data are presented as mean ± SEM analyzed by one-way ANOVA followed by Tukey’s test and Kruskal–Wallis followed by Dunn’s multiple comparison tests (**B**). Individual values are denoted with black dots for each group. * *p* < 0.05 and ** *p* < 0.01. SHAM: simulated surgery + vehicle; NX: 5/6 Nephrectomy + vehicle; NX + NAC: 5/6 Nephrectomy treated with N-acetylcysteine; NAC: control healthy rats treated with N-acetylcysteine.

We assessed the enzymatic activity of the CI, CII, and CIV complexes in the LV of the heart to evaluate whether these effects were related to ETS alteration. The CI, CII, and CIV activities decreased in the LV of the NX group compared with in that of the SHAM group (Figure 6A–C). In contrast, the treatment with NAC protected their activities (Figure 6A–C), which can be associated with its beneficial effect on OXPHOS. Afterward, to establish if the ETS lower activity was related to changes in the complex (CI-CIV) subunit protein contents, this was evaluated by WB (Figure 6D–I). CI-NDUFB8, CII-SDHB, and ATP5A levels decreased in the NX group compared to in the SHAM group without being statistically significant (Figure 6D,F). Interestingly, CIV-MTCO1 and ATP5A protein levels in the NX + NAC group were higher than in the NX group, explaining a possible improvement in ATP production in the heart during CKD (Figure 6D,H,I). These results suggested that NAC could improve OXPHOS function in the heart through mitochondrial mass preservation.

### 3.3. NAC Prevented Increased Mitochondrial H_2_O_2_ Production in Cardiac Mitochondria by Regulating GSH Levels and RS-SG During CRS-4

**Figure 7:**
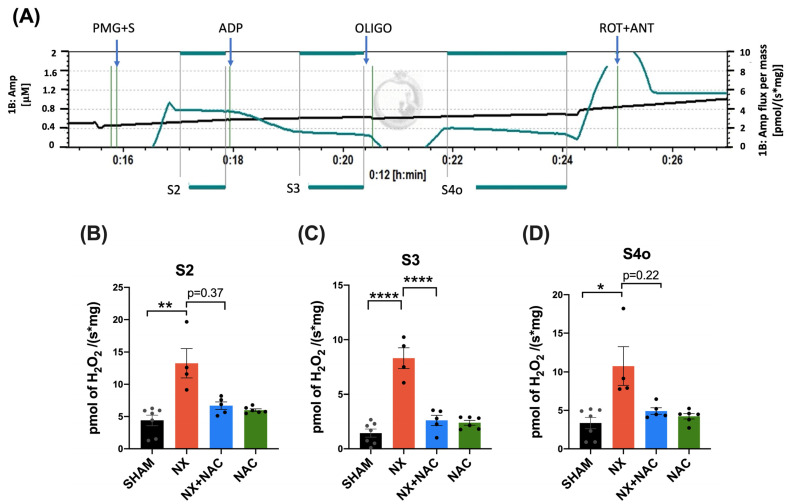

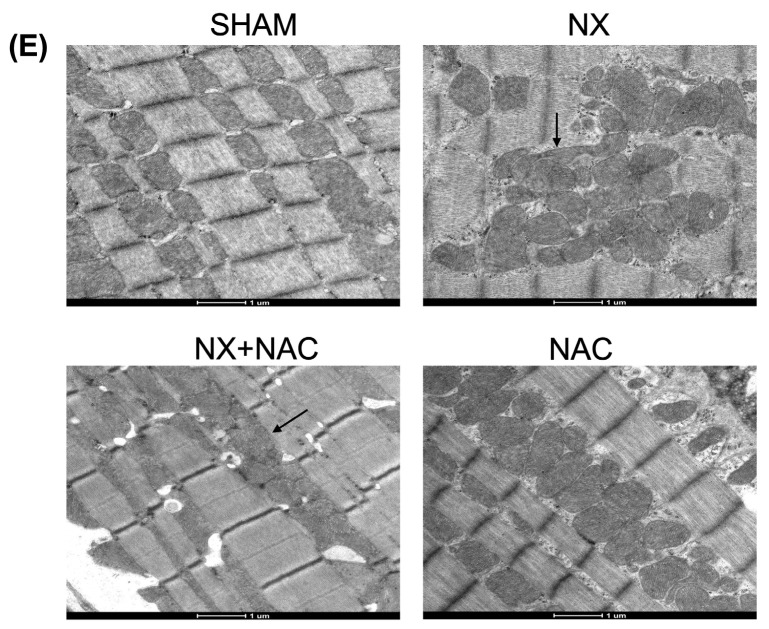
Mitochondrial hydrogen peroxide (H_2_O_2_) production rates and morphological analysis of mitochondria in the heart during CRS-4 development. (**A**) The H_2_O_2_ production rate evaluation in an O2k-Fluorometer (OROBOROS, Innsbruck, Austria) using Amplex red as a probe. The black line represents the total H_2_O_2_ concentration (μM) directly proportional to Amplex red fluorescence. The blue line shows the rate of H_2_O_2_ production in each respiratory state [pmol/(s*mg)]. (**B**) S2 feeding CI + CII, (**C**) S3 feeding CI + CII, and (**D**) S4o feeding CI + CII. CI= complex I; CII= complex II; S2 = state 2; S3 = state 3; S4 = state 4. PMG = pyruvate, malate, and glutamate; S = succinate; ADP = adenosine triphosphate; OLIGO = oligomycin; ANT = antimycin A; ROT = rotenone. Data are presented as the mean ± SEM analyzed by one-way ANOVA followed by Tukey’s test (**B**) and Kruskal–Wallis followed by Dunn’s multiple comparison tests (**A**,**C**). The data of H_2_O_2_ production were corrected by the values of an H_2_O_2_ standard curve. Individual values are denoted with black dots for each group. * *p* < 0.05, ** *p* < 0.01, and **** *p* < 0.0001. (**E**) The representative ultrastructural morphology of mitochondria from cardiomyocytes of the indicated experimental groups. The SHAM and NAC groups showed ovoid mitochondria with well-preserved cristae. In contrast, NX showed disorganized groups of small and round mitochondria with cristae effusion and NX + NAC rats showed long, apparently fused mitochondria (Black arrows). Scale bar= 1 μm of magnification. SHAM: simulated surgery + vehicle; NX: 5/6 Nephrectomy + vehicle; NX + NAC: 5/6 Nephrectomy treated with N-acetylcysteine; NAC: control healthy rats treated with N-acetylcysteine.

To investigate whether NAC prevented mitochondrial bioenergetic alterations through the modulation of the redox balance, we evaluated the mitochondrial H_2_O_2_ production rate (Figure 7A). Mitochondria isolated from the hearts of animals with NX showed higher rates of H_2_O_2_ production in states S2, S3, and S4o compared to the SHAM group (Figure 7B–D). The levels of H_2_O_2_ production were reduced in S2, S3, and S4o in the NX + NAC group. However, the reduction was only significant in S3, indicating that NAC can act as an antioxidant that regulates the redox state in the mitochondria during CRS-4 development.

The increased ROS levels may induce alterations in mitochondrial morphology. Thus, we next characterized the influence of NAC on the structure of mitochondria and cristae morphology in LV cardiomyocytes using TEM (Figure 7E). Representative micrographs showed that in comparison with the SHAM and NAC groups that exhibit ovoid, medium-sized mitochondria with well-preserved cristae, NX cardiomyocytes showed disorganized small spherical mitochondria with fragmented cristae. In contrast, NX + NAC exhibited long, apparently fused mitochondria with partially preserved cristae and an ultrastructural morphology that confirmed the influence of NAC in the mitochondrial structure. Thus, the influence of NAC in mitochondrial protection is associated with its ultrastructure preservation due to a decrease in the ROS production rate.

**Figure 8:**
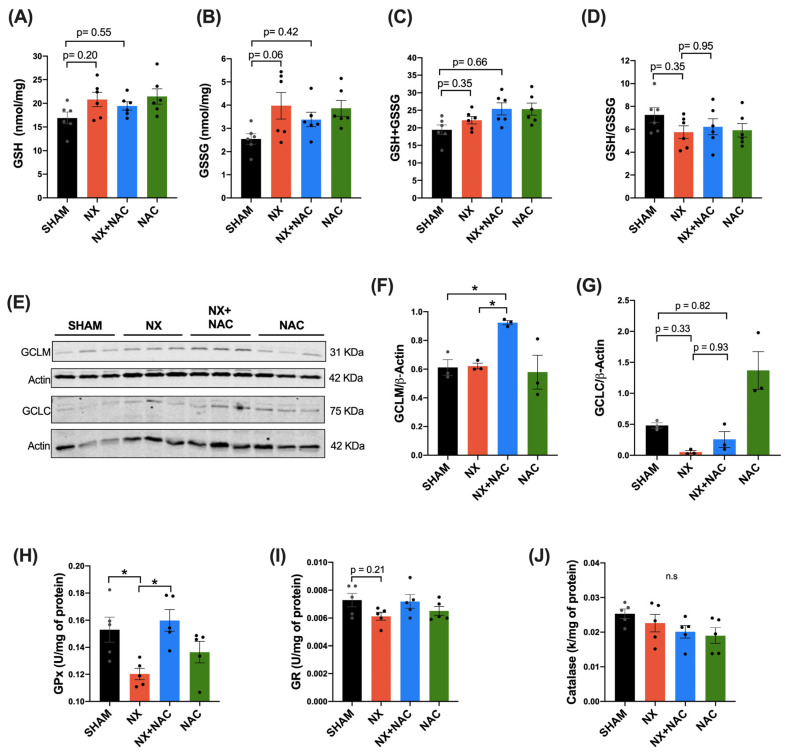
Effects of NAC on the glutathione (GSH) levels, enzymes involved in GSH synthesis, and GSH antioxidant system and homeostasis in the heart during CRS-4 development. (**A**) Glutathione (GSH), (**B**) glutathione disulfide (GSSG), (**C**) total glutathione (GSH + GSSG), (**D**) glutathione ratio (GSH/GSSG), (**E**) immunoblot of the glutamate–cysteine ligase modifier subunit (GCLM), the glutamate cysteine ligase catalytic subunit (GCLC), and (**F**) densitometric analysis of the GCLM and (**G**) GCLC (n = 3). Actin was used as a loading control for total protein. (**H**) Glutathione peroxidase (GPx), (**I**) glutathione reductase (GR), and (**J**) catalase activity in the LV of the heart. Data are mean ± SEM, analyzed by one-way ANOVA followed by Tukey’s test. n = 3–6: individual values are denoted with black dots for each group. * *p* < 0.05, n.s *p* > 0.05. SHAM: simulated surgery + vehicle; NX: 5/6 Nephrectomy + vehicle; NX + NAC: 5/6 Nephrectomy treated with N-acetylcysteine; NAC: control healthy rats treated with N-acetylcysteine.

As the cysteine moiety of NAC is a precursor for GSH synthesis, the latter involved in redox signaling, we evaluated whether the antioxidant properties of NAC were associated with regulating GSH levels in the heart. First, we assessed the GSH, GSSG, total glutathione (GSH + GSSG), and ratio of GSH/GSSG in the total heart homogenate (Figure 8A–D). We found that GSSG levels tended to increase in the NX group, while in the NX + NAC, these levels did not change (Figure 8B). In the other parameters, we did not detect any change (Figure 8A,C,E). Thus, it was further investigated if there were changes in the cytosolic enzymes required for glutathione synthesis. We assessed the protein levels of the subunits of the rate-limiting enzyme glutamate cysteine ligase (GCL), the glutamate–cysteine ligase modifier subunit (GCLM), and the glutamate–cysteine ligase catalytic subunit (GCLC) by WB. Interestingly, the levels of the positive activity modulator GCLM increased significantly only in the NX + NAC group (*p* < 0.05) (Figure 8E,F), suggesting more GCL activity and probably more synthesis of GSH in cardiac tissue. In comparison, the GCLC had a trend to decrease in the NX group compared to in SHAM group (*p* = 0.33), and it showed slight changes in the NX + NAC group compared to in the SHAM group (*p* = 0.82) (Figure 8E,G). On the other hand, the GSH-dependent antioxidant system enzymes GPx and glutathione reductase (GR), which are involved in peroxide detoxification, were also evaluated. It was found that compared to that of the SHAM group, the activity of GPx in the heart of the NX group was significantly decreased (*p* < 0.05). In contrast, in the NX + NAC group, their levels were restored (Figure 8H). In the case of GR, we did not detect changes in the enzyme activity, only a trend to decrease in the NX group (Figure 8I). Catalase activity (which neutralizes H_2_O_2_) also did not change between the groups. These data suggested that the antioxidant effects of NAC could be mediated by GSH regulation.

**Figure 9:**
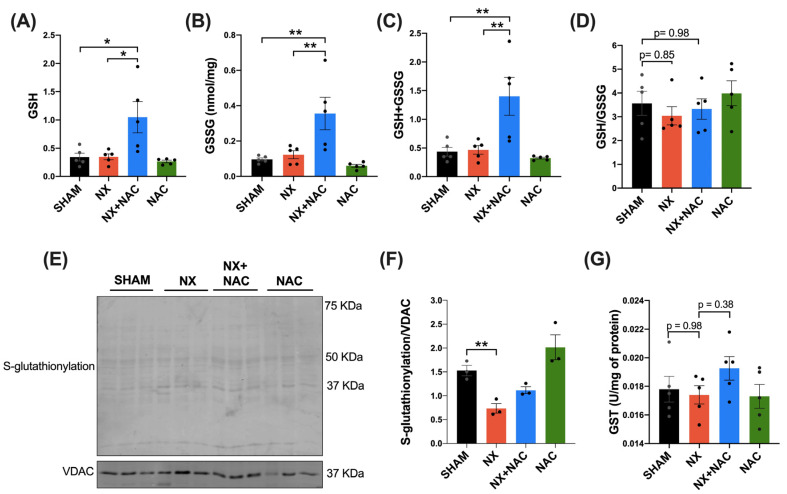
N-acetylcysteine (NAC) increases the mitochondrial glutathione content and S-glutathionylation in the left ventricle mitochondria. (**A**) Glutathione (GSH), (**B**) glutathione disulfide (GSSG), (**C**) total glutathione (GSH + GSSG), and (**D**) glutathione ratio (GSH/GSSG) in the heart’s isolated mitochondria. (**E**) Immunoblot of S-glutathionylated proteins and (**F**) densitometric analysis. The voltage-dependent anion channel (VDAC) was used as a loading control. (**G**) The enzymatic activity of glutathione S-transferase. Data are mean ± SEM, analyzed by one-way ANOVA followed by Tukey’s test. n = 3–5: individual values are denoted with black dots for each group. * *p* < 0.05 and ** *p* < 0.01. SHAM: simulated surgery + vehicle; NX: 5/6 Nephrectomy + vehicle; NX + NAC: 5/6 Nephrectomy treated with N-acetylcysteine; NAC: control healthy rats treated with N-acetylcysteine.

Because no changes were noted in cellular GSH levels but there were changes in GSH-associated enzymes (Figure 8) and mitochondrial function (Figure 5), we hypothesized that the antioxidant effect of NAC could be related to these organelles. Because of this, we measured the changes in the glutathione levels in isolated mitochondria (Figure 9). Remarkably, we found that the levels of GSH, GSSG, and GSH + GSSG were significantly increased in the cardiac-isolated mitochondria of the NX + NAC group, which suggested a selective regulation of NAC in the mitochondrial GSH (Figure 9A–D).

RS-SG mainly occurs in mitochondrial proteins, serving as a protective mechanism for protein oxidation, which depends on the GSH/GSSG rate and H_2_O_2_ levels; therefore, we evaluated the RS-SG levels in these organelles. Interestingly, compared with in the SHAM group, we observed that protein RS-SG was significantly reduced in the NX group but not in the NX + NAC group (Figure 9E,F). Next, we measured the enzymatic activity of GST (an enzyme involved in RS-SG) and observed that GST activity had a trend to increase in the NX + NAC group, but with no changes in the other groups. These results suggested that NAC avoids the excessive oxidation of heart mitochondrial proteins by increasing GSH and restoring RS-SG.

### 3.4. NAC Protected Cardiac Mitochondria by Restoring the Redox Status, Increasing SIRT3 Levels, and Improving the SOD-2 Activity in the LV of the Heart in Animals with CRS-4 Development

The alterations in the redox status are evidenced by a decrease in the NAD^+^/NADH ratio and an increase in the acetyl-CoA/CoA ratio, which produces the acetylation of mitochondrial and cellular lysines in proteins. Therefore, we evaluated the total NAD^+^/NADH, reduced NADH, and NAD^+^ levels in the LV of the hearts of animals with CRS-4 development. As shown in Table 1, the NADH levels significantly increased in the NX group compared to the SHAM group (*p* < 0.05, 192 ± 45 vs. 93 ± 12, respectively). In contrast, in the NX + NAC group, the NADH levels significantly decreased (69 ± 17), close to those in the SHAM group, and the NAD^+^/NADH ratio increased (8 ± 1.4). These changes correlated with ETS impairment in the NX group during CKD because CI decreased activity (Figure 6A), which is responsible for NADH oxidation. These results suggested that NAC maintains the cardiac mitochondrial redox state of the heart during CKD.

**Figure 10:**
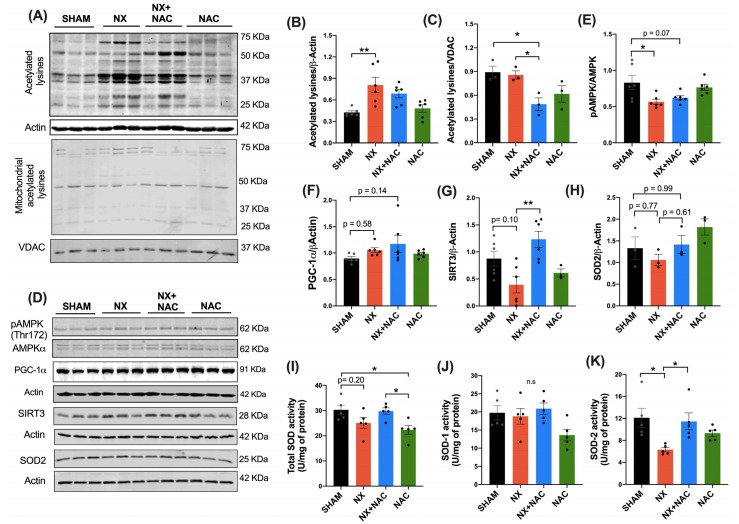
NAC decreased the total and mitochondrial protein lysine acetylation, restored the redox status, increased SIRT3 levels, and improved the SOD-2 activity in the LV of the heart in animals with CRS-4 development. (**A**) Immunoblot and densitometric analysis of (**B**) the protein’s acetylated lysines in the total homogenate of the heart’s left ventricle and (**C**) the protein’s acetylated lysines in isolated mitochondria. (**D**) Immunoblot and densitometric analysis of (**E**) phosphorylated adenosine monophosphate (AMP)-activated protein kinase alpha (pAMPK) and non-phosphorylated AMPKα, (**F**) peroxisome proliferator-activated receptor gamma coactivator 1-alpha (PGC-1α), (**G**) sirtuin 3 (SIRT3), and (**H**) superoxide dismutase- 2 (SOD-2) protein levels in the heart. Actin was used as a loading control for the total protein lysine acetylation and the voltage-dependent anion channel (VDAC) for the mitochondrial protein lysine acetylation (n = 3–6). The determination of the antioxidant activities of (**I**) total SOD, (**J**) SOD-1, and (**K**) SOD-2, n = 5. Data are mean ± SEM, analyzed by one-way ANOVA followed by Tukey’s test. The individual values are shown in black dots for each group. * *p* < 0.05, ** *p* < 0.01, and n.s *p* > 0.05. SHAM: simulated surgery + vehicle; NX: 5/6 Nephrectomy + vehicle; NX + NAC: 5/6 Nephrectomy treated with N-acetylcysteine; NAC: control healthy rats treated with N-acetylcysteine.

The rise in acetyl-CoA produces the acetylation of mitochondrial and cellular lysines depending on the NAD^+^/NADH ratio [50,51]. Thus, we evaluated the lysine acetylation levels in the LV proteins and proteins from LV-isolated mitochondria. The acetylation levels in the total homogenate increased significantly in the group with NX compared to in the SHAM group (*p* < 0.01) (Figure 10A,B). On the contrary, the levels of acetylated lysines decreased in the NX + NAC group, but this change was not significant versus NX. In the cardiac-isolated mitochondria, we observed that the acetylated lysines increased in the NX group compared to in SHAM group (*p* = 0.25), and they decreased in the NX + NAC group (*p* = 0.06) (Figure 10A,C). Thus, changes in the cardiac cellular redox state modify the protein’s acetylation profiles, which NAC treatment decreases during CRS-4 development.

Because we observed that there was a deregulation in the NAD^+^/NADH ratio and an increase in the acetylated lysines in heart proteins during CRS-4 and because recent research has proposed that NAC can modulate mitochondrial redox homeostasis through the adenosine monophosphate (AMP)-activated protein kinase alpha (AMPKα)- peroxisome proliferator-activated receptor gamma coactivator 1-alpha (PGC-1α)-SIRT3-SOD2 pathway in renal and acute cardiorenal disease [21,22,23], we investigated whether this signaling pathway was also modified in the heart’s mitochondria. We found that compared to in the SHAM group, the phosphorylated AMPK (p-AMPK) (Figure 10A,D, *p* < 0.05) and SIRT3 protein levels decreased in the NX group (Figure 10A,G, *p*= 0.10). On the contrary, the NX + NAC group recovered its p-AMPK levels and significantly increased SIRT3 levels (*p* < 0.01). In the following results, we did not observe changes in PGC-1α and SOD-2 protein levels (the main target of SIRT3) in the NX and NX + NAC groups (Figure 10A,F,H). However, because SIRT3 is a deacetylase that regulates the activity of SOD-2 and requires NAD^+^ as a co-substrate [52,53], we evaluated the activity of SOD enzymes (Figure 10I–K). Compared to in the SHAM group, the activity of SOD-2 in the NX group was significantly decreased (*p* < 0.05) (Figure 10K), with no significant changes in the total SOD and SOD-1 (copper/zinc superoxide dismutase) (Figure 10I,J). Interestingly, SOD-2 activity increased in the NX + NAC group (*p* < 0.05), suggesting that NAC could improve the enzymatic activity of SOD-2 because it is the main target of SIRT3 for lysine deacetylation. Therefore, it would be essential to evaluate this signaling pathway in the heart during CRS-4 development with genetic tools to determine whether NAC exerts its antioxidant regulation through specific signaling on the SIRT3 deacetylase.

## 4. Discussion

In this study, we used the NX model to evaluate the effect of NAC on CKD-induced cardiovascular damage, mitochondrial alterations, redox, and SIRT3 disturbances. In this model, systolic blood pressure was increased and CKD was established within 4 to 8 weeks [24,25,26]. Compared with this, we demonstrated that renal damage markers (BUN and creatinine in plasma) were increased in the NX animals from 2 weeks post-surgery (Supplementary Figure S1). The oral treatment with NAC did not prevent CKD disturbances at 8 weeks (Figure 2). However, NAC during CRS-4 development improved systolic blood pressure and cardiac fibrosis. The protective effect was associated with preserving the mitochondrial bioenergetics and the redox state in cardiac mitochondria. In addition, NAC increased the SIRT3 levels and decreased the mitochondrial protein lysine acetylation, indicating that SIRT3 probably induced protein regulation and increased the activity of its main target SOD-2.

The kidney is essential for energy metabolism, detoxification, and the excretion of nitrogenous waste products. CKD promotes cardiac ROS overproduction and oxidative stress, leading to alterations in cardiomyocyte excitation–contraction coupling, calcium regulation, and changes in energy metabolism [54,55,56]. Accordingly, we observed a notable increase in SBP and a decrease in HR in the NX group that were improved in the NX + NAC group (Figure 2D and Figure 3H). Increased blood pressure is generally associated with increased HR [57,58]. Despite this relationship, controversial observations have been observed in CKD, where renal dysfunction, fluid retention, and plasma volume disturbances increase blood pressure but decrease HR variability. These alterations have been associated with peripheral nerve alterations due to uremia, which causes sympathetic and parasympathetic systems dysfunction, known as uremic neuropathy [54,55,56]. The reduction in SBP in the NX + NAC rats and the maintenance of HR could be attributed to the anti-inflammatory effects and hemodynamic regulation conferred by NAC [50,51]. According to this, studies in hypertensive humans and animals have demonstrated that NAC lowers blood pressure by increasing GSH levels, decreasing oxidative stress, and modulating nitric oxide levels and other vasoactive molecules [59,60]. In addition, the endothelial function and markers of cardiac damage, such as B-natriuretic peptide, were decreased in patients with CRS-2 treated with NAC (500 mg orally twice for 28 days), suggesting that NAC is a potent antioxidant that preserves hemodynamics and blood pressure [60]. We cannot separate the antioxidant effects of NAC on renal function and the antihypertensive effect from its direct impact on cardiac cells. However, it has been suggested that NAC’s primary mechanism of action is to penetrate cells, where it is hydrolyzed to cysteine by acylase enzymes [61]. Free cysteine is an intracellular limited precursor for GSH synthesis. Most plasma cysteine exists in its oxidized form, cystine, which sustains glutathione synthesis at an acceptable level (1.0 mM). NAC provides cysteine through intracellular deacetylation in plasma-free solutions, but the concentrations necessary to do so are too high (more than 1.0 mM). Thus, the mechanism of action of cysteine is to reduce plasma cystine to cysteine, which then enters cells for GSH synthesis [61]. The determination of cysteine and its derivatives in the plasma of rats could be measured in additional studies with ultra-high performance hydrophilic interaction liquid chromatography [62].

ROS overproduction promotes inflammation and fibrosis, contributing to the restructuring of the extracellular matrix and exacerbating both systolic and diastolic dysfunctions [63,64]. We observed increased cardiac fibrosis and inflammation measured by IL-1β protein levels in the NX animals. NAC treatment did not completely decrease these alterations (Figure 4B,E). Heart inflammation is an active participant in the early phases of CKD [65], persisting through its chronic stages [25]. The kidney’s production and release of proinflammatory mediators reduce compliance and increase cardiac stiffness, impairing heart function and altering the heart’s electrical activity [66]. Proinflammatory factors such as IL-1β trigger cardiomyocyte apoptosis and oxidative stress [67,68]. In addition, in CRS type 3, IL-1β and IL-6 levels increased in plasma, establishing a possible cardiorenal connector [21]. In cardiac pathology, IL-1β regulates the inflammatory response and induces cardiomyocyte hypertrophy by regulating atrial natriuretic factors, suppressing calcium-regulating genes [69], and leading to vasoconstriction of the arteries [70]. An important consideration of our study is that we did not evaluate other proinflammatory cytokines such as IL-6. Therefore, this would be important in future studies involving the cardiorenal evaluation.

In the heart, the highly active contractile work determines the ATP and oxygen consumption rates [71]; 80 to 90% of ATP is produced from OXPHOS. Therefore, OXPHOS failure leads to contractile dysfunction and heart failure. In addition, mitochondrial OXPHOS capacity decreases, and coupling loss is associated with lower Δψm. Our results showed that OXPHOS heart impairment in CKD may be related to heart CI, CII, and CIV decreasing in activity in the NX group (Figure 5 and Figure 6A–C). These changes were partially explained by the partial diminution in the ETS subunit contents, especially CI and CII (Figure 6D–I), suggesting a decrease in mitochondrial mass that can explain the respiratory parameter alterations. Although we did not measure Δψm in this study, we previously reported that CKD induces a permanent decline in renal Δψm, which is accompanied by a higher ROS production [18]. In this way, cardiac mitochondrial respiration changes and decoupling (Figure 5B–D) can be associated with an increase in mitochondrial H_2_O_2_ production rates (Figure 7B–D) and ultrastructure alterations (Figure 7E). Together, these results suggested that CKD also induced the heart loss of Δψm. The loss of Δψm causes issues in mitochondrial respiration, oxygen consumption, and substrate utilization, leading to changes in myocardial energetics and endothelial function [14]. NAC prevented these cardiac mitochondrial bioenergetic changes in all respiratory states (S3, S4o, P, and RCI) and decreased the mitochondrial H_2_O_2_ production rate (S3).

GSH is an antioxidant metabolite synthesized in the cytosol that prevents irreversible protein oxidation and indicates cellular redox status [72]. NAC increases intracellular GSH levels and counteracts oxidative stress, particularly during CKD [73]. Other studies have shown that NAC prevents GSH depletion in vascular cells and reduces cardiovascular risks in CKD patients by balancing systemic GSH levels [49]. Moreover, NAC normalizes GSSG levels in experimental cardiomyopathy and prevents renal dysfunction from cardiac damage [28]. In this study, we found that the NX group showed increased GSSG levels with the concomitant GSH/GSSG decrease (Figure 8A–D). As GSH biosynthesis depends on factors such as the proper availability of cysteine, the GCL activity, subunit (GCLC and GCLM) levels, and the GSH synthetase [74], we measured the protein levels of the GCLC and GCLM. We observed notable GCLM changes in the NX + NAC group but not the GCLC (Figure 8E–G), which can depend on the differential regulation of the holoenzyme (GCLC + GCLM) modified in response to cysteine deprivation [75]. In addition, GCLC feedback inhibition is regulated by GSH. GCLM upregulation increases GCL activity depending on GSH levels [76]. Thus, changes in the GCLM protein levels, but not the GCLC ones, in the NX + NAC group suggested a differential regulation in response to cysteine availability, which improved GSH synthesis. Moreover, a significant decrease in the GPx activity was observed in the NX group compared to in the NX + NAC group, thus suggesting that GSH oxidation increases in the heart due to chronic oral NAC administration. This modification may also be related to the fact that the levels of H_2_O_2_ production decreased in the heart’s mitochondria during the development of CRS-4.

Mitochondria harbor 10–15% of the total cellular GSH but lack the enzymes to synthesize it. However, mitochondrial GSH originates from cytosolic GSH via transport through specific mitochondrial carriers, through both mitochondrial membranes, and depending on the sensitivity to membrane dynamics [77]. In our results, we showed that GSH, GSSG, and GSH-GSSG levels increased in the isolated mitochondria of the NX + NAC group (Figure 9). Thus, we can hypothesize that NAC promoted an increased synthesis of GSH in the cytosol through increasing cysteine levels. Then, it could be transported to cardiac mitochondria by the triggered expression of mitochondrial GSH transporters such as the solute carrier family 25 member 39 (SLC25A39), which shows a feedback regulatory mechanism depending on mitochondrial GSH levels [78]. However, we did not evaluate these transporters because it was beyond our primary objective. Thus, it would be interesting to assess whether GSH transporters are specifically modulated functionally or transcriptionally in cardiac cells and identify which specific cells are more susceptible to GSH alterations during CRS-4.

The catabolism of fats and carbohydrates provides the electrons to reduce NAD^+^ to NADH [79]. In the presence of oxygen, NADH is oxidized by the ETS, where the oxygen in CIV is the terminal electron acceptor. During electron transfer, the proton motive force that develops across the inner mitochondrial membrane drives ATP synthesis [80]. Thus, NADH must be oxidized in the ETS and converted into NAD^+^ in normal conditions. NAD^+^ regulates intermediary metabolism and is an essential substrate for the deacetylase activity of sirtuins, which are important regulators of the redox state, antioxidant signaling, and metabolism sensors [81]. Thus, we explored the effect of NAC on mitochondrial redox signaling by measuring the NADH and NAD^+^ levels in the LV of the heart. The levels of NADH were significantly increased in the NX group compared to in the SHAM group (Table 1). This agrees with the trend in the reduction in CI activity (Figure 6A), suggesting a change in the cardiac mitochondrial redox and bioenergetic state during CRS-4. On the contrary, in the NX + NAC group, NADH levels significantly decreased, NAD^+^/NADH levels increased (Table 1), and CIV activity was recovered (Figure 6A). A decrease in the activity of CI, which oxidizes NADH and reestablishes the levels of NAD^+^, can produce an increase in the Krebs cycle because dehydrogenase enzymes also produce NADH. Still, some of them, such as isocitrate dehydrogenase 3, are regulated allosterically by negative effectors such as ATP, NADH, and NADPH [82]. Therefore, the increase in NADH levels by the reduction in CI and a very active TCA cycle affects the redox state in cells. In addition, the metabolic reprogramming of the failing heart is characterized by transcriptional and post-transcriptional modifications associated with the downregulation of enzymes in myocyte fatty acid transport and oxidation, the main pathway to produce ATP and NADH in cardiomyocytes [83]. A significant limitation of our study is that we did not evaluate enzymes of the Krebs cycle, fatty acid oxidation, and ATP levels to determine these metabolic modifications. However, these are perspectives that could be considered for future studies because tissue metabolic profiles and the identification of metabolic signatures could give more information about the energetic state in the heart during the progress of CKD and look for therapies that can effectively modulate these modifications.

Acetyl-CoA produces the acetylation of mitochondrial and cellular lysines depending on the NAD^+^/NADH ratio [50,51]. In this study, we observed NAD^+^/NADH ratio alterations. Therefore, we evaluated the lysine acetylation levels in the LV proteins and proteins from LV-isolated mitochondria. Protein lysine acetylation levels in the heart homogenate increased in the group with NX compared to in the SHAM group (Figure 10A,B). On the contrary, the levels of acetylated lysines decreased in the NX + NAC group, but this change was not significant. In cardiac-isolated mitochondria, we observed that the protein’s acetylated lysines increased in the NX group compared to in the SHAM group and decreased in the NX + NAC group (Figure 10A,C), suggesting that NAC regulates the mitochondrial homeostasis in the heart, preventing the acetylation of mitochondrial proteins. Reversible deacetylation of several mitochondrial proteins by sirtuins is an essential mechanism for the control of energy metabolism [84], and the role of these modifications during CKD has been demonstrated in studies with GSH precursors like NAC [18,22]. In addition, the AMPK-SIRT3-PGC-1α axis coordinates mitochondrial homeostasis at the renal and cardiac levels [85,86], as well as enhances TFAM expression, mitochondrial CI and CII, and mitochondrial DNA replication [86,87].

PGC-1α induces the gene expression of different components of the ETS through SIRT3 and the expression of ROS-detoxifying enzymes [86,87]. Thus, this axis may upregulate antioxidant enzymes and decrease caspase 3 activity, enhancing cardiomyocyte viability [52,88]. In addition, SIRT3 is a deacetylase that modifies and controls several mitochondrial proteins and requires NAD^+^ as its substrate [53]. Thus, we evaluated the AMPK-PGC-1α-SIRT3-SOD2 axis in our model and the effect of NAC (Figure 10D–H). Interestingly, in the NX + NAC group versus the NX group, the AMPK and SIRT3 protein levels were preserved, but the PGC-1α and SOD-2 protein levels did not change (Figure 10D,F,H). However, SOD-2 enzymatic activity was significantly increased (Figure 10K). This suggested that the increase in SIRT3 and its cofactor NAD^+^ could promote the deacetylation of this antioxidant enzyme. In line with these observations, NAC administration during diabetic nephropathy activated mitochondrial SIRT3 and prevented the hyperacetylation of SOD-2. The modulation of these mechanisms was attributed to NAC’s ability to regulate GSH levels through Gpx4, thus maintaining mitochondrial redox homeostasis [22]. In a study by Peerapanyasut et al. [23], NAC treatment diminished bisphenol A-induced kidney and liver functional alterations by reducing oxidative stress, enhancing AMPK-PGC-1α-SIRT3 signaling, and decreasing SOD-2 hyperacetylation. However, a critical limitation of our findings is that we did not evaluate the acetylated form of SOD-2, which must be considered in the following studies.

Although the antioxidant NAC has been widely used, its chronic effects as a treatment have not been fully elucidated. This may be since opposite effects have been observed in different pathologies. For example, Small et al. [89] showed that in the progression of acute kidney disease to CKD induced by ischemia–reperfusion (I/R) in mice, the pre-administration of NAC (5% seven days before I/R) enhanced metabolic impairment and mitochondrial dysfunction by promoting the dysregulation of antioxidant enzymes [89]. In addition, adverse oral and intravenous effects due to incorrect dosing have been reported. Those include anaphylaxis, rash, pruritus, and hypotension [90]. Thus, the administration time, correct solution preparation, pH adjustment, and route of administration are essential when NAC is used as a treatment because its pharmacodynamics, pharmacokinetics, and half-life highly depend on these factors. For example, using non-pH-adjusted NAC solutions induces the acidification of the medium and modifies experimental outcomes [91]. Moreover, single intravenous doses from 500 mg/kg to 2445 mg/kg in several species have been shown to be lethal [92]. However, other studies showed that the correct and continuous use of NAC reduces the risk of CKD [93]. For example, in clinical settings, an intravenous dose of 150 mg/kg NAC improved endothelial function and increased homocysteine removal in hemodialyzed patients [49]. In patients with CKD in stage five, the intravenous administration of 5000 mg of NAC during the hemodialysis session improved vascular reactivity pressure in reactive hyperemia [94].

An important consideration of our research is that we administered NAC as a treatment. Compared with other studies, NAC has shown to be functional if given as a pre-treatment in CKD models. However, it has been demonstrated that the treatment with NAC 7 days after 5/6 NX improves renal function [29] and attenuates systemic oxidative stress in uremic rats [73]. Therefore, it would be essential to evaluate whether administration one week earlier in our model could generate a more significant benefit. Because of that, we probably did not see substantial changes in renal function. However, improvements were noted in systolic blood pressure, cardiac fibrosis, mitochondrial function, redox status and SIRT3 levels. This adds interesting new data to the field of cardiorenal diseases since we established that administering antioxidants (such as NAC) during established CKD can promote benefits to mitochondrial function and that modifying the redox state is vital to reduce alterations in the heart. However, in this study, we could not observe a fully therapeutic effect on the kidney or the heart since the different pathophysiological mechanisms in CKD likely remain active (e.g., activation of the renin–angiotensin–aldosterone system, uremia, dyslipidemia, proteinuria, fibrosis and secretion of growth factors and cytokines, among others). In addition, the 5/6 NX model produces sustained and progressive hypertension that is closely related to cardiovascular damage, producing fibrosis, cardiac remodeling, and ultimately cardiac dysfunction [95], which are related to other signaling pathways independent of NAC effects. In this regard, NAC can be proposed as an adjuvant treatment that could be administered with currently used drugs to treat CKD such as antihypertensives, angiotensin-converting enzyme inhibitors, angiotensin receptor blockers, sodium-glucose cotransporter inhibitors, and statins [96].

The effects of the oral administration of NAC as a potential strategy to treat mitochondrial and redox signaling disturbances on CRS-4 have not been explored until now. Thus, the current study presents novel insights, suggesting that long-term treatment with NAC significantly increases mitochondrial GSH levels, enhances mitochondrial function and redox signaling, and promotes an increase in SIRT3 while reducing cardiac damage during CRS-4 development in a clinically relevant model. Consequently, NAC emerges as a potential strategy to fight the detrimental mitochondrial alterations associated with this disease.

## 5. Conclusions

This study uncovers that NAC treatment during CKD significantly improved mitochondrial health by increasing GSH levels within these essential organelles while effectively modulating the redox state and the redox-sensitive cysteine residues. This intervention not only modulated the redox state through positively affecting redox-sensitive cysteine residues. These improvements were linked to reduced mitochondrial lysine acetylation, an increased NAD^+^/NADH ratio, and recovered SIRT3 levels in the heart during CRS-4. Therefore, the multifaceted intervention with NAC during CRS-4 protects mitochondria from damage and fine-tunes redox signaling dynamics. This study suggests a novel potential approach for delaying the onset of CRS-4.

## Figures and Tables

**Table 1:** NADH/NAD^+^ levels in the left ventricle of the hearts of animals with CRS-4 development.

NAD^+^/NADH Levels (pmol/mg of Protein)	SHAM	NX	NX + NAC
NAD^+^ + NADH	752 ± 65	666 ± 68	620 ± 94
NADH	93 ± 12	192 ± 45 *	69 ± 17
NAD^+^/NADH	7 ± 0.2	3 ± 1.2 *	8 ± 1.4

(NAD^+^/NADH oxidized and reduced forms of nicotinamide adenine dinucleotide). Data are mean ± SEM, analyzed by one-way ANOVA followed by Tukey’s test. N = 5, * *p* < 0.05 vs. NX + NAC. SHAM: simulated surgery + vehicle; NX: 5/6 Nephrectomy + vehicle; NX + NAC: 5/6 Nephrectomy treated with N-acetylcysteine.

## Data Availability

The data supporting this study’s findings are available from the corresponding author (J. Pedraza-Chaverri) upon reasonable request.

## Supplementary Materials

The following supporting information can be downloaded at https://www.mdpi.com/article/10.3390/antiox14030367/s1, Supplementary File S1—Supplementary Figure S1: Renal damage markers (24 h and 15 days) and representative micrographs of renal damage; Supplementary File S2: Original WB membranes; Supplementary File S3: Numerical data; Supplementary Tables: Table S1: List of primary antibodies used for immunoblots; Table S2: List of secondary antibodies.

## Abbreviations

The following abbreviations are used in this manuscript:

Δψm	Mitochondrial membrane potential
2-VP	2-vinylpyridine
ADP	Adenosine 5′-diphosphate
ANOVA	Analysis of variance
ATP	Adenosine 5′-triphosphate
BPM	Beats per minute
BSA	Bovine serum albumin
BUN	Blood urea nitrogen
CCCP	Carbonyl cyanide m-chlorophenylhydrazone
CDNB	1-Chloro-2,4-dinitrobenzene
CI	Complex I
CII	Complex II
CIII	Complex III
CIV	Complex IV
CI-NDUFB8	Reduced form of nicotinamide adenine dinucleotide ubiquinone oxidoreductase subunit B8
CII-SDHB	Succinate dehydrogenase B
CIII-UQCRC2	Ubiquinol–cytochrome c reductase core protein 2
CIV-MTCO1	Cytochrome c oxidase subunit I
CV-ATP5A	Adenine triphosphate synthase-α subunit
CKD	Chronic kidney disease
CoA	Coenzyme A
CRS-4	Cardiorenal syndrome type 4
DCPIP	2,6-dichlorophenolindophenol sodium salt hydrate
DTNB	5,5′-dithio-bis-(2-nitrobenzoic acid)
DTT	Dithiothreitol
DUB	Decyl ubiquinone
DUBH2	Decyl ubiquinol
Echo	Echocardiography
EDTA	Ethylenediaminetetraacetic acid
EF	Ejection fraction
EGTA	Ethylene glycol-bis (2-aminoethyl ether)-N,N,N′,N′-tetraacetic acid
ETS	Electron transport system
G6PDH	Glucose-6-phosphate dehydrogenase
GAPDH	Glyceraldehyde 3-phosphate dehydrogenase
GCL	Glutamate cysteine ligase
GCLC	Glutamate–cysteine ligase catalytic subunit
GCLM	Glutamate–cysteine ligase modifier subunit
GPx	Glutathione peroxidase
GR	Glutathione reductase
GSH	Glutathione
GSH/GSSG	Glutathione ratio
GSH + GSSG	Total glutathione
GSSG	Oxidized glutathione
GST	Glutathione S-transferase
H&E	Hematoxylin and eosin
H_2_O_2_	Hydrogen peroxide
HEPES	4-(2-hydroxyethyl)-1-piperazineethanesulfonic acid
HR	Heart rate
HRP	Horseradish peroxidase
IL-1β	Interleukin-1β
IVS	Interventricular septum
KCN	Potassium cyanide
KH_2_PO_4_	Potassium Phosphate monobasic
KOH	Potassium hydroxide
LDH	Lactate dehydrogenase
LV	Left ventricle
LVDd	Left ventricle dimensions at end-diastole
LVDs	Left ventricle dimensions at end-systole
LVPW	Left ventricular posterior wall
MgCl_2_	Manganese (II) chloride
Na_2_HPO_4_	Sodium phosphate dibasic
Na_3_VO_4_	Sodium orthovanadate
NAC	N-acetylcysteine
NaCl	Sodium chloride
NAD^+^	β-Nicotinamide adenine dinucleotide oxidized
NADH	β-Nicotinamide adenine dinucleotide hydrogen
NADP^+^	β-Nicotinamide adenine dinucleotide phosphate oxidized
NADPH	β-Nicotinamide adenine dinucleotide phosphate reduced
NaF	Sodium fluoride
NaH_2_PO_4_	Sodium phosphate monobasic
NaHCO_3_	Sodium bicarbonate
NaOH	Sodium hydroxide
NaN_3_	Sodium azide
NBT	Nitro blue tetrazolium
NEM	N-ethyl maleimide
NH_4_Cl	Ammonium chloride
Nrf2	Nuclear factor erythroid 2-related factor 2
NX	5/6 Nephrectomy
NX + NAC	5/6 Nephrectomy treated with NAC
O_2_	Oxygen
P	Oxidative phosphorylation-associated respiration
PAGE	SDS–polyacrylamide gel electrophoresis
p-AMPK	Phosphorylated-adenosine monophosphate-activated protein kinase
PEP	Phosphoenolpyruvate
PGC-1α	Peroxisome proliferator-activated receptor-gamma coactivator-1 alpha
PMSF	Phenylmethanesulfonyl fluoride
PVDF	Polyvinylidene fluoride
PW	Posterior wall
PYK	Pyruvate kinase
RCI	Respirometry control index
RIPA	Radioimmunoprecipitation buffer
ROX	Residual respiration
RS-	Thiolate anions
RS-SG	Protein-S-glutathionylation
S3	State 3
S4o	State 4 induced by oligomycin
SBP	Systolic blood pressure
SDHB	Succinate dehydrogenase B
SDS	Sodium dodecyl sulfate
Sham	Simulated surgery
SIRT3	Sirtuin 3
SOD	Superoxide dismutase
SOD-2	Superoxide dismutase-2
TBS-T	Tris-buffered saline with 0.1% Tween 20 detergent
TFAM	Transcription factor associated mitochondria
TMPD	Tetramethyl-p-phenylenediamine
TNB	5-thio-2-nitrobenzoic acid
TnT	Troponin T
Tris	Trizma
Tris-HCl	Trizma-hydrochloride
UQCRC2	Ubiquinol–cytochrome c reductase core protein 2
VDAC	Voltage-dependent anion channel
WB	Western blot
