# Supplementary material for: Effect of N-Acetylcysteine in Mitochondrial Function, Redox Signaling, and Sirtuin 3 Levels in the Heart During Cardiorenal Syndrome Type 4 Development
Source: Antioxidants (Basel). 2025 Mar 20;14(3):367. doi: 10.3390/antiox14030367 (PMC11939543; doi:10.3390/antiox14030367)
Supplement: Supplementary file 1 [file antioxidants-14-00367-s001.zip › Supplementary File S1-Supplementary Figure S1.pdf]

Supplementary Figures

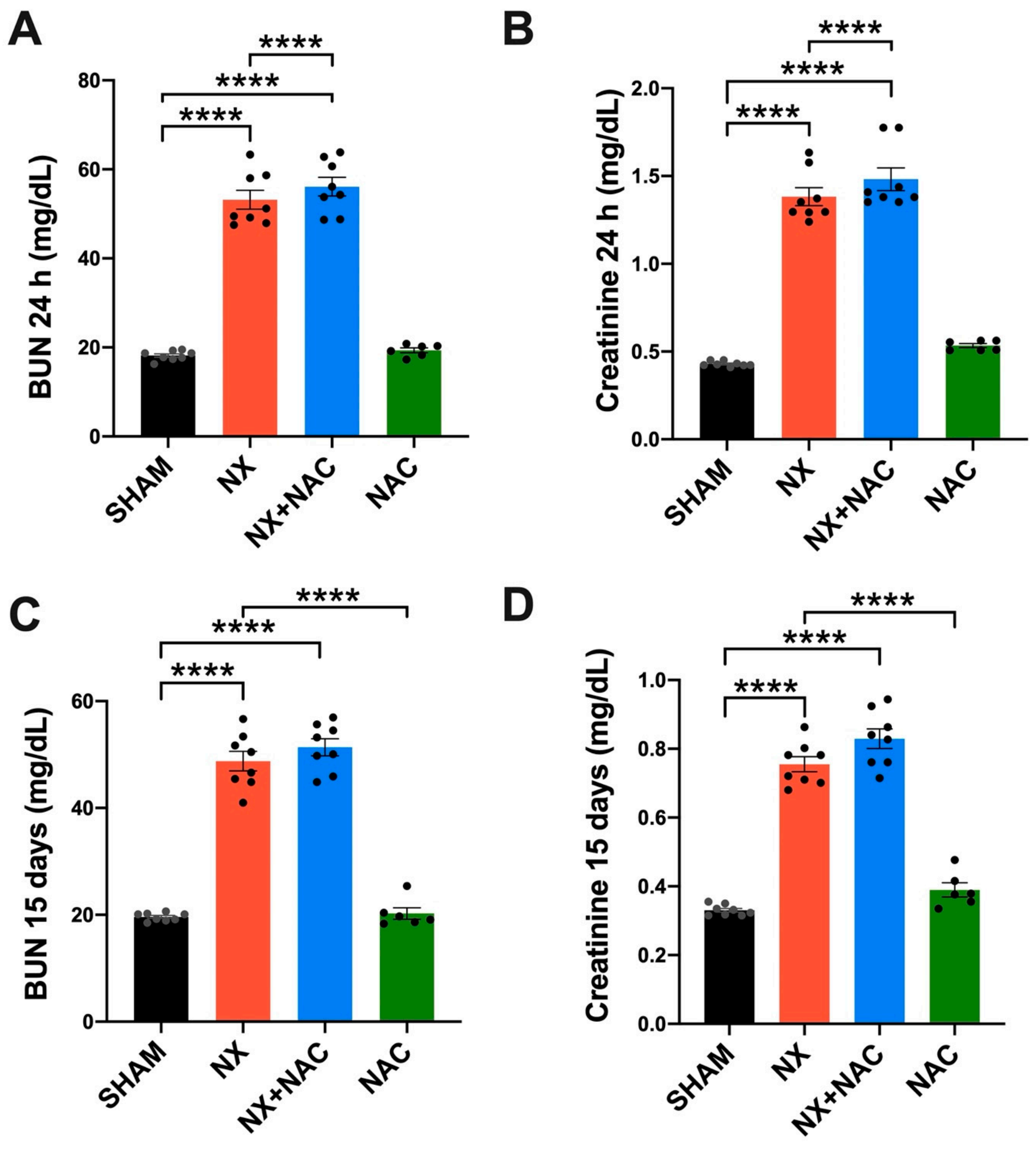

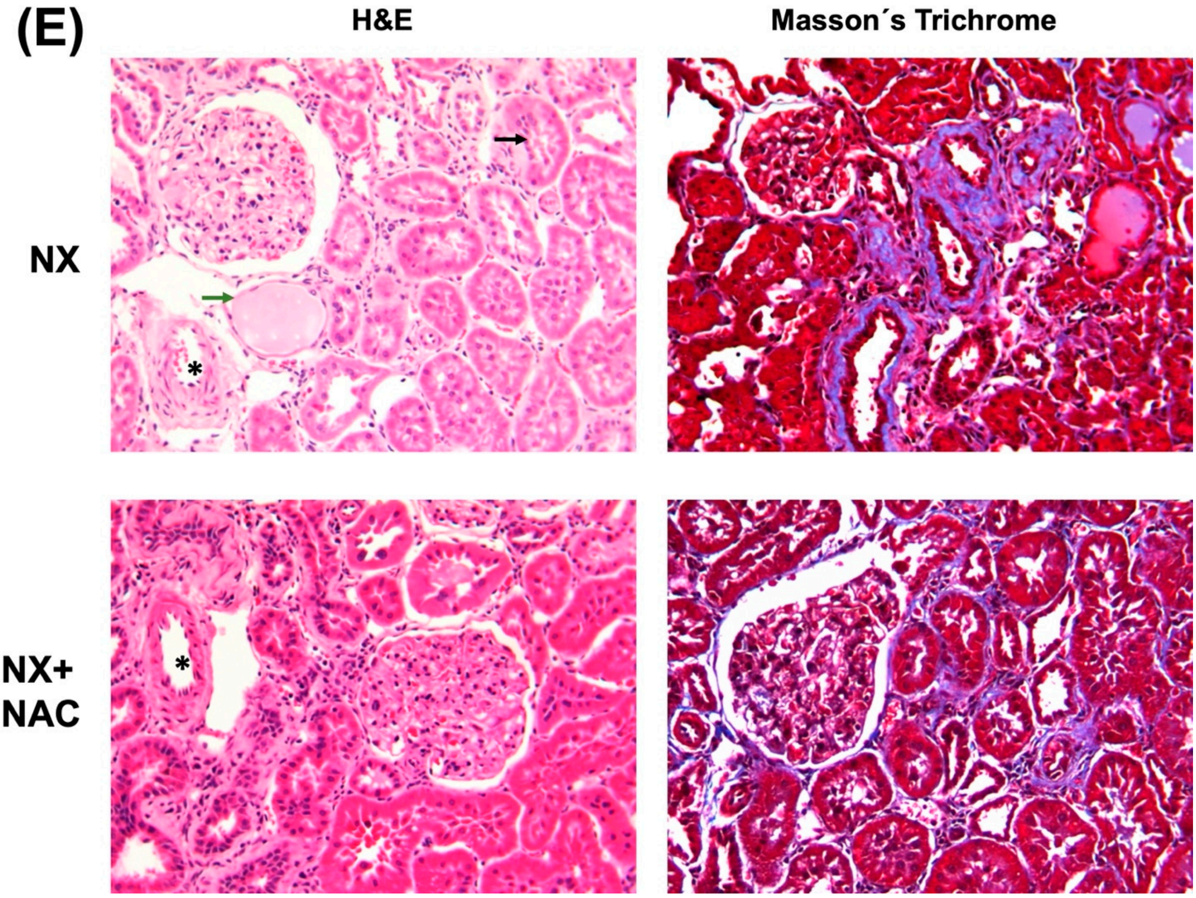

Supplementary Figure S1. Validation of the animal model for studying cardiorenal syndrome type 4. A) BUN and B) creatinine measurements at 24 h after 5/6 nephrectomy (NX). C) BUN, and D) creatinine measurements 15 days after NX. Data are mean±SEM, analyzed by one-way ANOVA followed by Tukey´s test. n=6-8, individual values are denoted with black dots for each group. ***p<0.001, ****p<0.0001. E) Representative micrographs of kidney sections H/E staining from NX rat, showing proximal convoluted tubules with necrotic epithelial cells detachment (blue arrow), tubular atrophy manifested by flattened epithelium and hyaline casts (green arrow), the interstitium shows chronic inflammation and fibrosis, as well as muscular arteries with wide hyperplastic muscular layer (asterisk), the histological abnormalities particularly fibrosis are evidenced by Masson trichrome staining. Kidney section from NX+NAC rat, H/E staining shows lesser tubular damage and fibrosis, muscular artery with slight media hyperplasia (asterisk); Masson trichrome staining evidenced lesser interstitial fibrosis. SHAM: simulated surgery;

NX: 5/6 nephrectomy; NX+NAC: 5/6 nephrectomy treated with N-acetylcysteine; NAC: rats treated with N-acetylcysteine.
