# Supplementary material for: Effect of N-Acetylcysteine in Mitochondrial Function, Redox Signaling, and Sirtuin 3 Levels in the Heart During Cardiorenal Syndrome Type 4 Development
Source: Antioxidants (Basel). 2025 Mar 20;14(3):367. doi: 10.3390/antiox14030367 (PMC11939543; doi:10.3390/antiox14030367)
Supplement: Supplementary file 1 [file antioxidants-14-00367-s001.zip › Supplementary File S2_Original western blot membranes pdf.pdf]

Effects of N-acetylcysteine in mitochondrial function, redox signaling, and Sirtuin 3 levels in the heart during Cardiorenal syndrome type 4 development

Western blot membranes

IL1beta

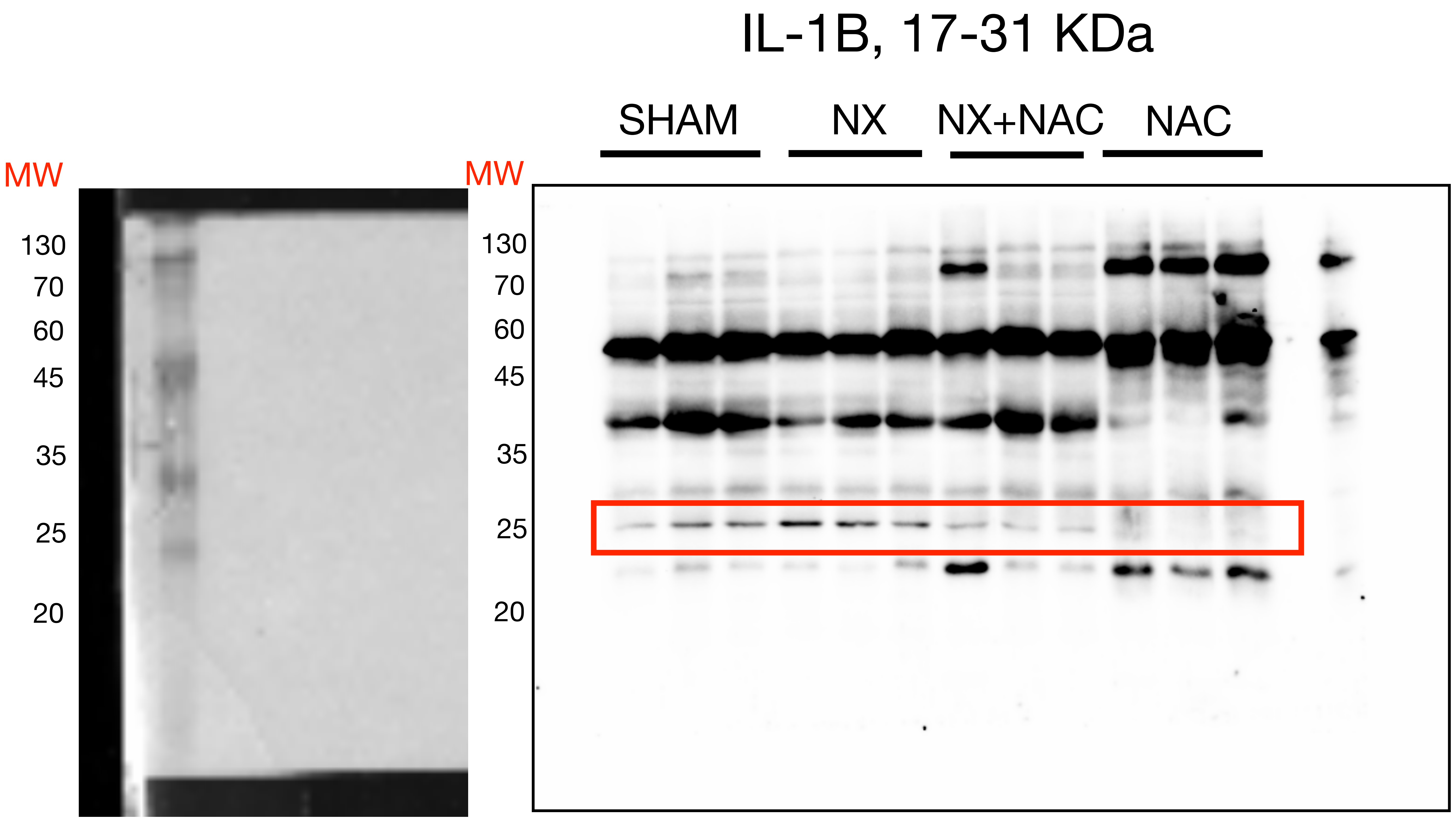

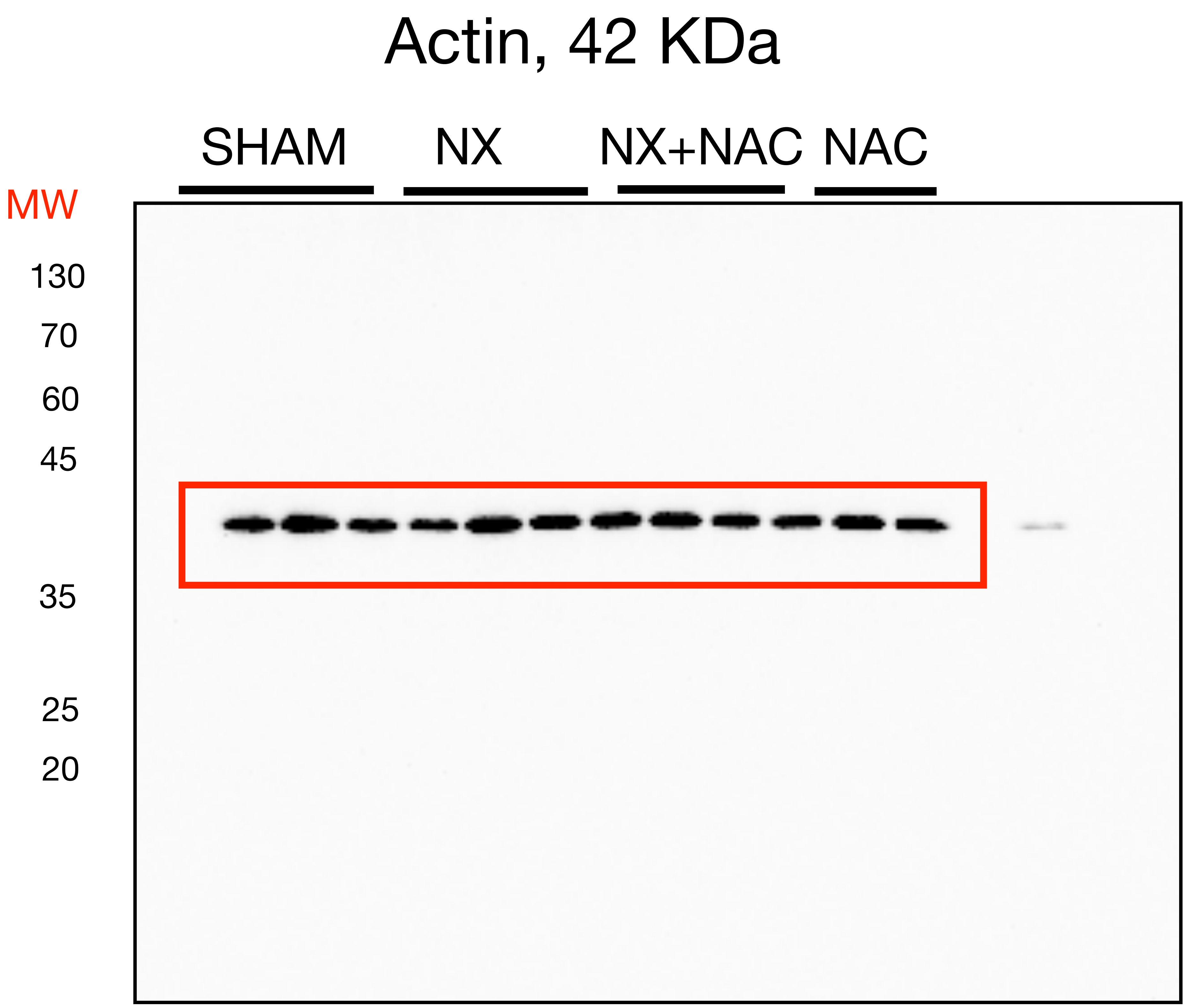

Chemiluminescence

Troponin (plasma)

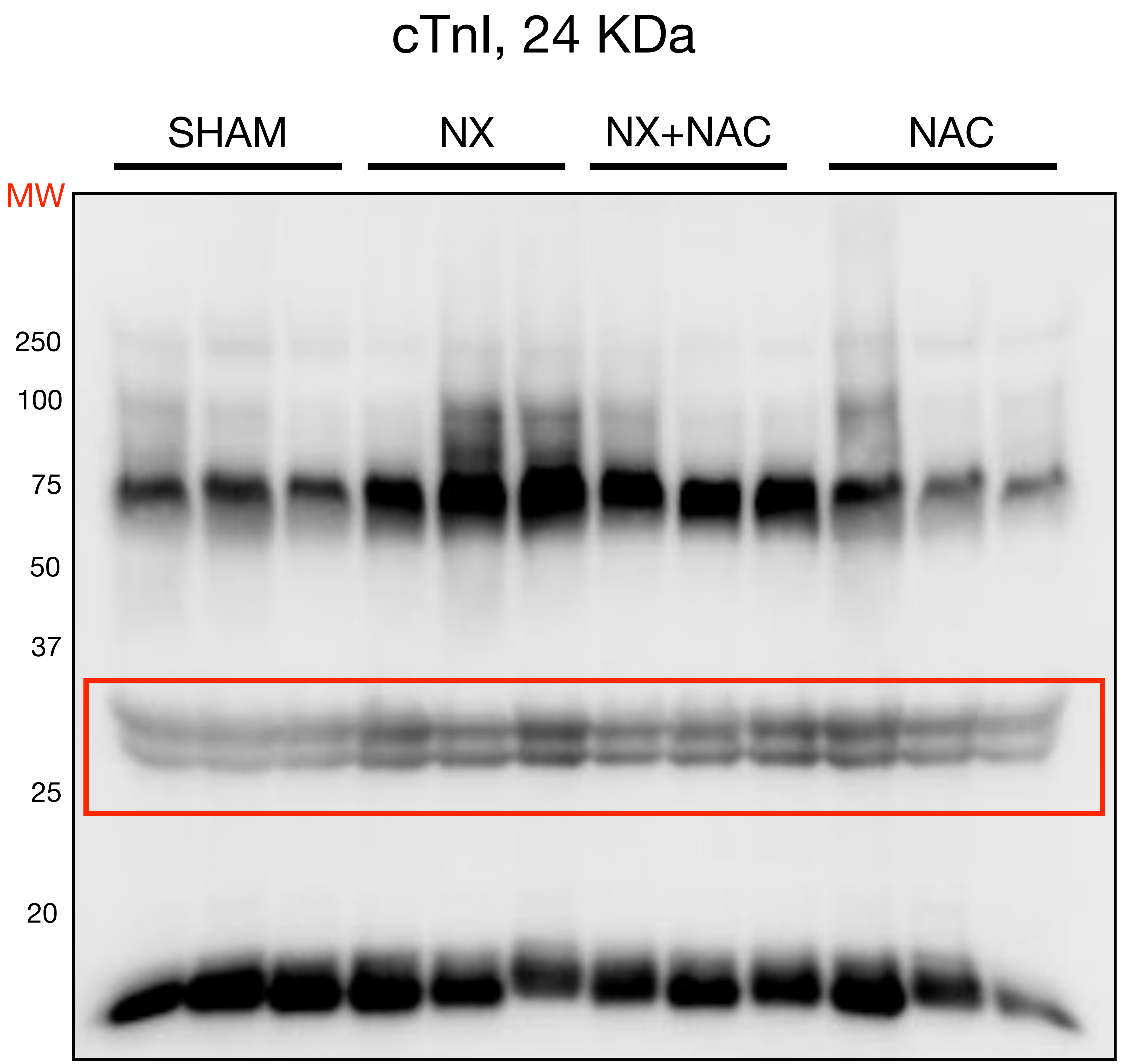

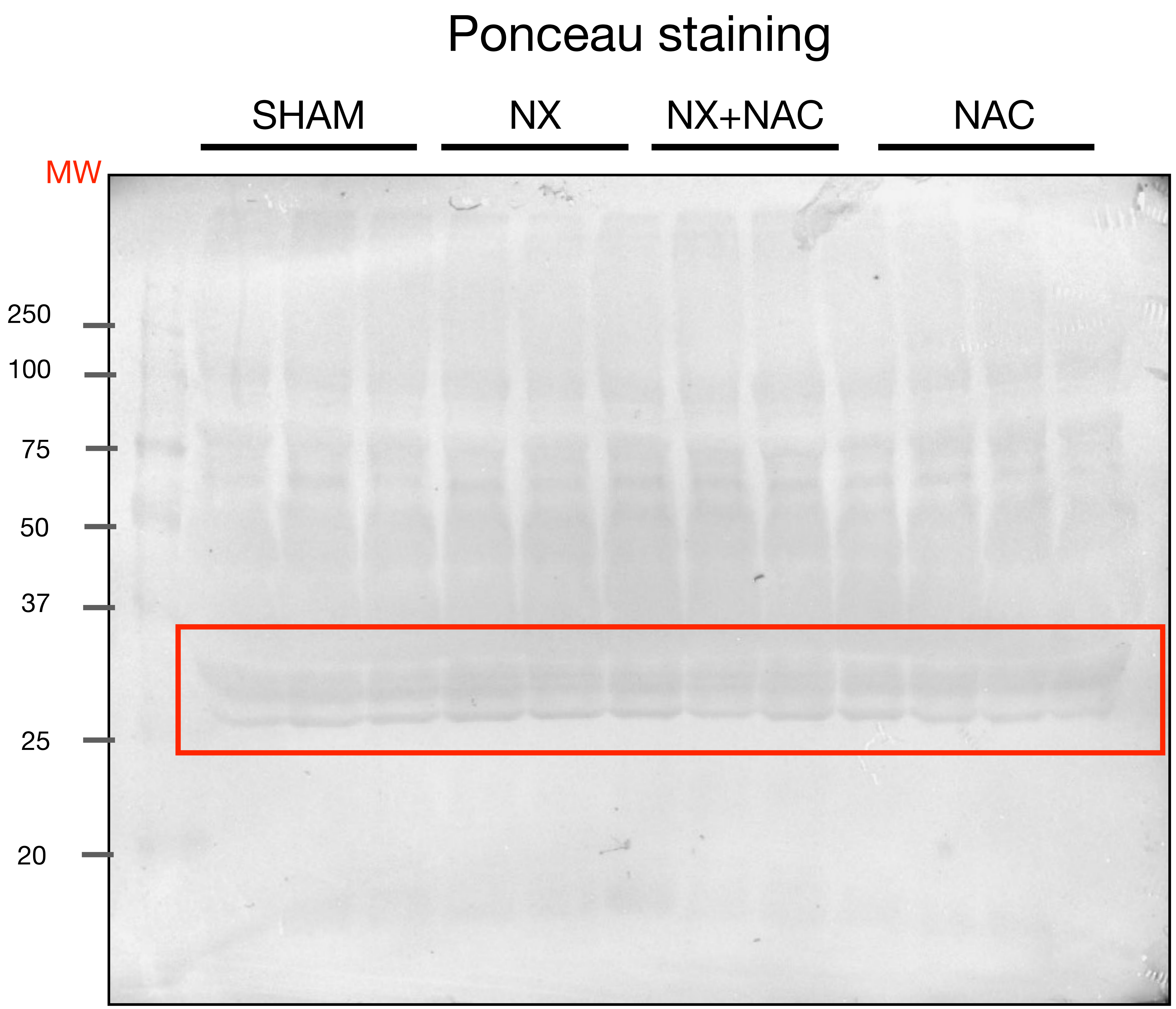

Troponin (plasma)

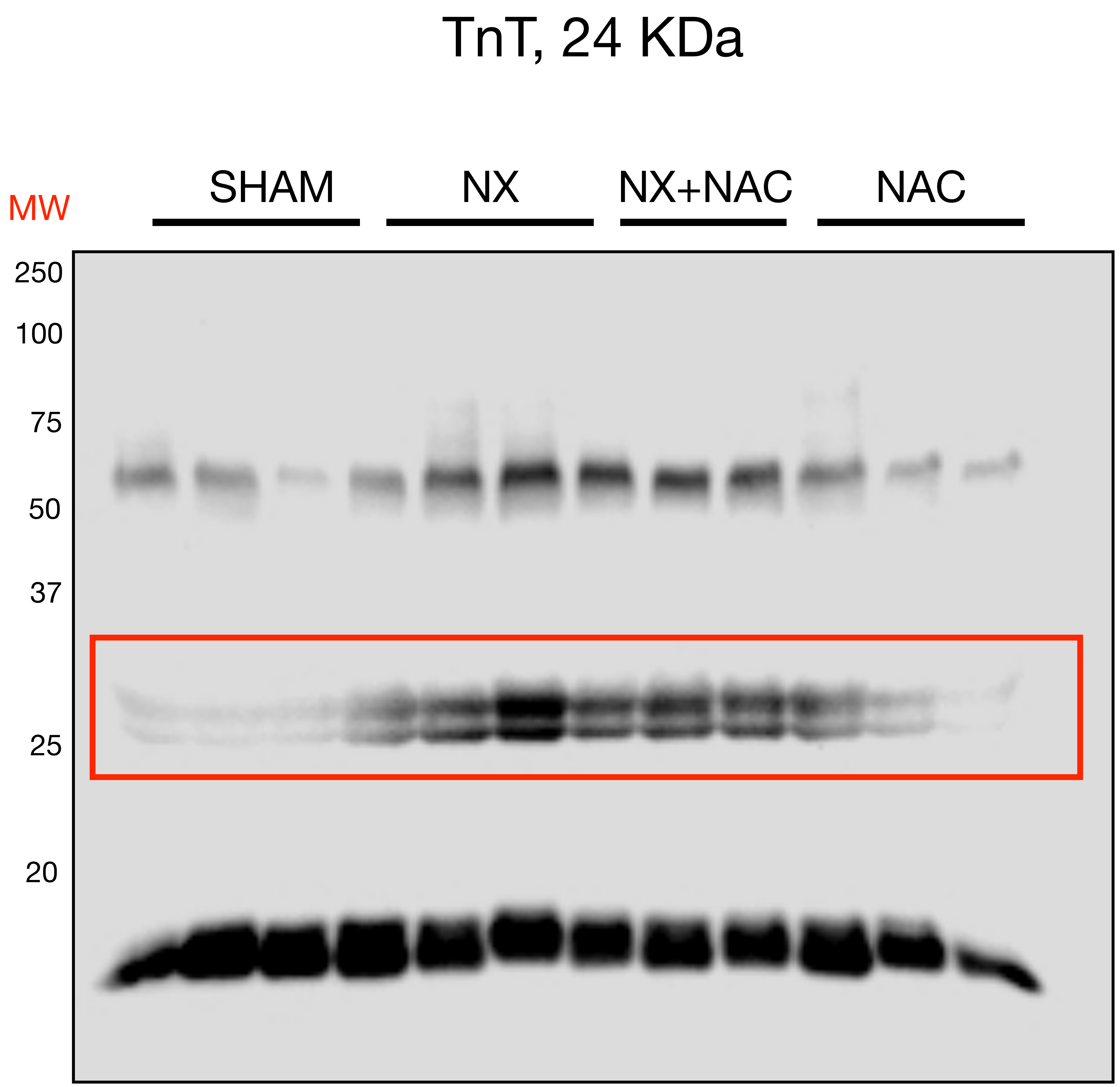

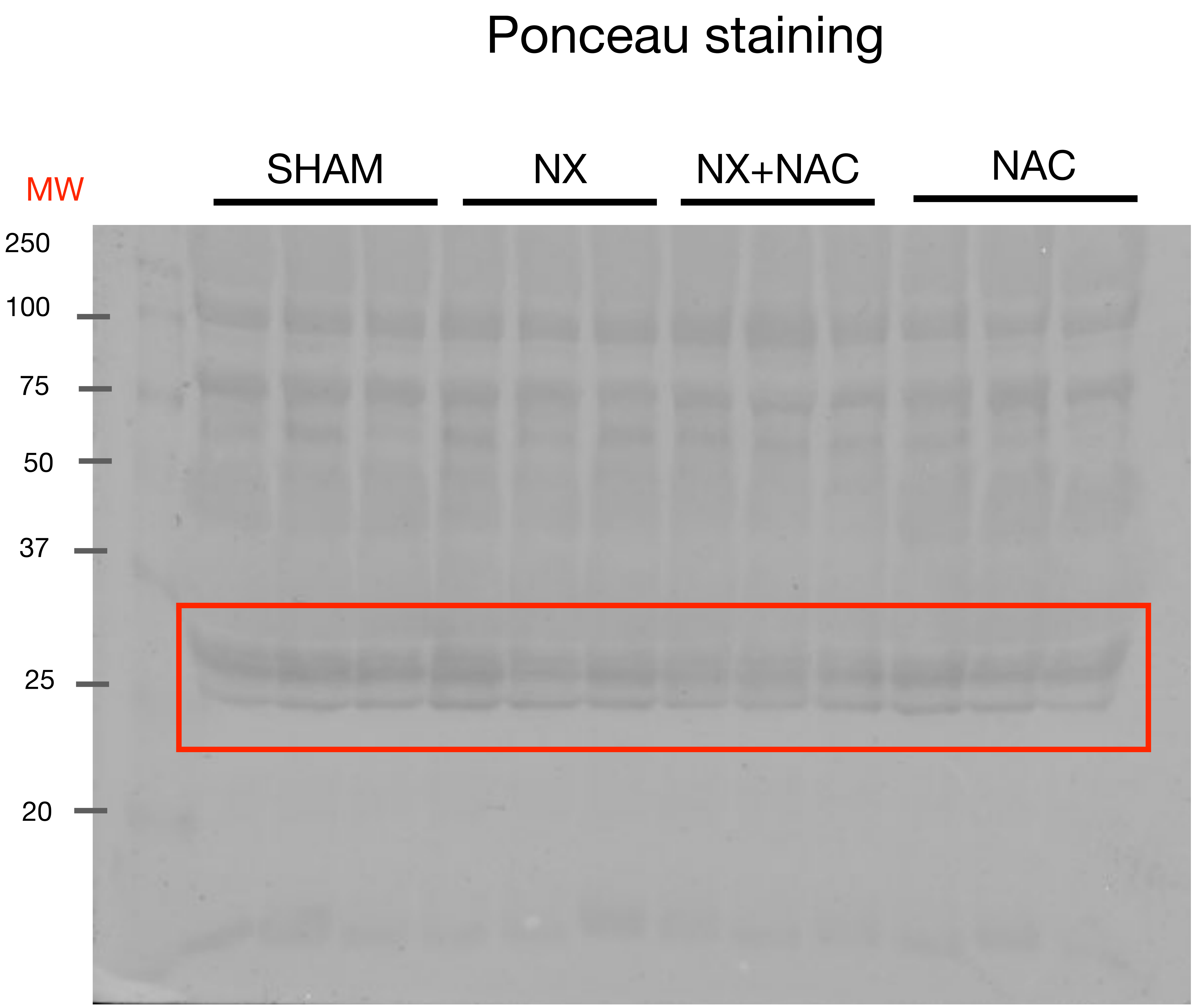

OXPHOS

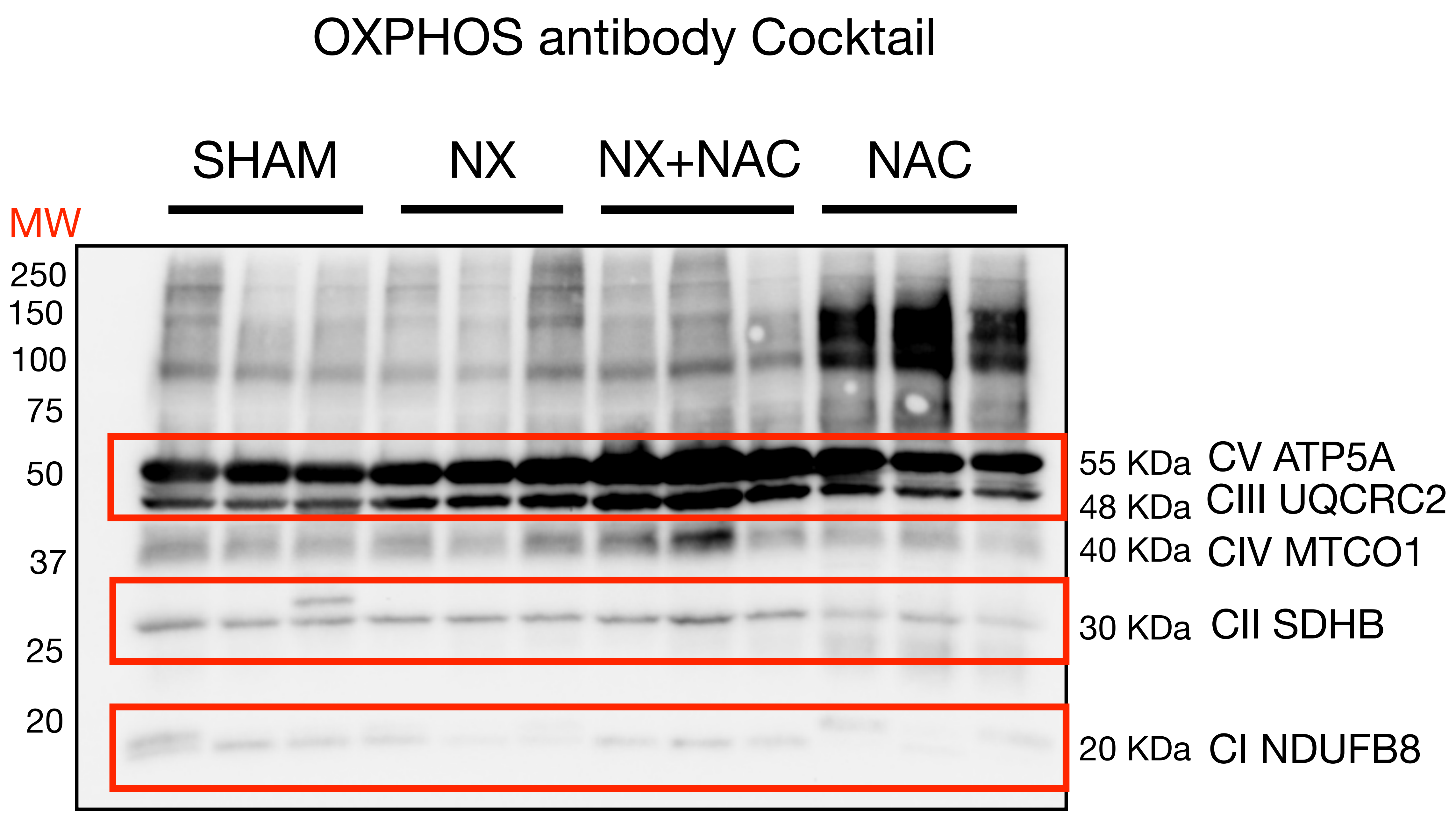

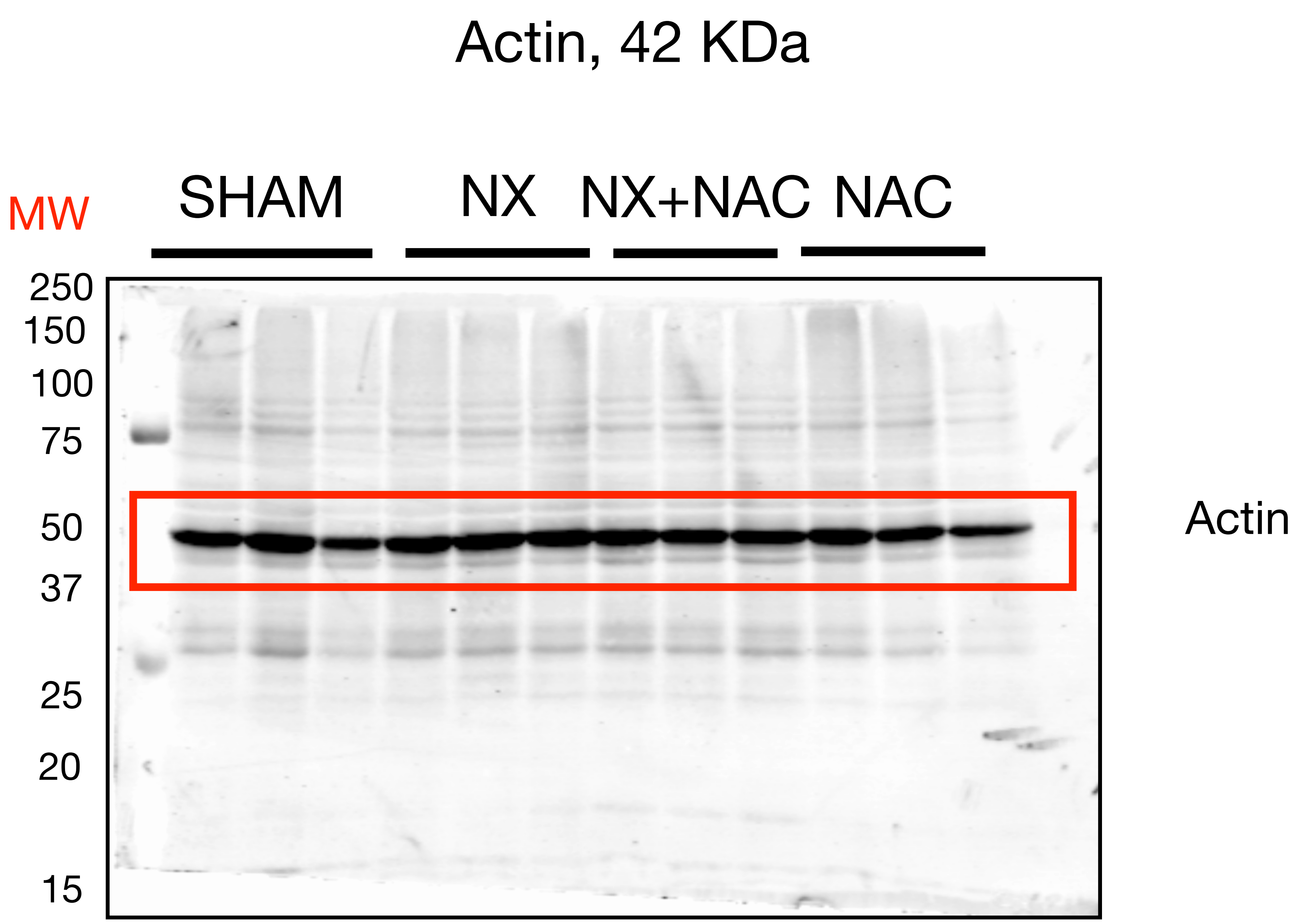

Chemiluminescence

GCLM

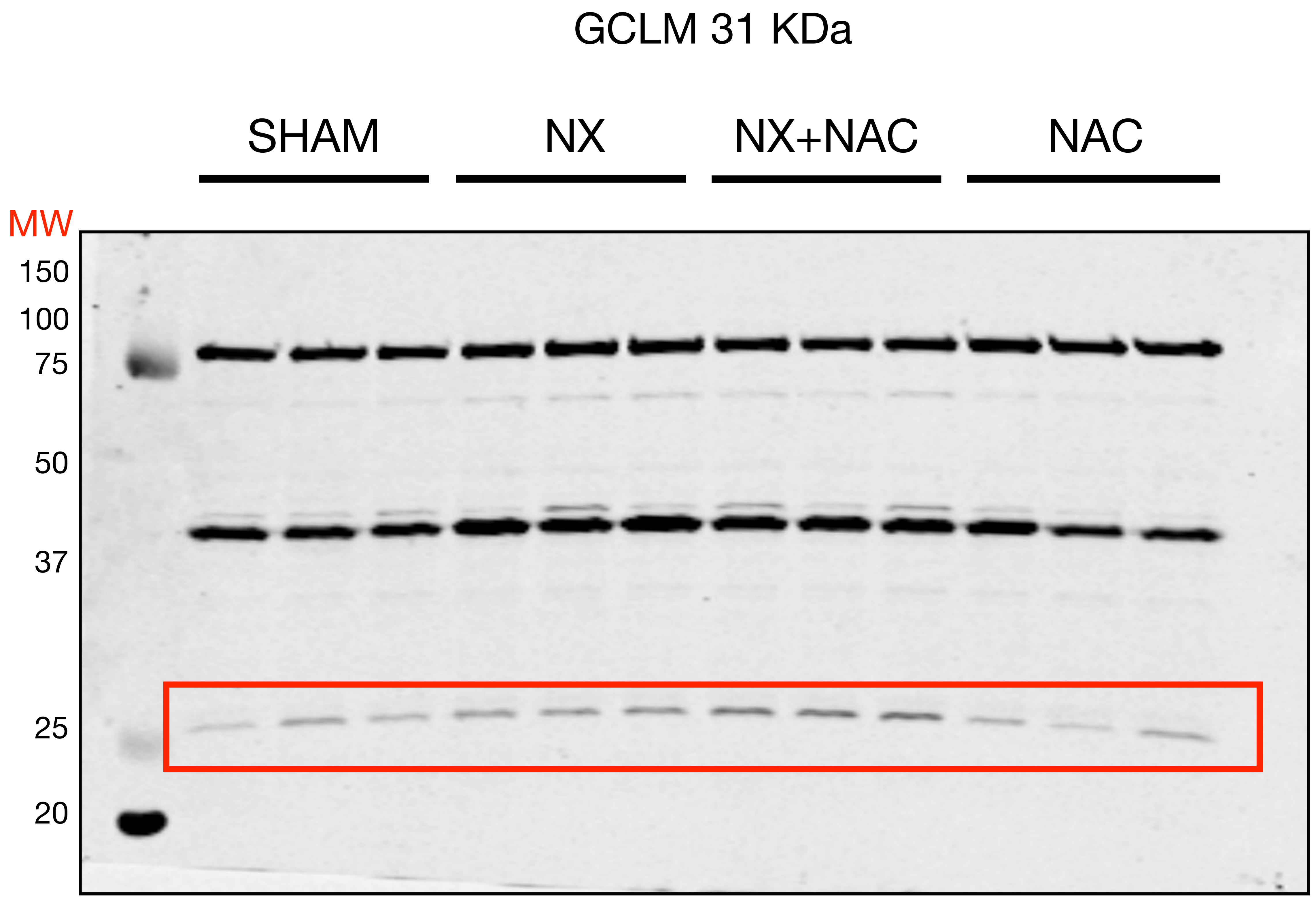
GCLM 31 KDa
SHAM
NX
NX+NAC
NAC
MW
150
100
75
50
37
25
20

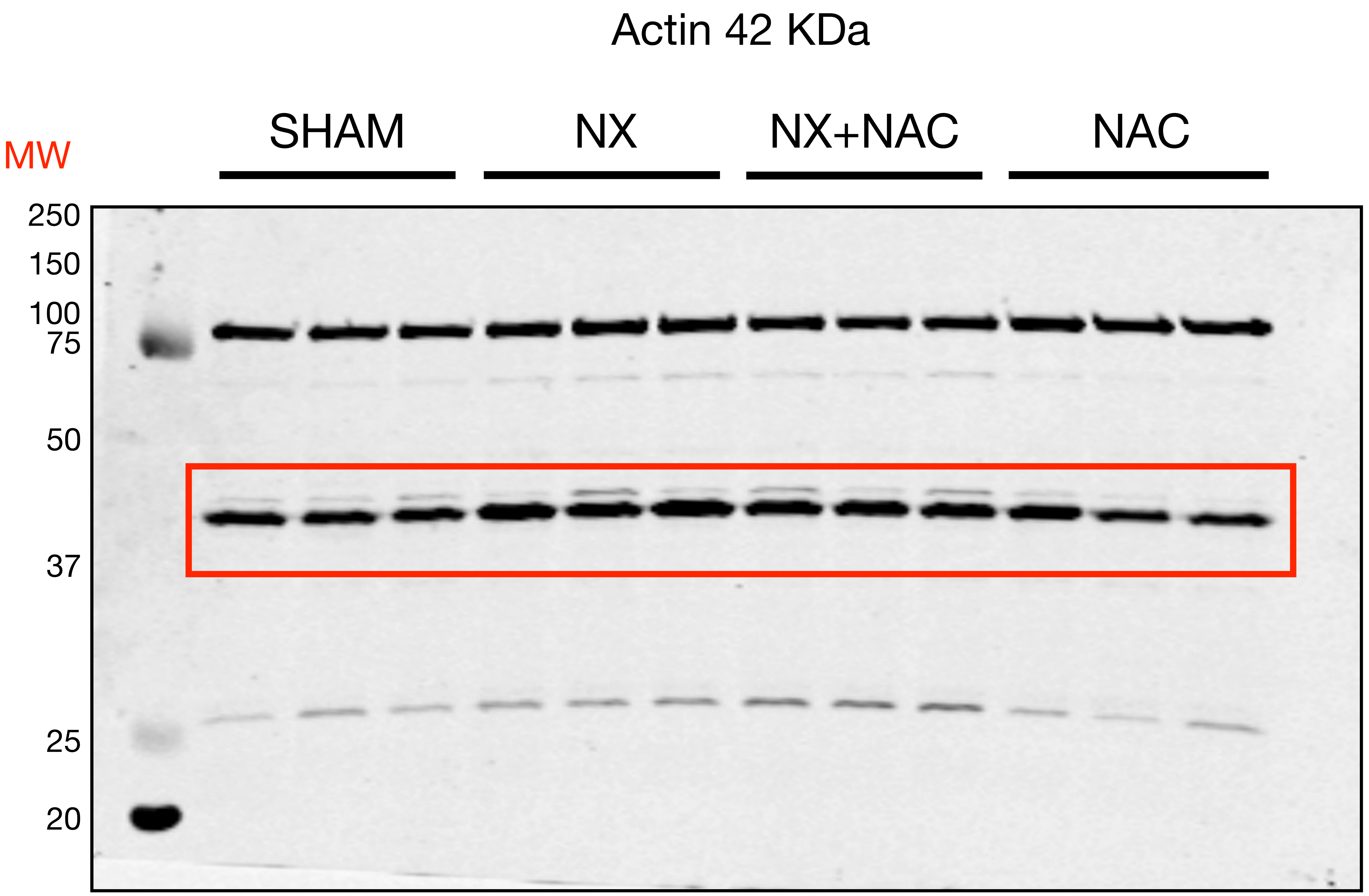
Actin 42 KDa
SHAM
NX
NX+NAC
NAC
MW
250
150
100
75
50
37
25
20

GCLC/ACTIN

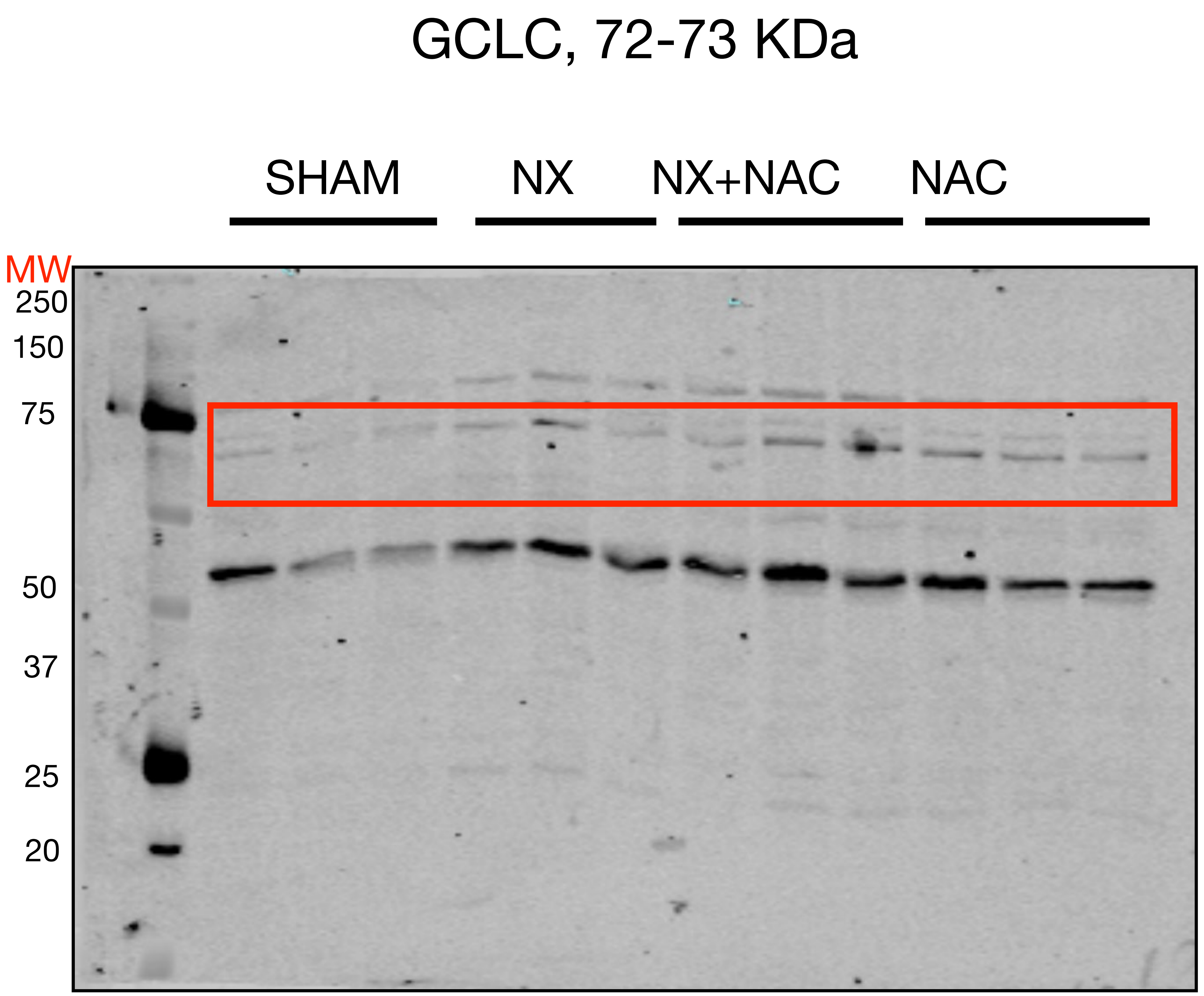
GCLC, 72-73 KDa
SHAM
NX
NX+NAC
NAC
MW
250
150
75
50
37
25
20

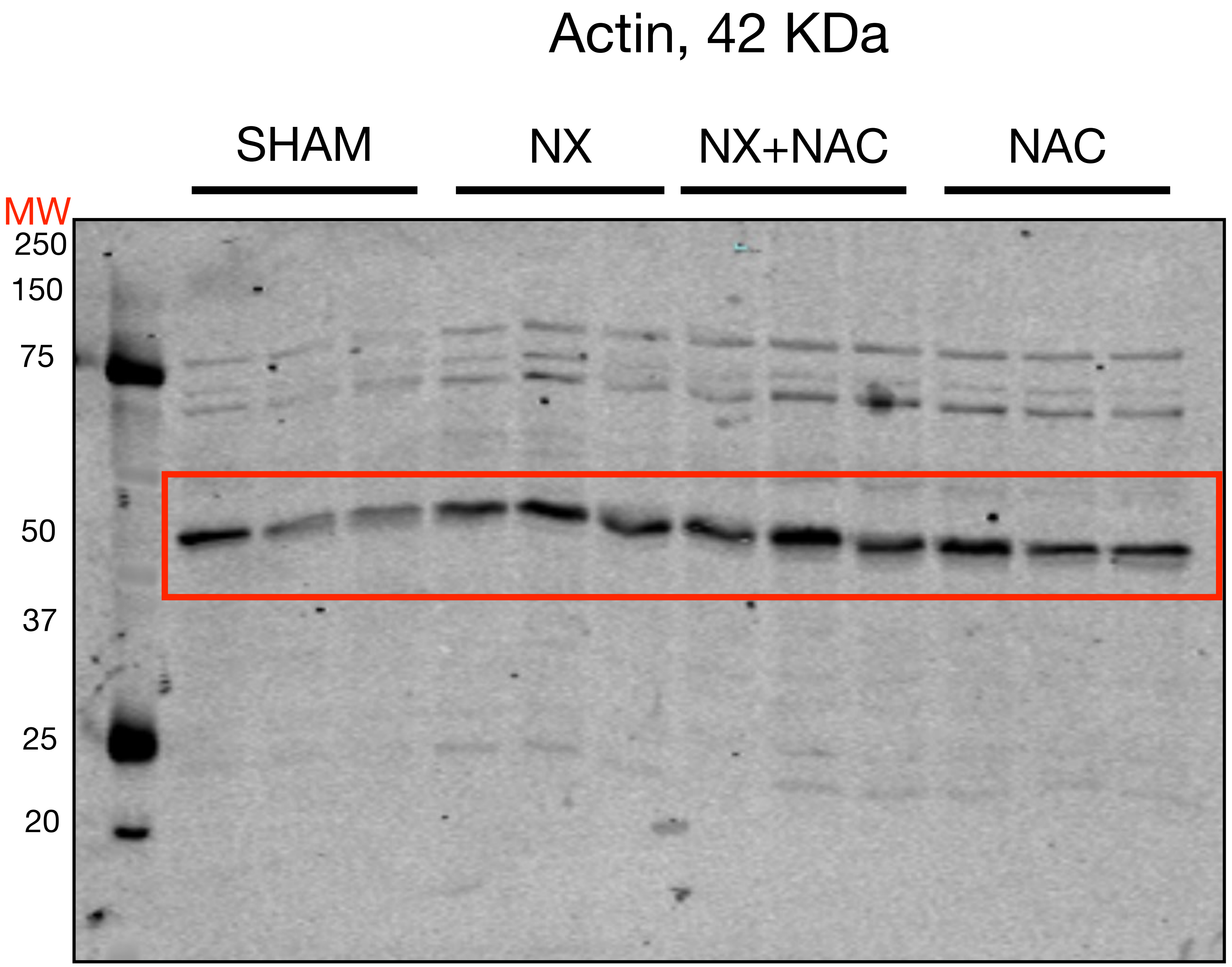
Actin, 42 KDa
SHAM
NX
NX+NAC
NAC
MW
250
150
75
50
37
25
20

GCLC/ACTIN

GCLC, 72-73 KDa

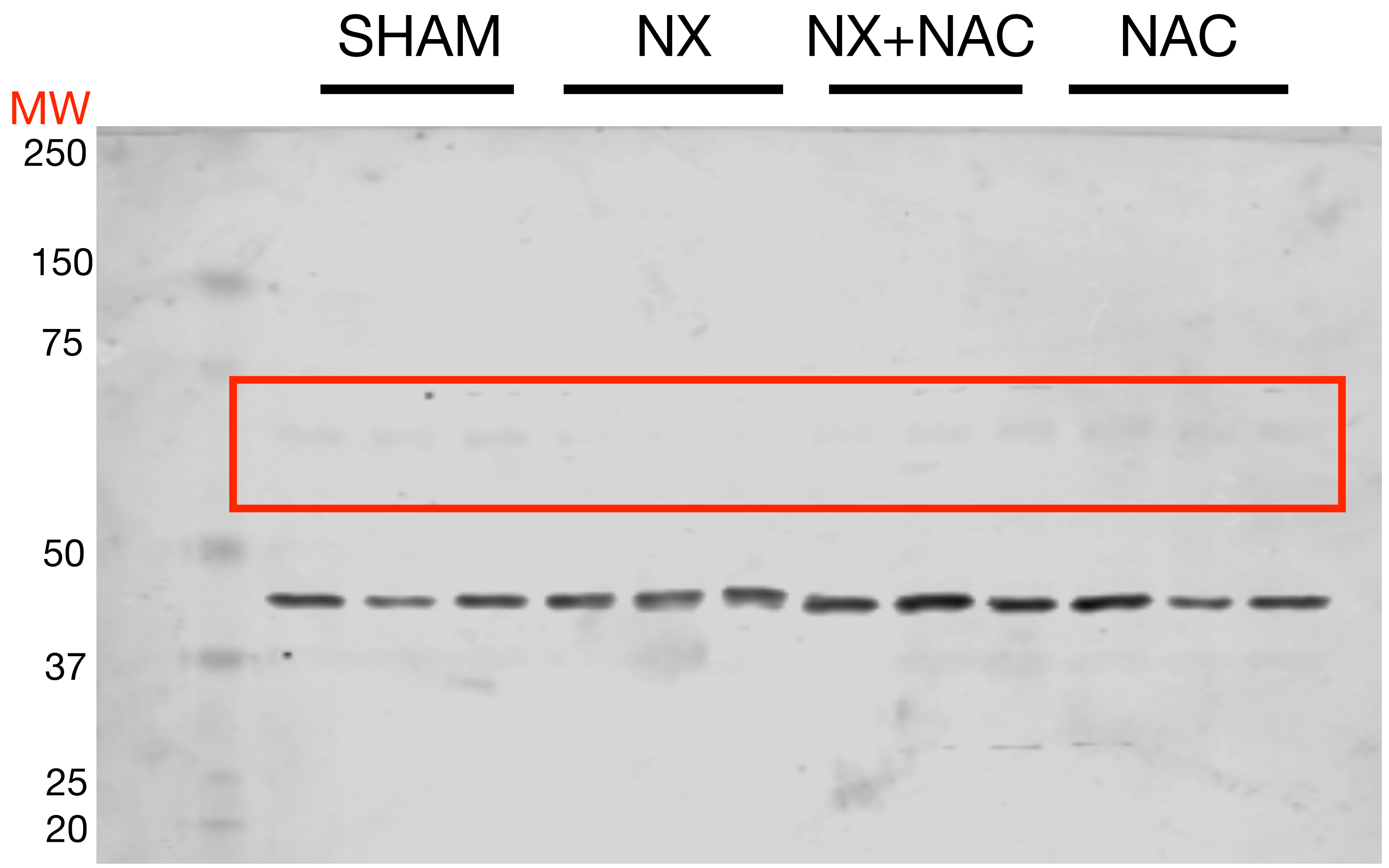

Actin, 42 KDa

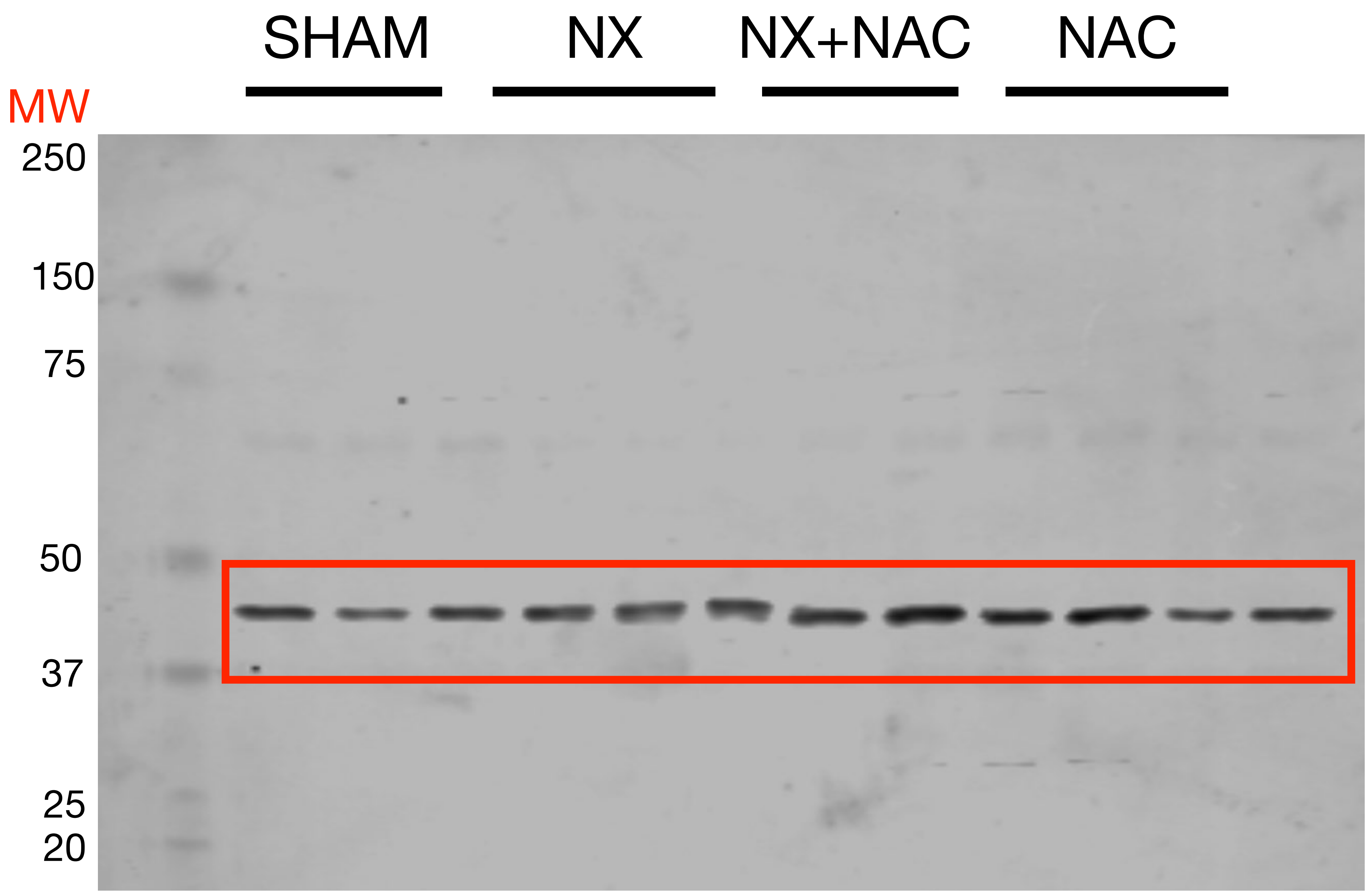

S-glutathionylation

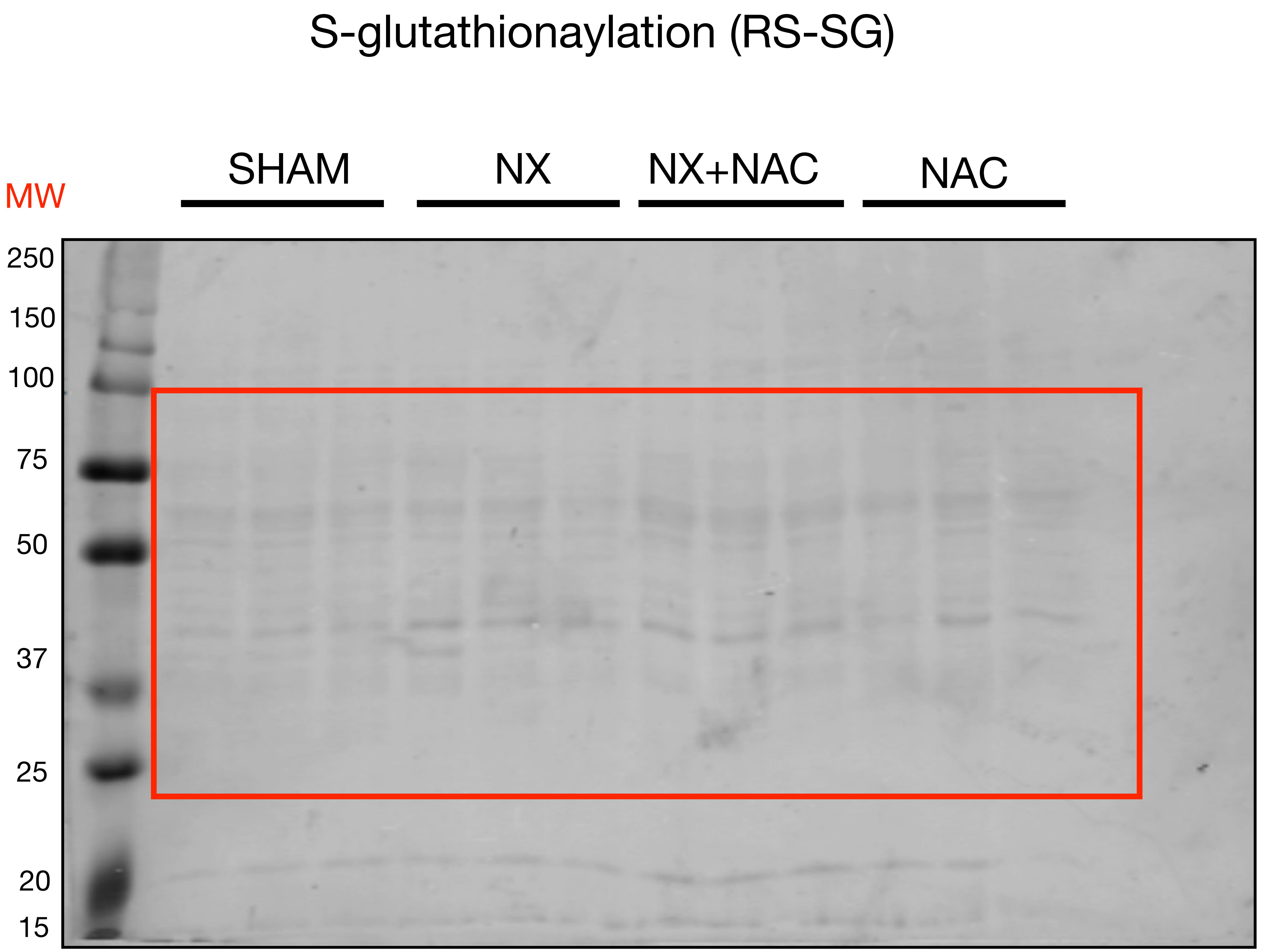

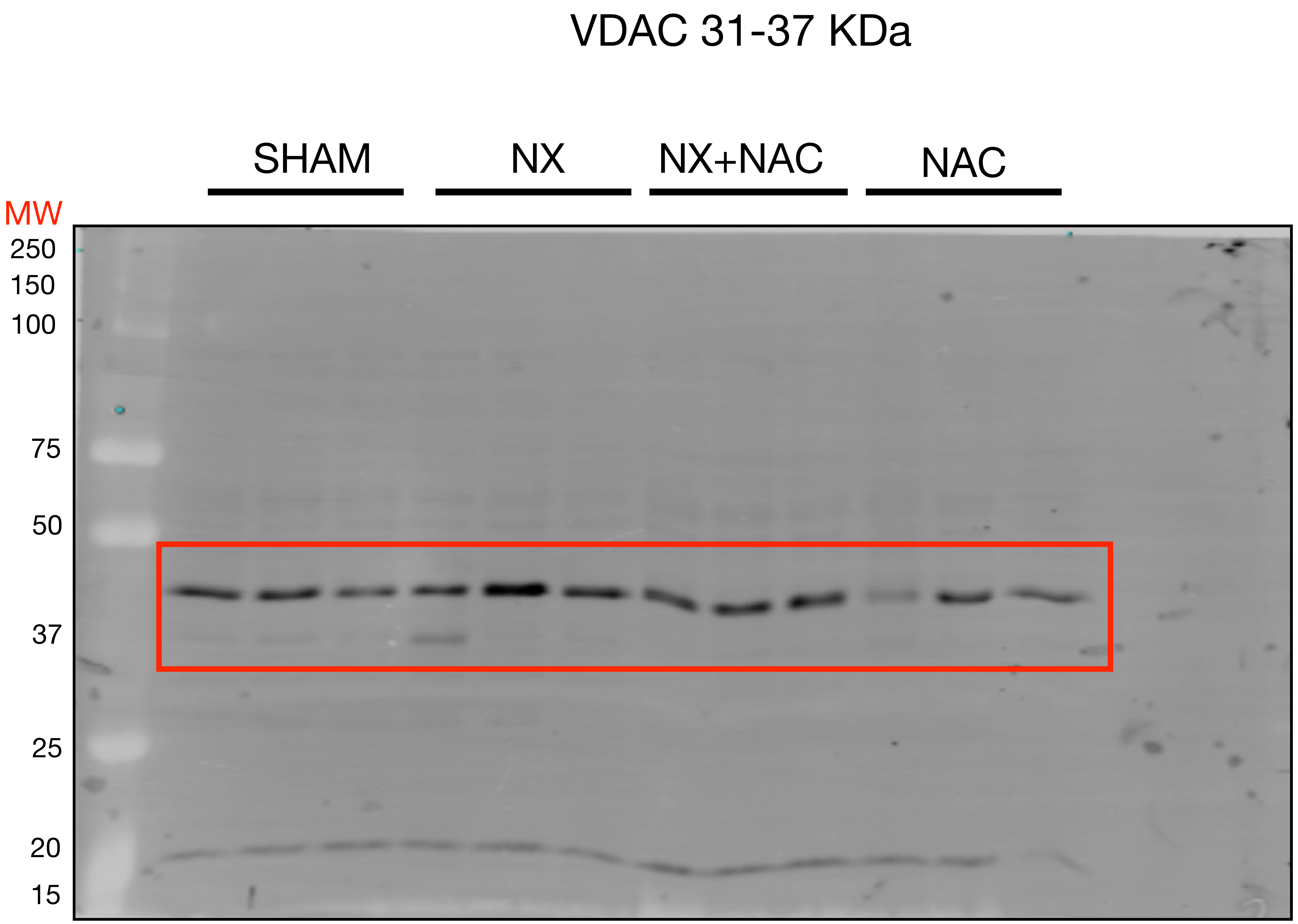

pAMPK (Thr172)/AMPK/ACTIN

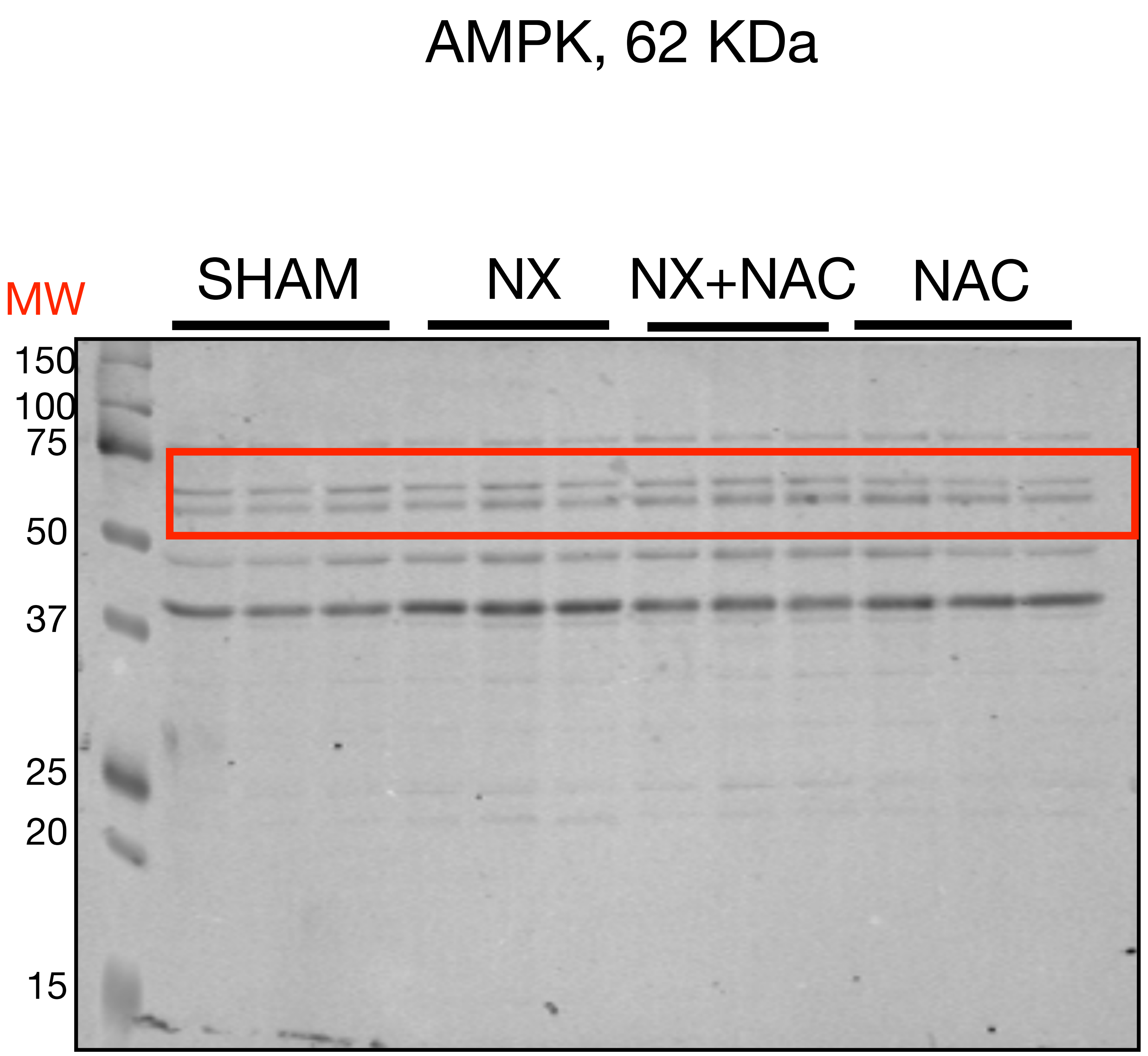

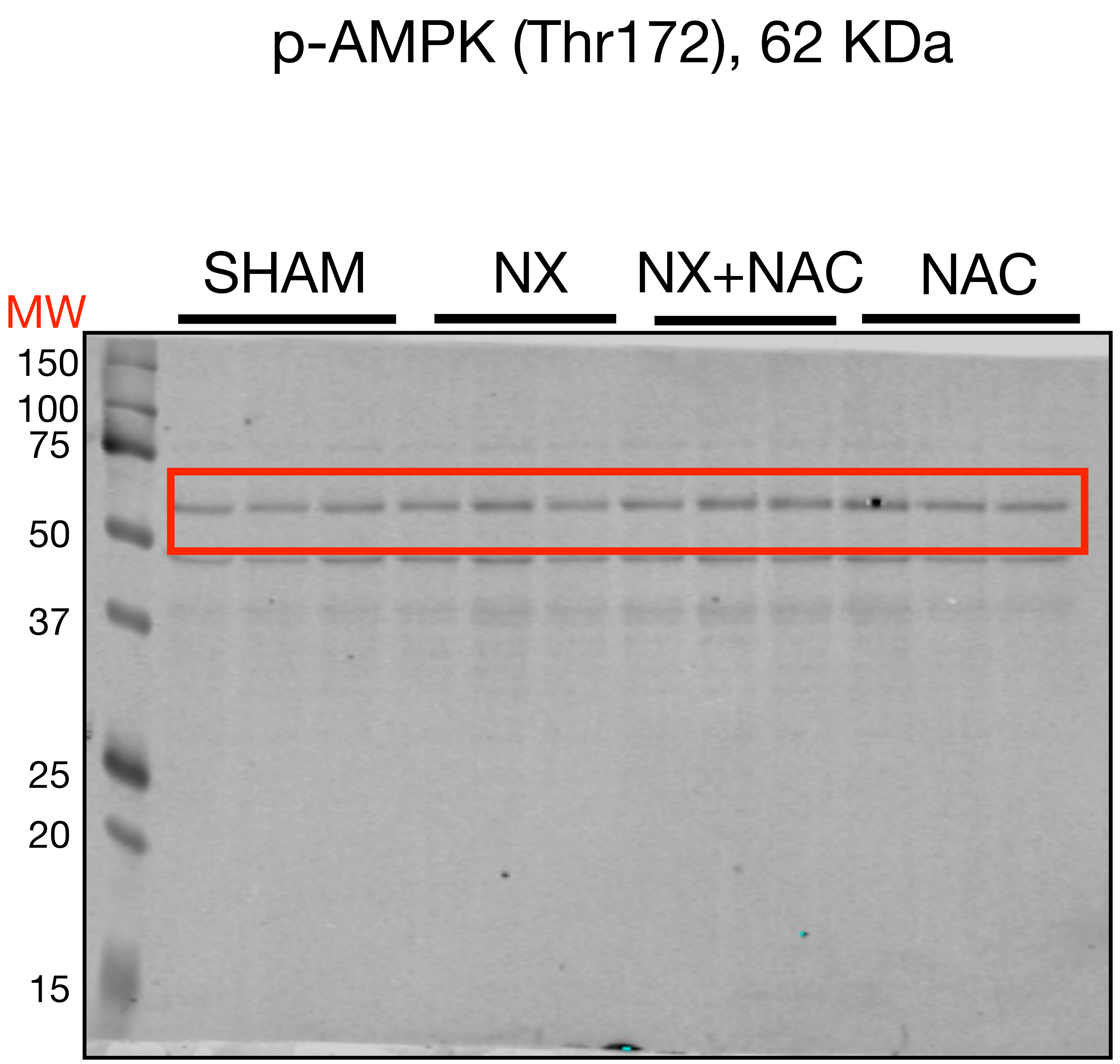

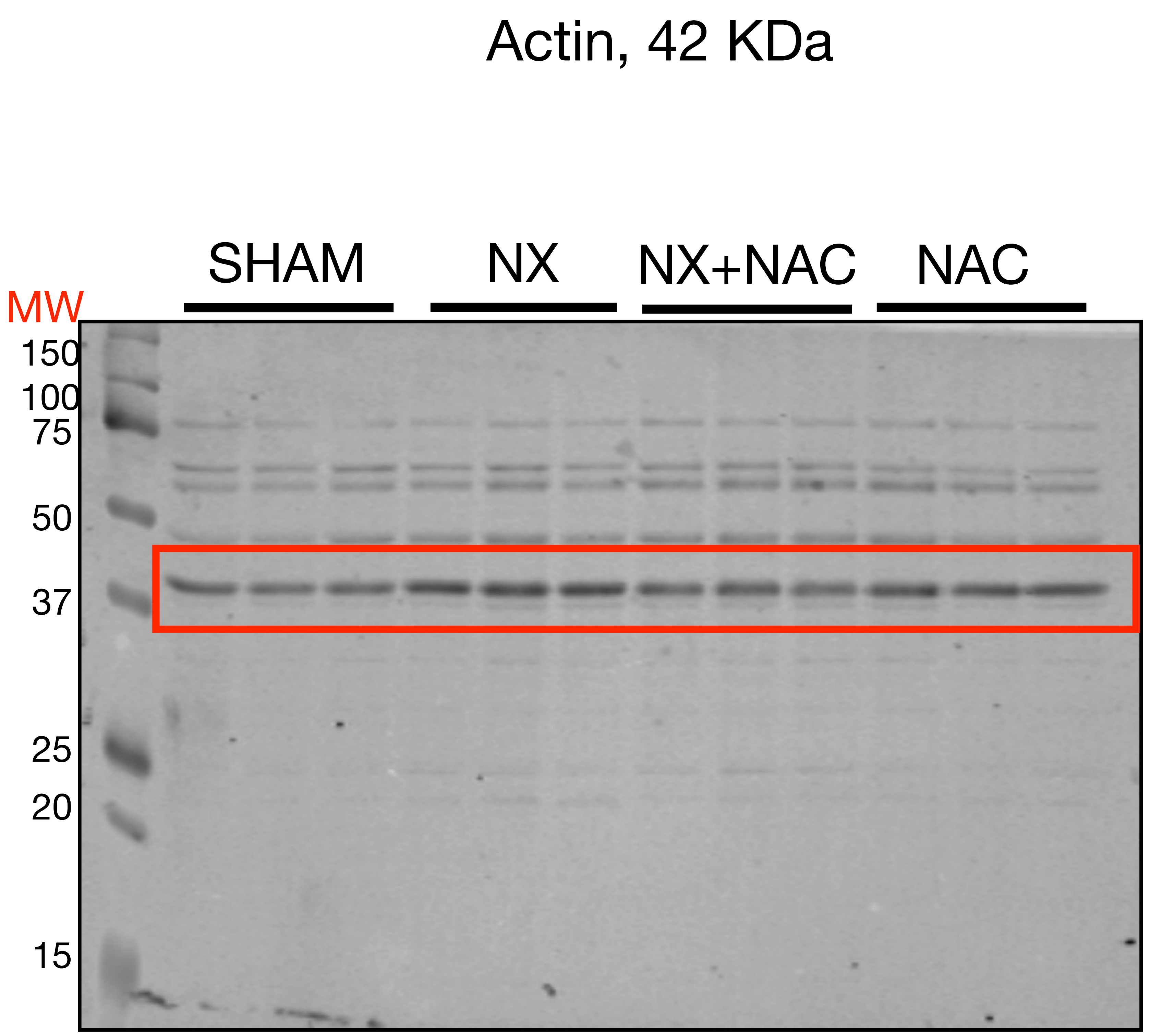

pAMPK (Thr172)/AMPK/ACTIN

pAMPK, 62 KDa

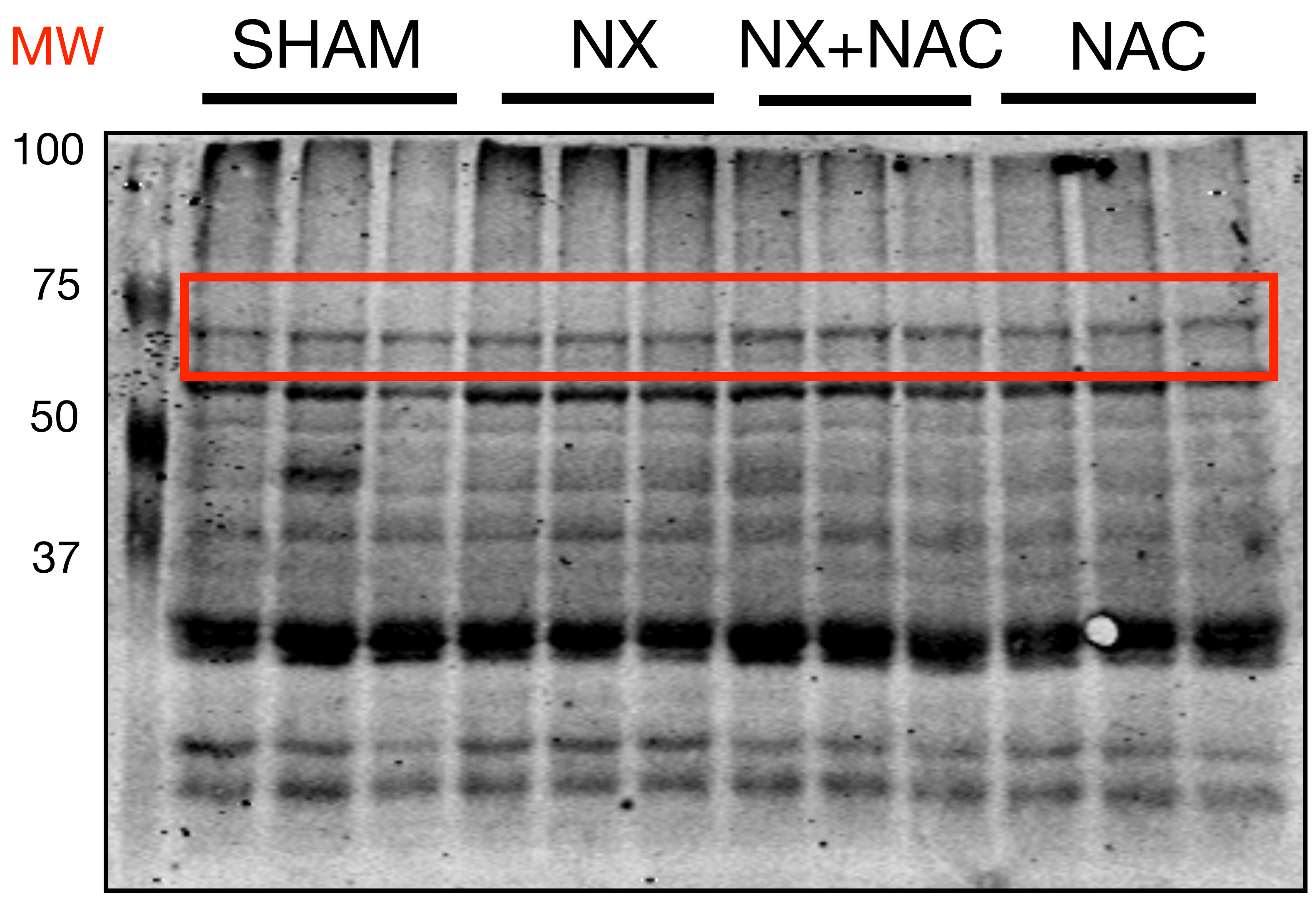

AMPK, 62 KDa

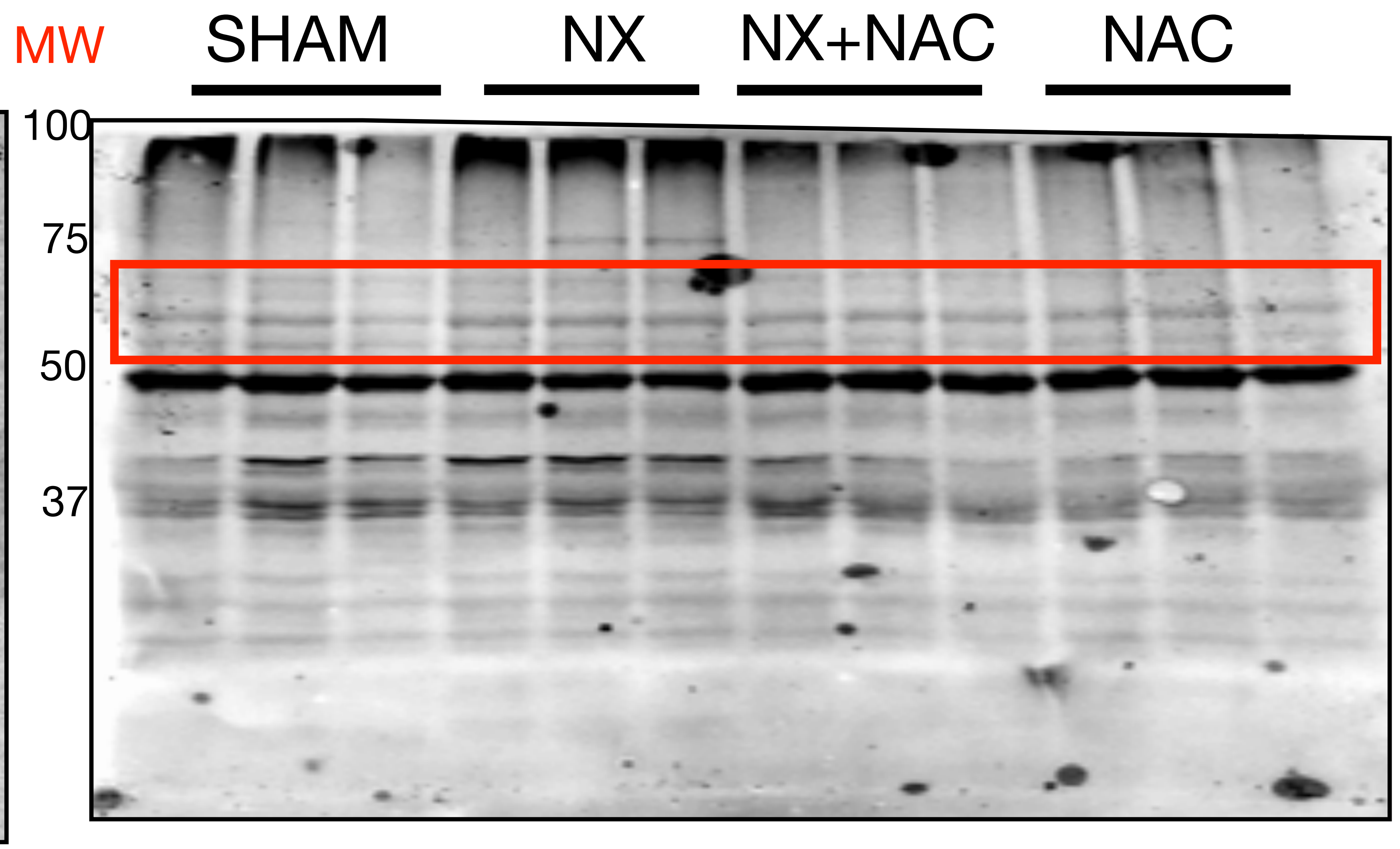

Actin, 42KDa

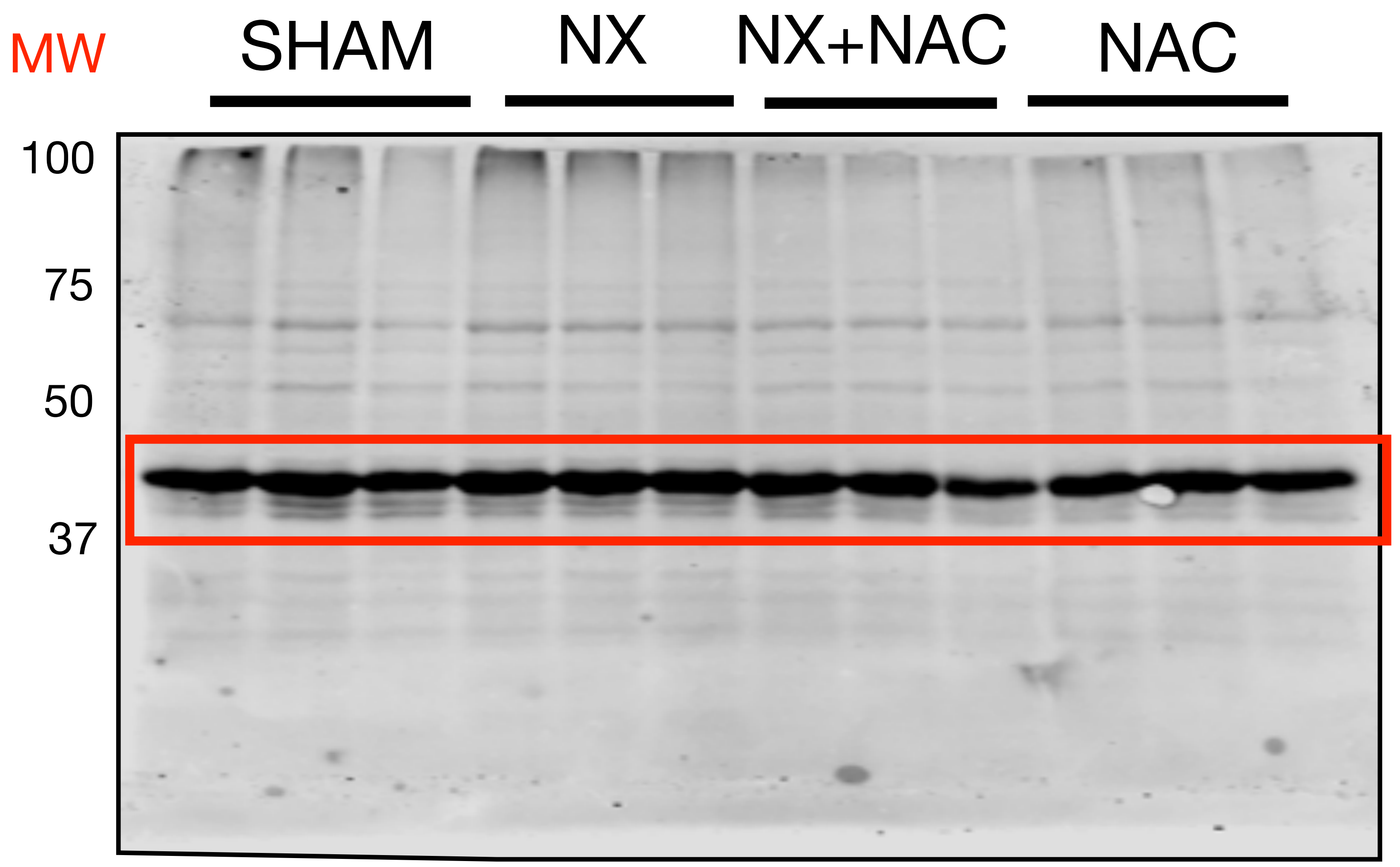

PGC1alpha

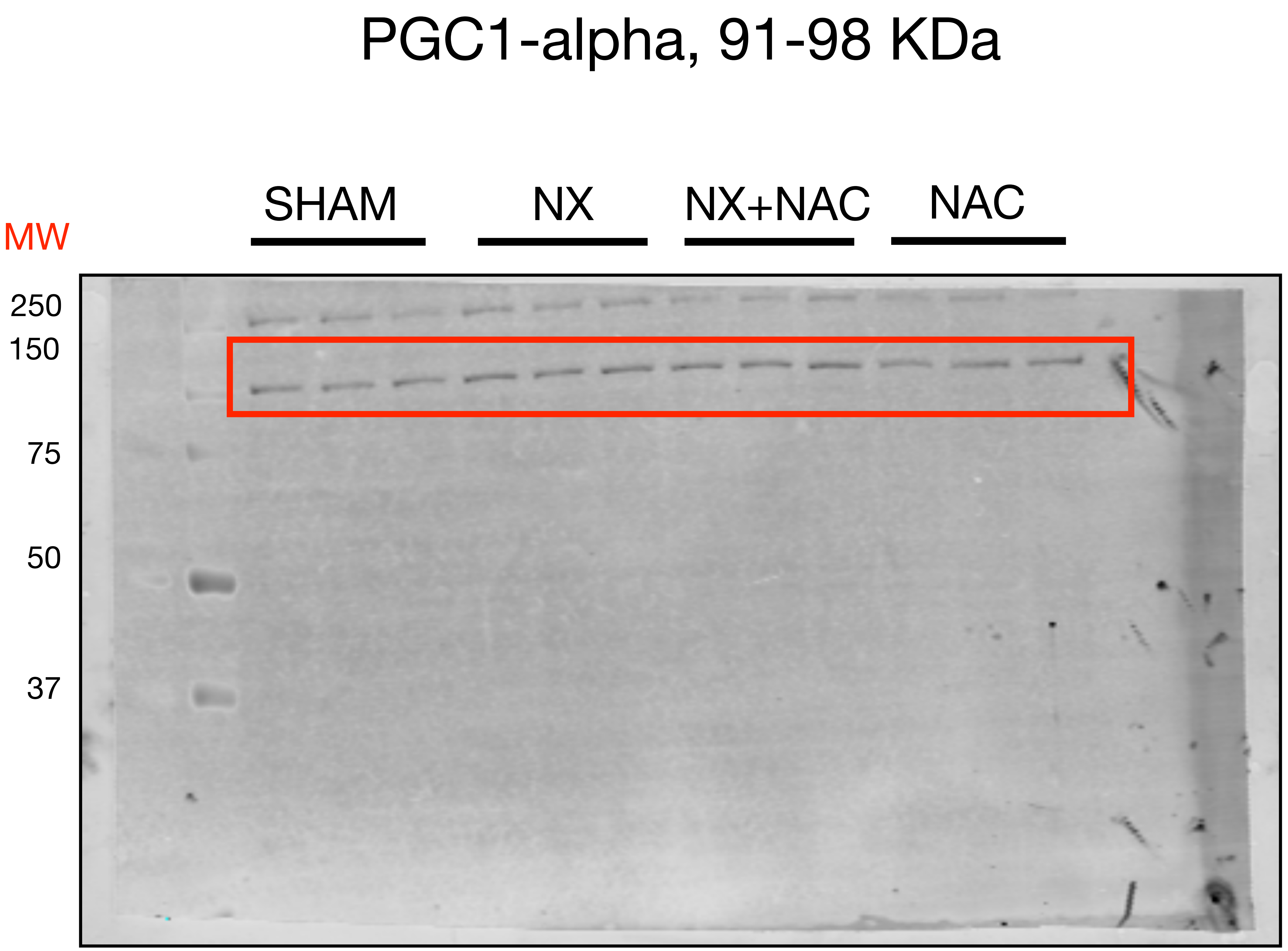
PGC1-alpha, 91-98 KDa
SHAM
NX
NX+NAC
NAC
MW
250
150
75
50
37

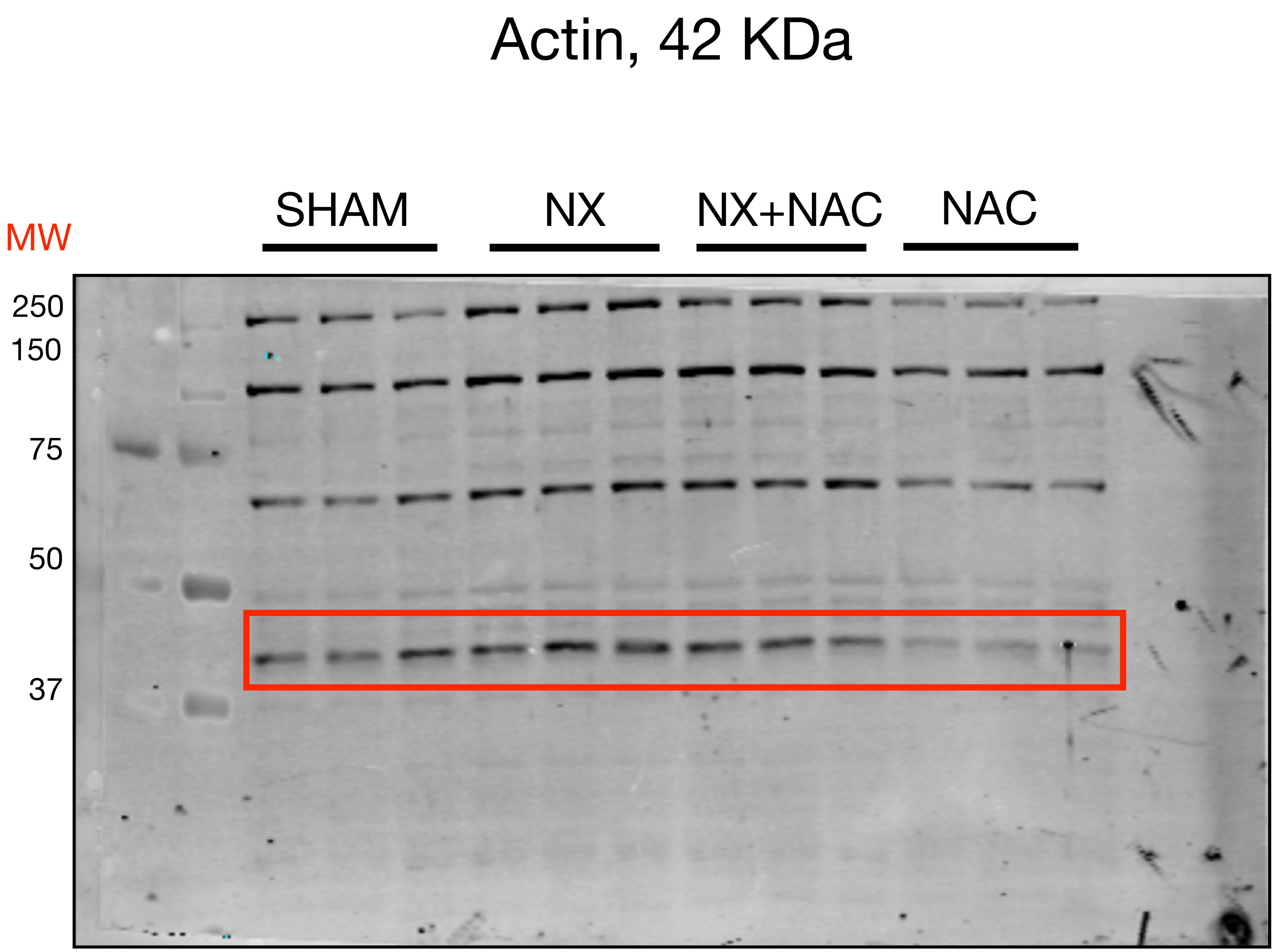
Actin, 42 KDa
SHAM
NX
NX+NAC
NAC
MW
250
150
75
50
37

PGC1alpha

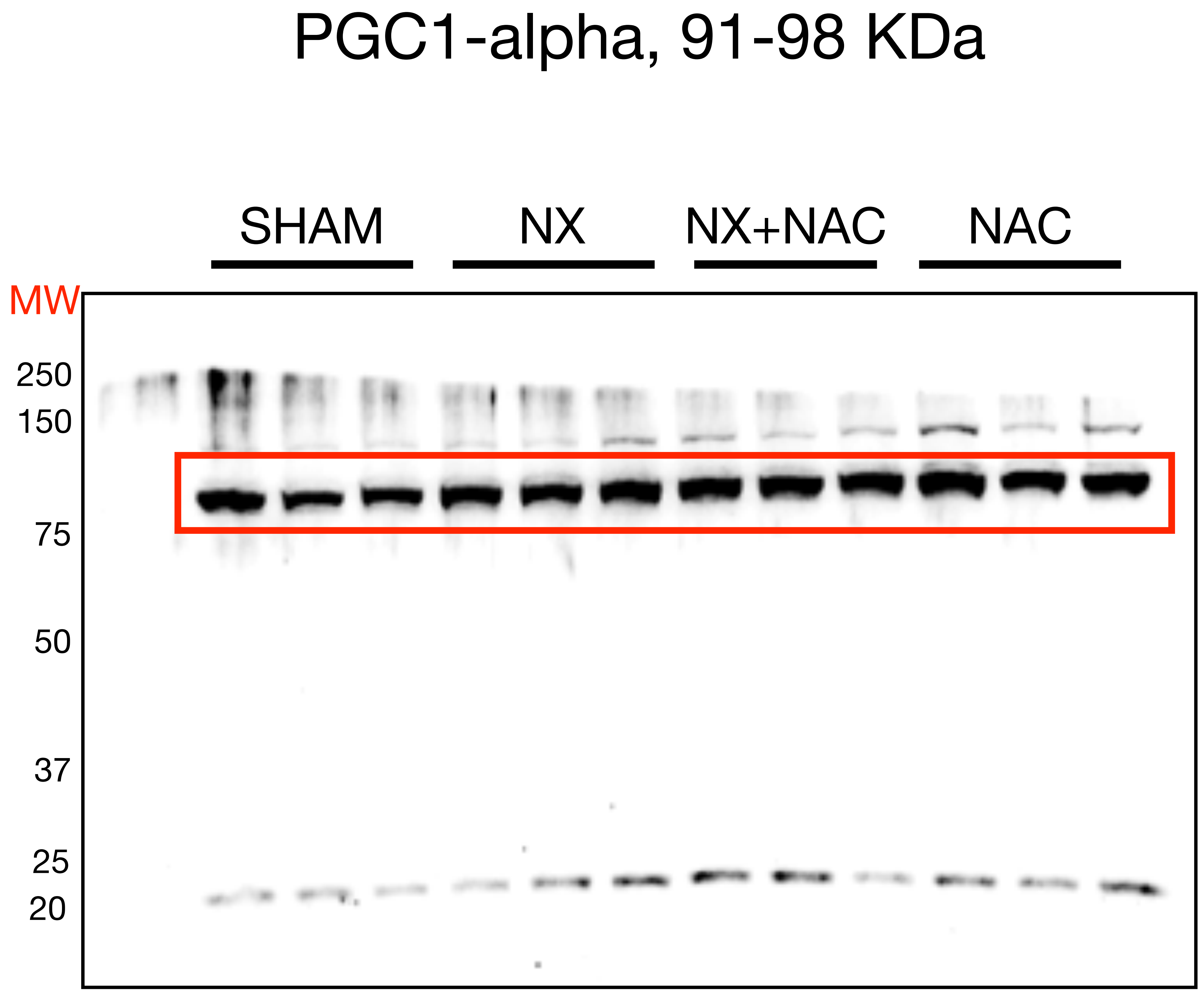

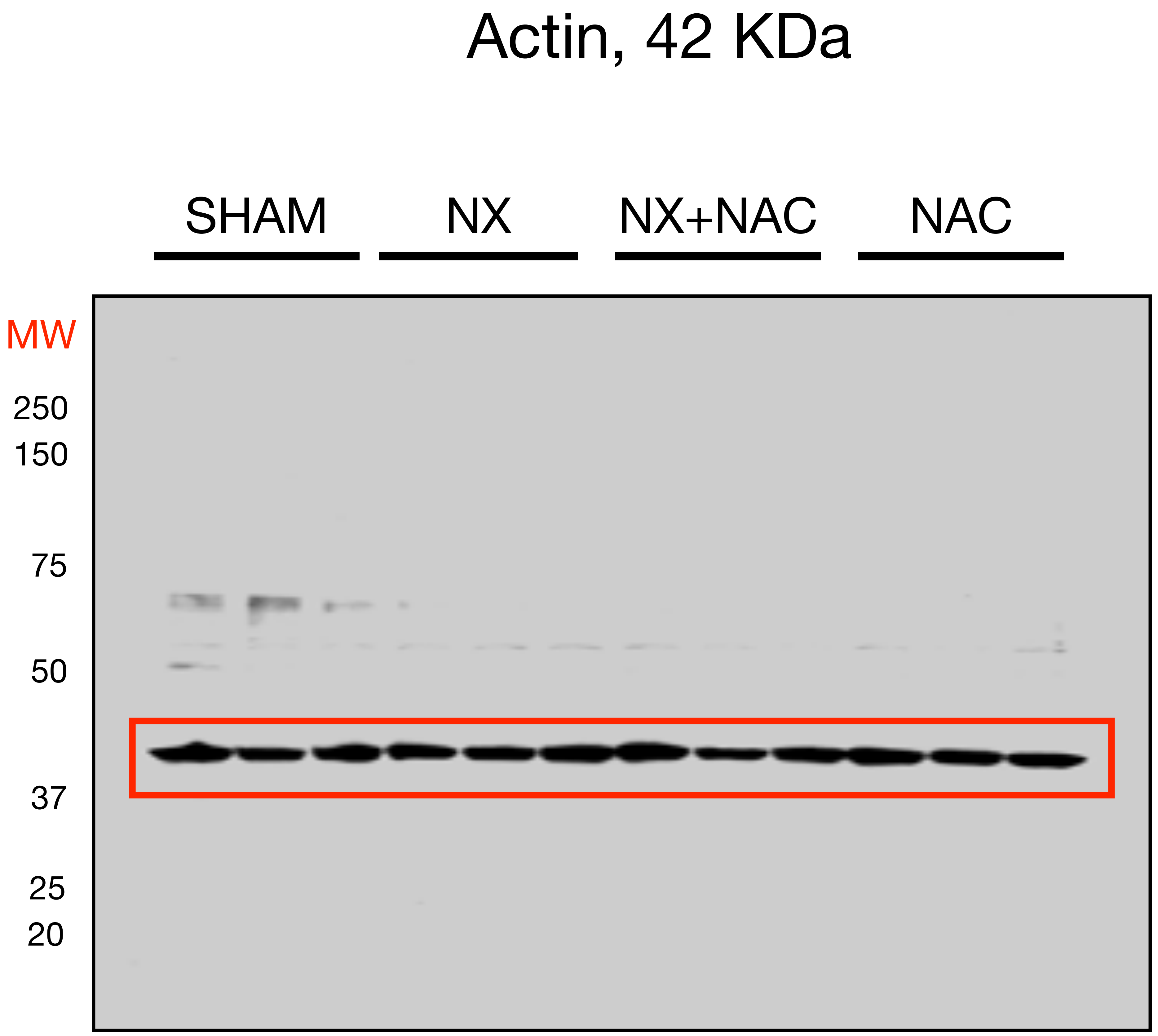

Chemiluminescence

SIRT3

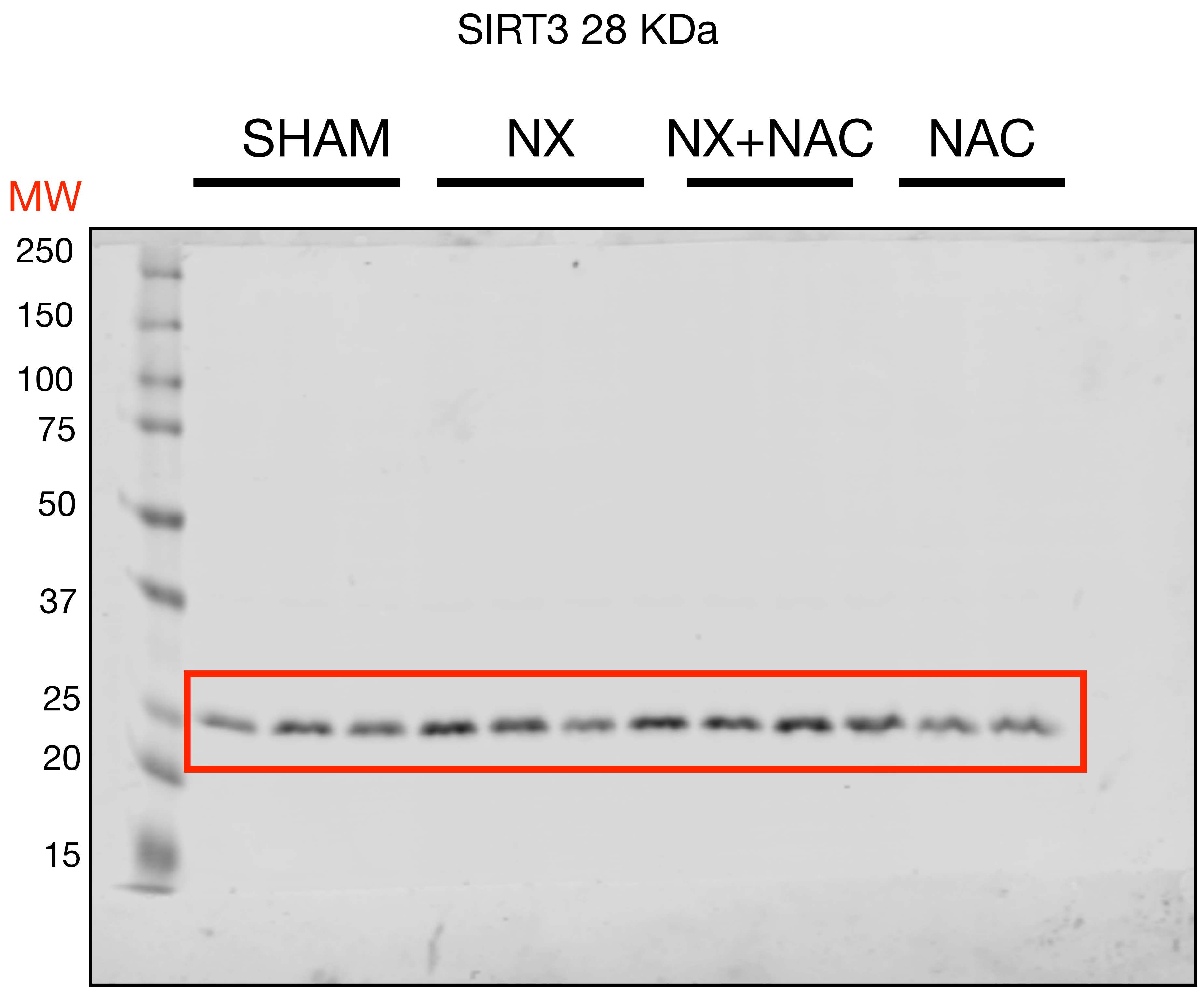
SIRT3 28 KDa
SHAM
NX
NX+NAC
NAC
MW
250
150
100
75
50
37
25
20
15

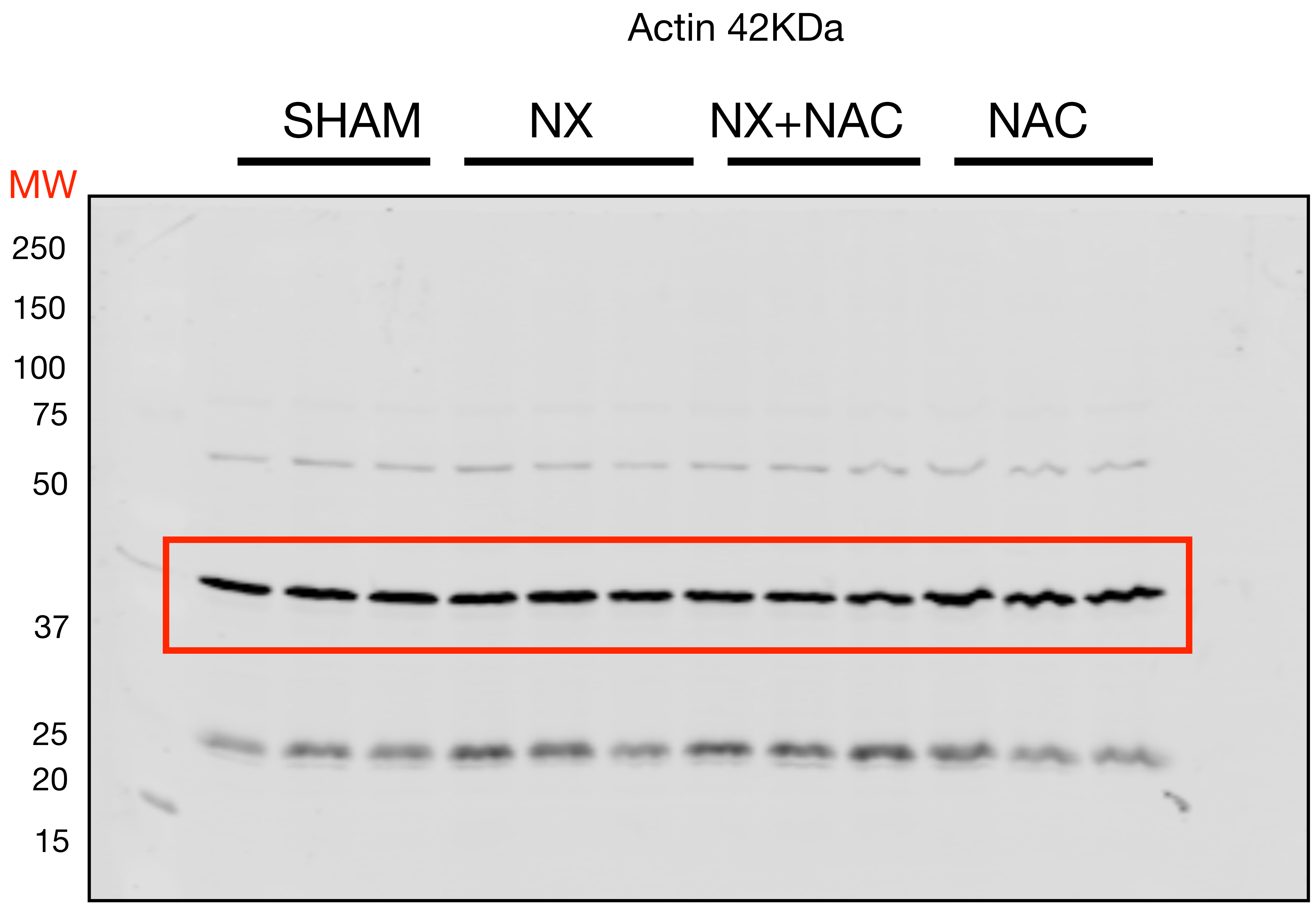
Actin 42KDa
SHAM
NX
NX+NAC
NAC
MW
250
150
100
75
50
37
25
20
15

SIRT3

Colorimetric

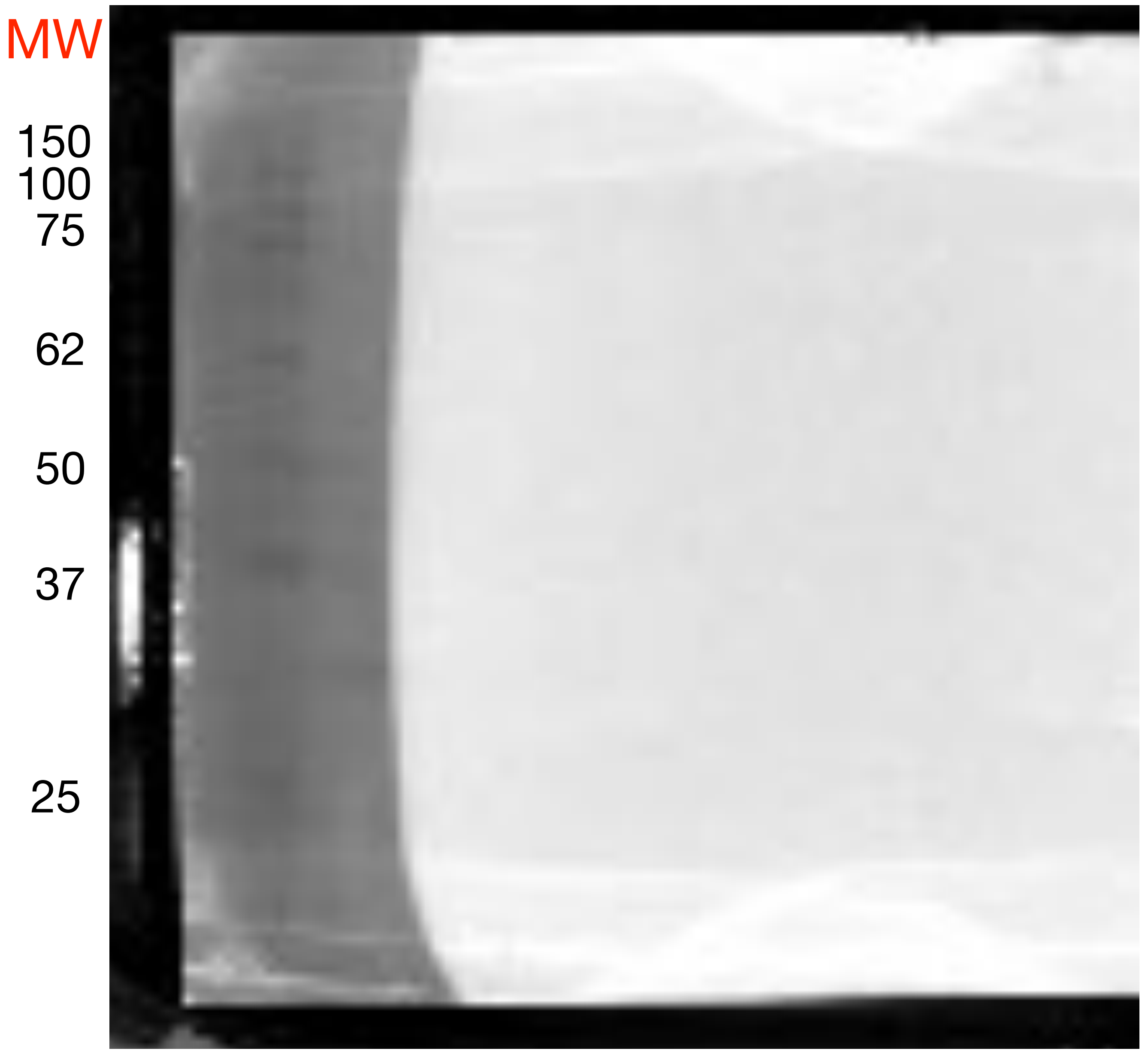

Sirt3, 28 KDa

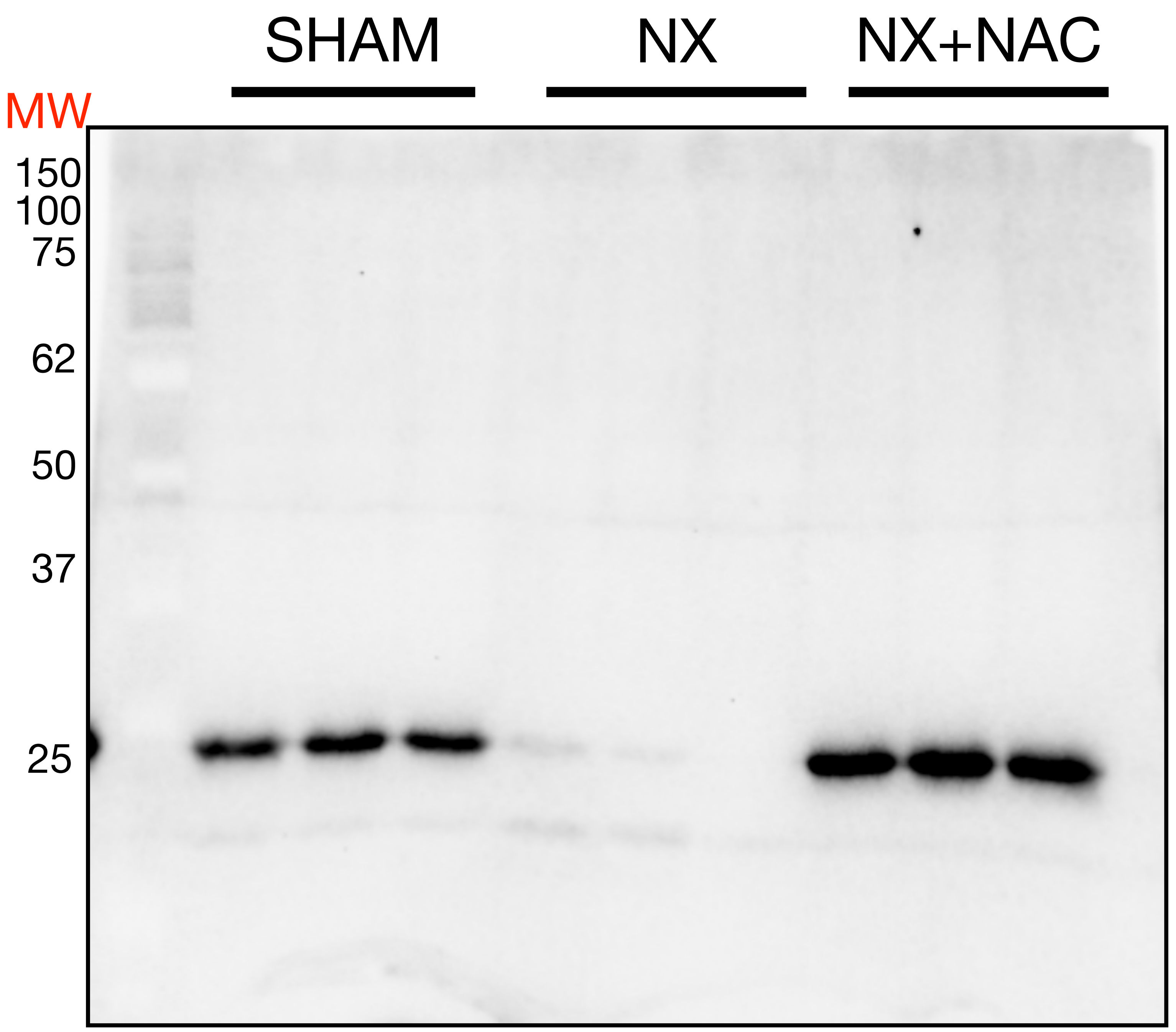

Actin, 42 KDa

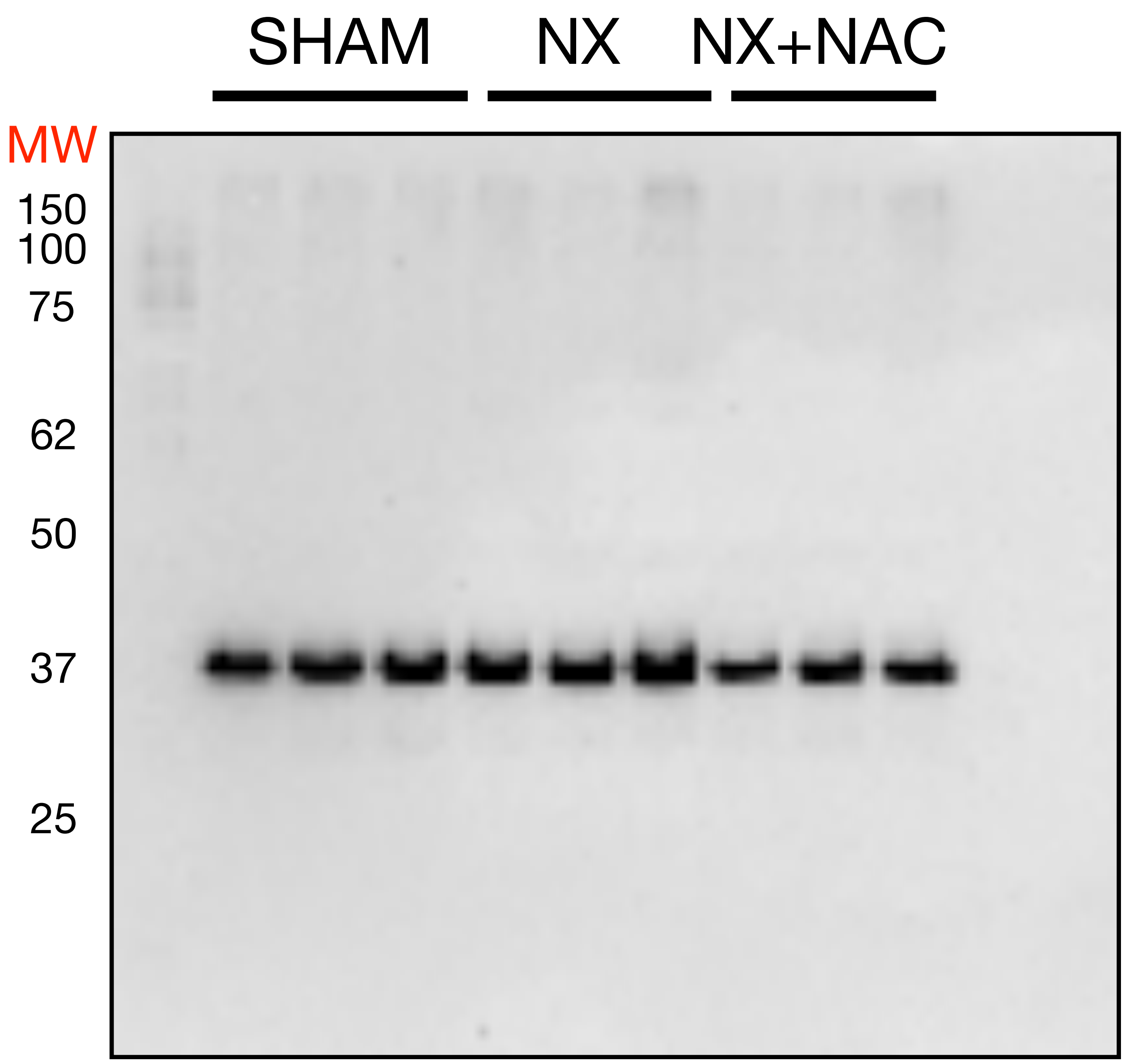

Chemiluminescence

Total acetylated lysines

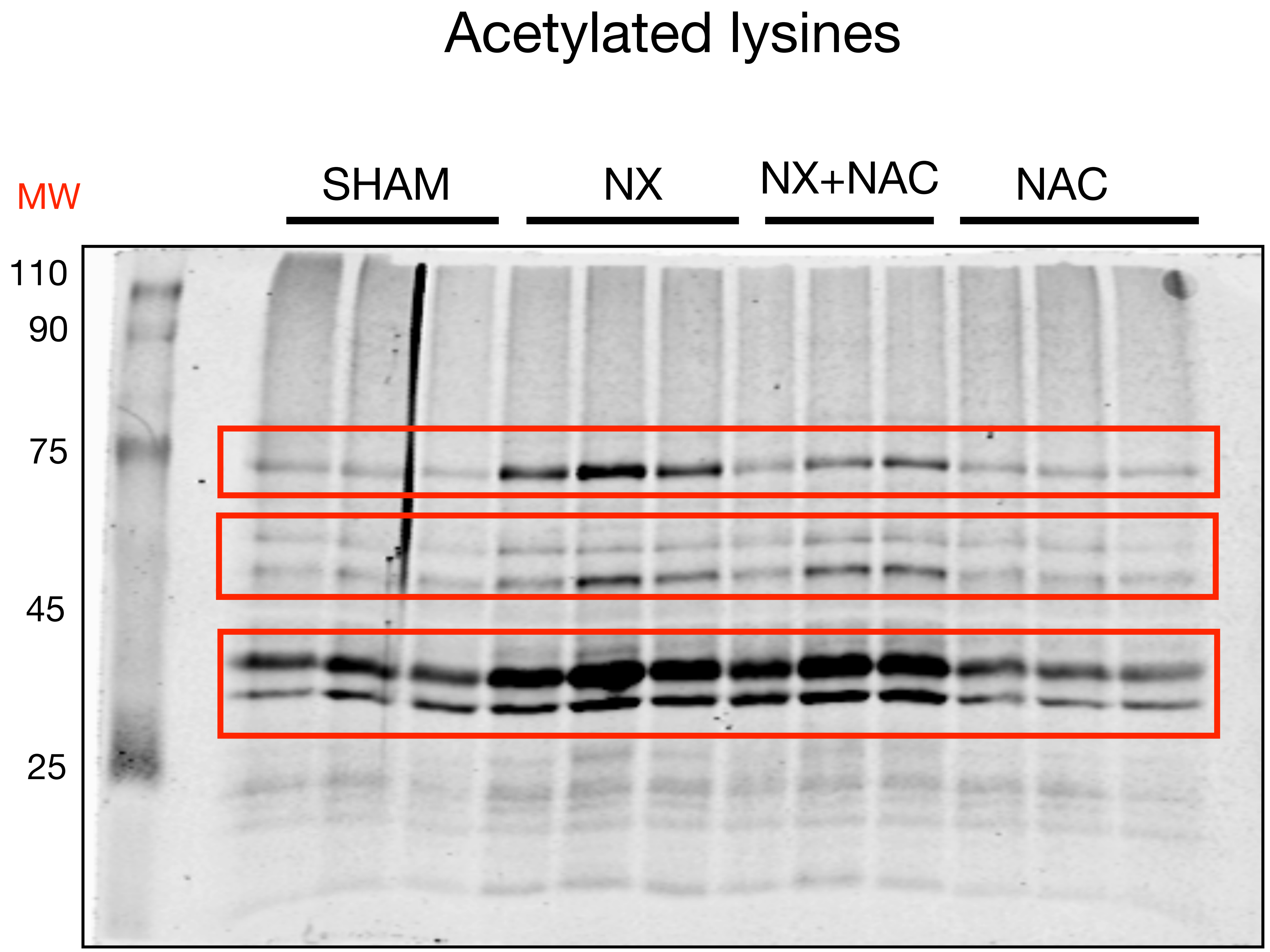

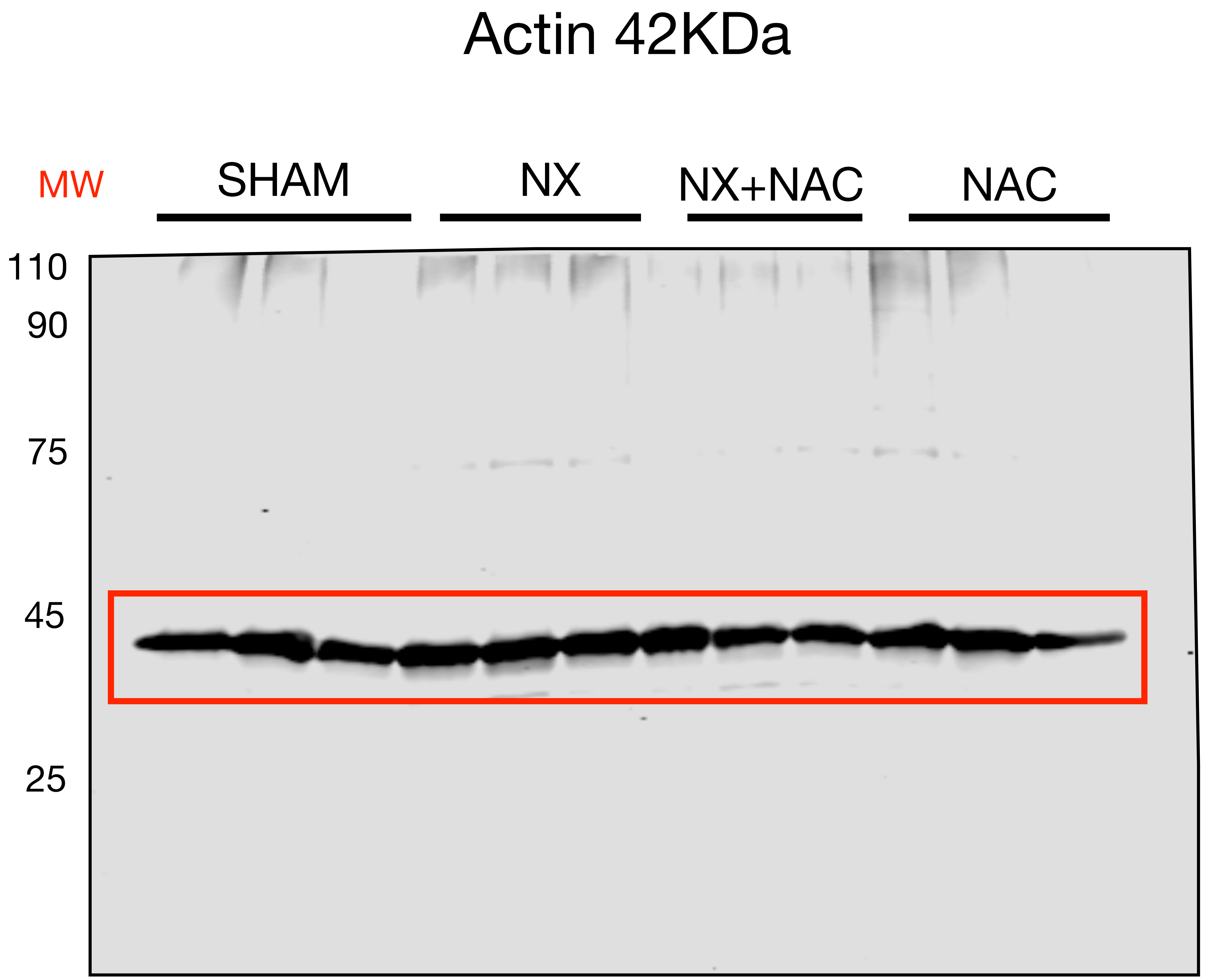

Total acetylated lysines

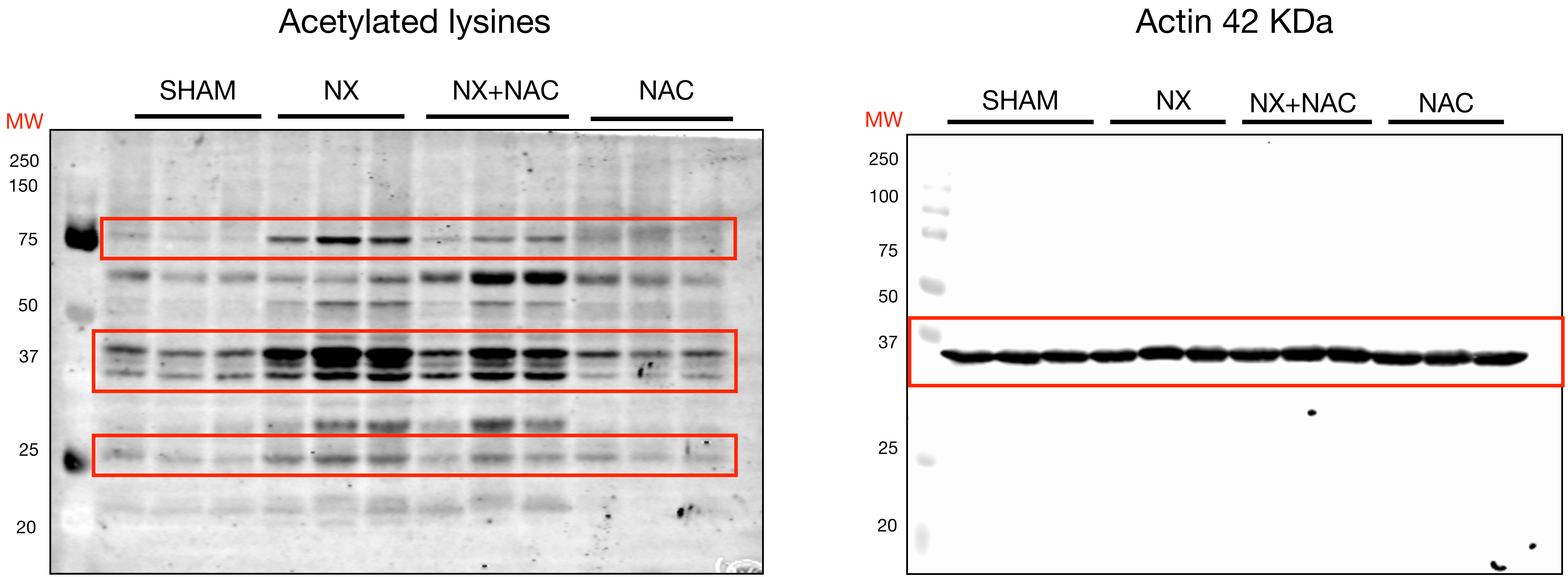

Acetylated lysines in mitochondria

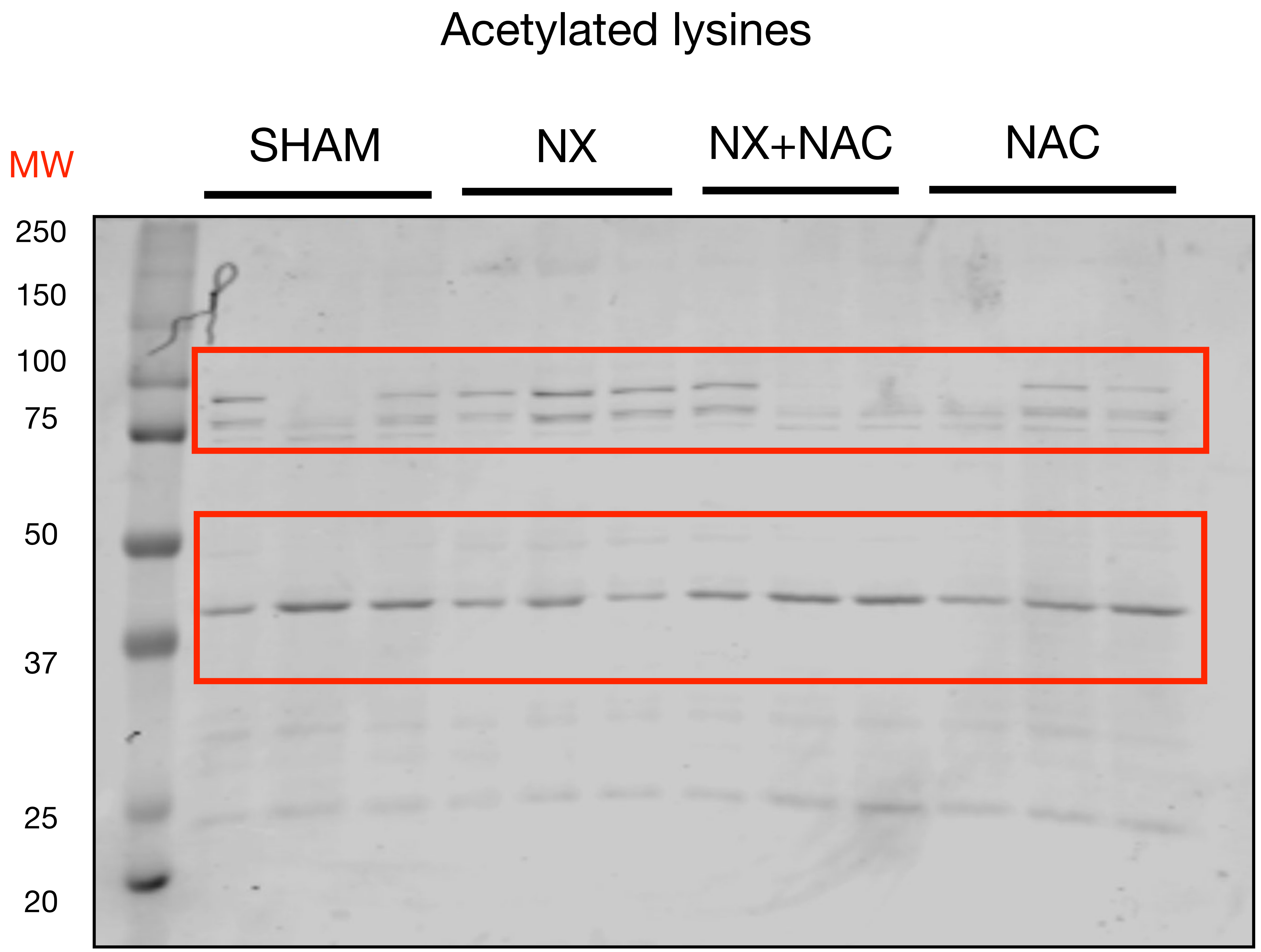

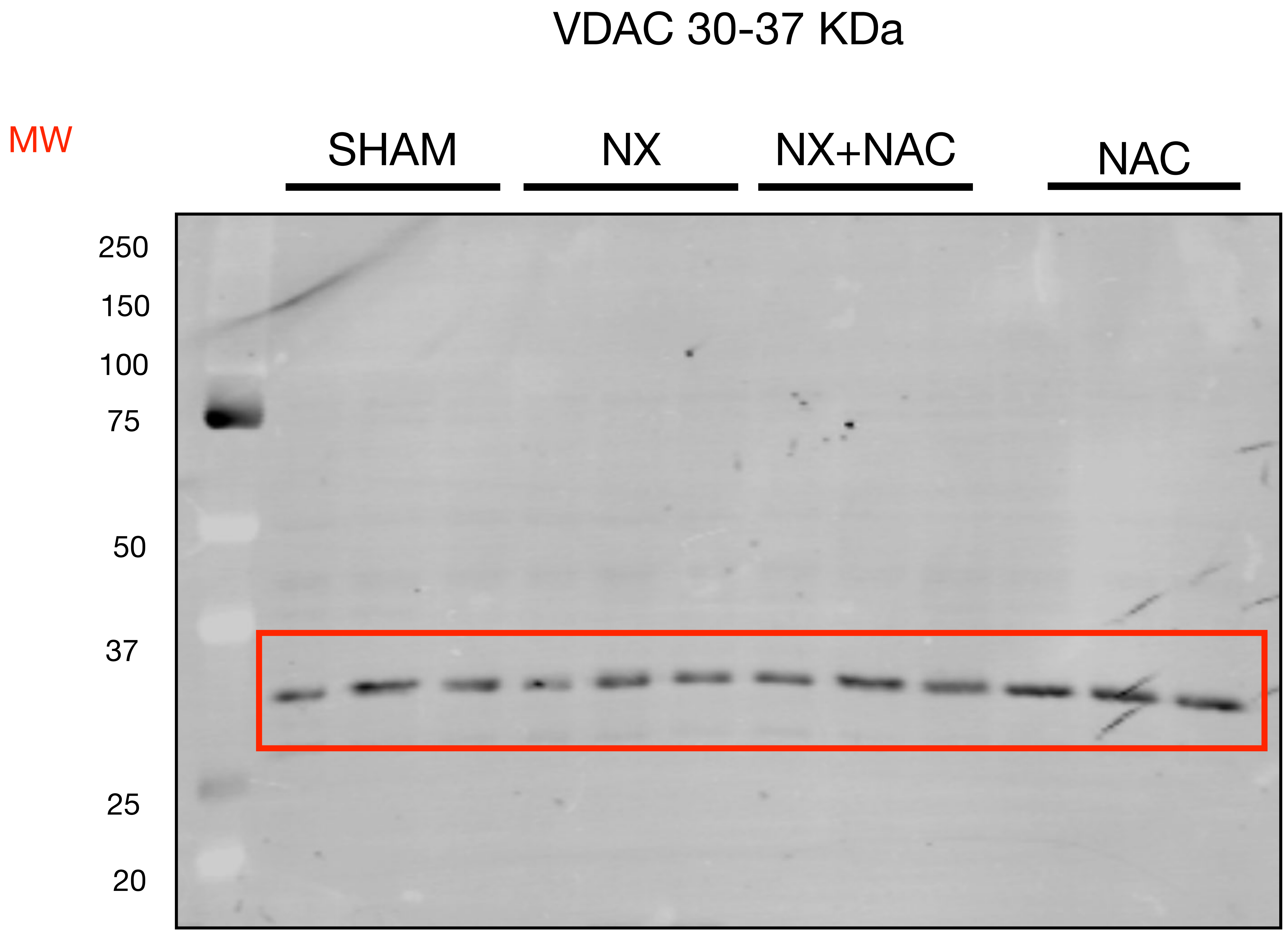

SOD 2

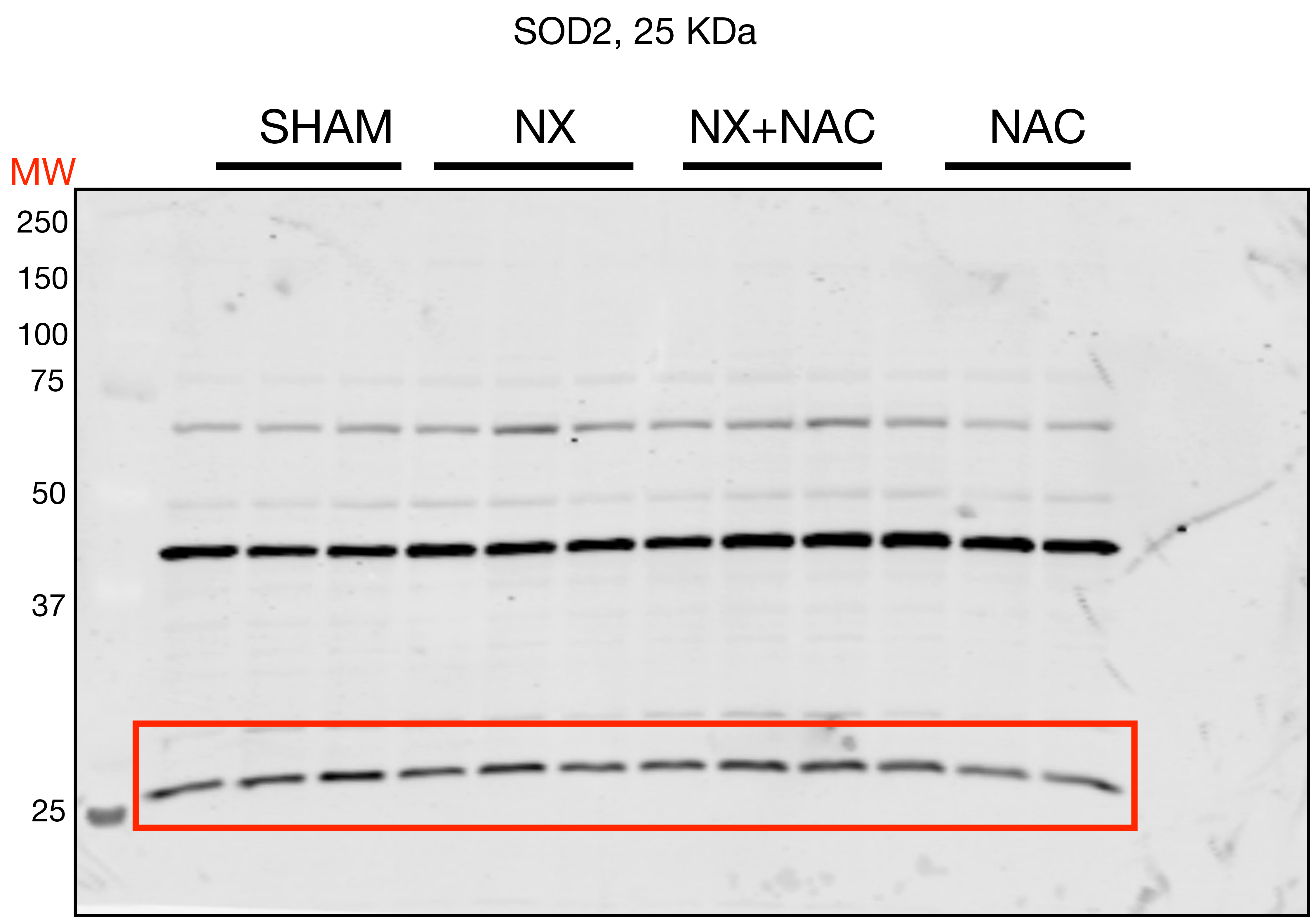
SOD2, 25 KDa
SHAM
NX
NX+NAC
NAC
MW
250
150
100
75
50
37
25

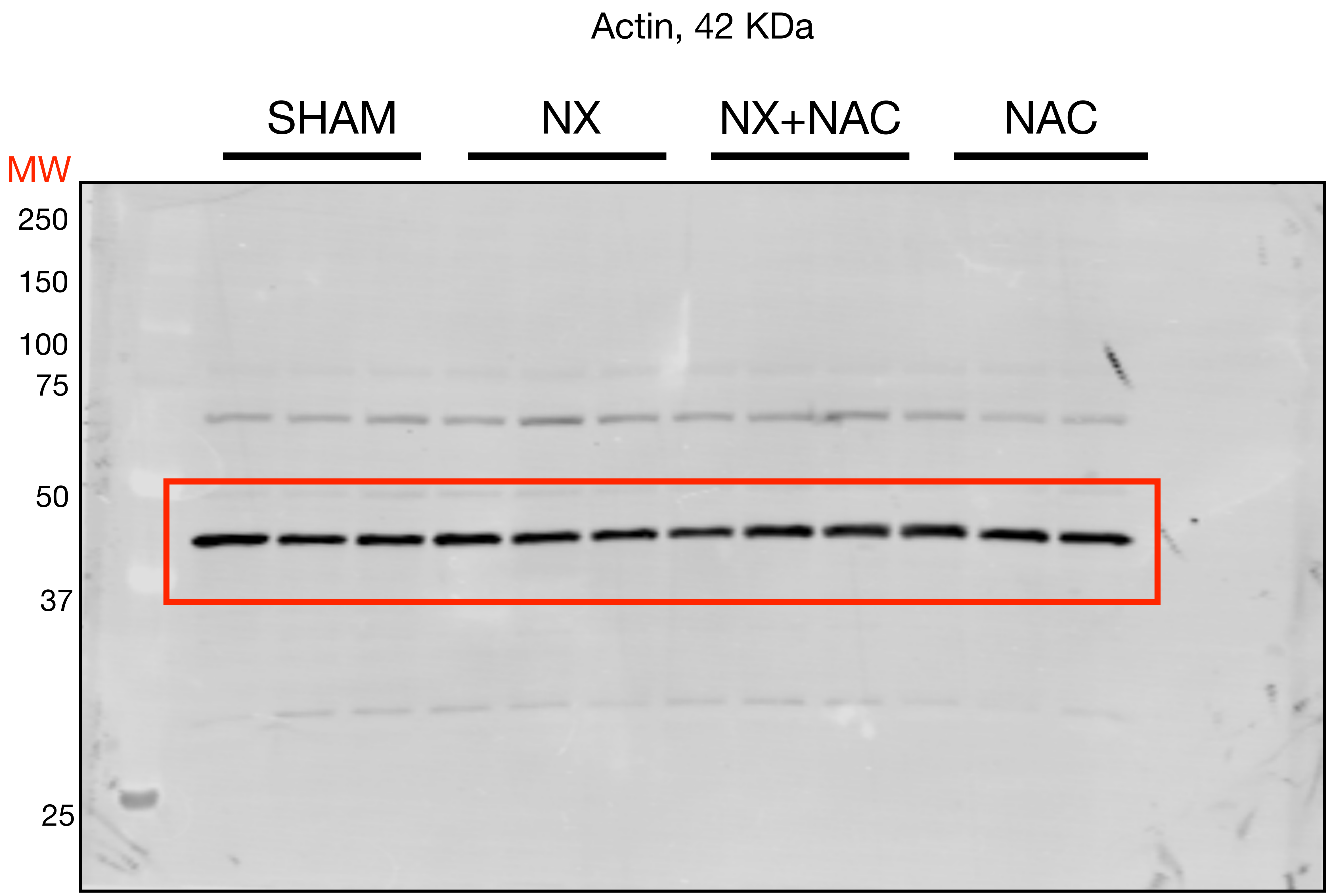
Actin, 42 KDa
SHAM
NX
NX+NAC
NAC
MW
250
150
100
75
50
37
25
