# Supplementary material for: Effect of N-Acetylcysteine in Mitochondrial Function, Redox Signaling, and Sirtuin 3 Levels in the Heart During Cardiorenal Syndrome Type 4 Development
Source: Antioxidants (Basel). 2025 Mar 20;14(3):367. doi: 10.3390/antiox14030367 (PMC11939543; doi:10.3390/antiox14030367)
Supplement: Supplementary file 1 [file antioxidants-14-00367-s001.zip › Supplementary Tables _ANTIOXIDANTS.pdf]

Supplementary Table S1. List of primary antibodies used for determination of proteins by western blotting.

Antibody (Ab)	Full name	Catalogue number	Source	Dilution of primary Ab	Manufacturer
**Anti-Troponin I**	Troponin I	Ab8295	Mouse	1:3000	Abcam
**Anti-IL-1β**	Interleukin-1β	503502	Armenian Hamster	1:3000	BioLegend
**Anti-SOD2**	Superoxide dismutase 2	D3X8F	Rabbit	1:3000	Cell Signaling Technology
**Anti-GCLC**	Glutamate-cysteine ligase catalytic subunit	HPA036359	Rabbit	1:500	Abcam
**Anti-GCLM**	Glutamate-cysteine ligase modifier subunit	Ab126704	Rabbit	1:3000	Abcam
**Anti-GSH**	Glutathione	Ab19534	Mouse	1:1000	Abcam
**Anti-Phospho-AMPKα (Thr172)**	Phosphorylated AMP-activated protein kinase	2535	Rabbit	1:1000	Cell Signaling Technology
**AMPKα**	AMP-activated protein kinase	2532	Rabbit	1:3000	Cell Signaling Technology
**Anti-PGC-1α**	Peroxisome proliferator-activated receptor-gamma coactivator (PGC)-1alpha	AB3242	Rabbit	1:2000	Sigma-Aldrich
**Anti-Acetylated lysines**	Acetylated lysines	9441	Rabbit	1:3000	Cell Signaling Technology
**Anti-OXPHOS**	Oxidative phosphorylation	Ab110413	Mouse	1:20,000	Abcam
**Anti-SIRT3**	Silent information regulator protein 3	D22A33	Rabbit	1:3000	Cell Signaling Technology
**Anti-Actin**	Actin	GTX109639	Rabbit	1:5000	Genetex
**Anti-**	Glyceraldehyde	Sc-25778	Rabbit	1:5000	Santa Cruz

**GAPDH**	3-phosphate dehydrogenase				Biotechnology
**Anti-VDAC**	Voltage-dependent anion channel	4866S	Rabbit	1:2000	Sigma-Aldrich

Sigma-Aldrich: St Louis, MO, USA.

Supplementary Table S2. List of fluorescent secondary antibodies used for Western blot.

Antibody	Full name	Catalogue	Source	Dilution	Purchased from
800CW	Anti-Rabbit IgG	926-32213	Donkey	1:15,000	Termofisher
800CW	Anti-Goat IgG	926-32214	Donkey	1:10,000	Termofisher
800CW	Anti-Mouse IgG	926-32212	Donkey	1:10, 000	Termofisher
680RD	Anti-Goat IgG	926-68074	Donkey	1:8,000	Termofisher
680RD	Anti-Rabbit IgG	926-68073	Donkey	1:8,000	Termofisher
680RD	Anti-Mouse IgG	926-68072	Donkey	1:8,000	Termofisher

Termofisher: Waltham, Massachusetts, USA.
